# A systematic review of the evaluation of endocrine-disrupting chemicals in the Japanese medaka (*Oryzias latipes*) fish

**DOI:** 10.3389/ftox.2023.1272368

**Published:** 2023-11-27

**Authors:** Asok K. Dasmahapatra, Charmonix B. Williams, Anitha Myla, Sanjay K. Tiwary, Paul. B. Tchounwou

**Affiliations:** ^1^ RCMI Center for Environmental Health, Jackson State University, Jackson, MS, United States; ^2^ Department of BioMolecular Sciences, School of Pharmacy, University of Mississippi, University, MS, United States; ^3^ RCMI Center for Urban Health Disparities Research and Innovation, Morgan State University, Baltimore, MD, United States

**Keywords:** Japanese medaka, endocrine disruptors, EATS pathways, systematic review, risk assessment

## Abstract

Japanese medaka (*Oryzias latipes*) is an acceptable small laboratory fish model for the evaluation and assessment of endocrine-disrupting chemicals (EDCs) found in the environment. In this research, we used this fish as a potential tool for the identification of EDCs that have a significant impact on human health. We conducted an electronic search in PubMed (http://www.ncbi.nlm.nih.gov/pubmed) and Google Scholar (https://scholar.google.com/) using the search terms, Japanese medaka, *Oryzias latipes*, and endocrine disruptions, and sorted 205 articles consisting of 128 chemicals that showed potential effects on estrogen–androgen–thyroid–steroidogenesis (EATS) pathways of Japanese medaka. From these chemicals, 14 compounds, namely, 17β-estradiol (E2), ethinylestradiol (EE2), tamoxifen (TAM), 11-ketotestosterone (11-KT), 17β-trenbolone (TRB), flutamide (FLU), vinclozolin (VIN), triiodothyronine (T3), perfluorooctanoic acid (PFOA), tetrabromobisphenol A (TBBPA), terephthalic acid (TPA), trifloxystrobin (TRF), ketoconazole (KTC), and prochloraz (PCZ), were selected as references and used for the identification of apical endpoints within the EATS modalities. Among these endpoints, during classification, priorities are given to sex reversal (masculinization of females and feminization of males), gonad histology (testis–ova or ovotestis), secondary sex characteristics (anal fin papillae of males), plasma and liver vitellogenin (VTG) contents in males, swim bladder inflation during larval development, hepatic vitellogenin (*vtg*) and choriogenin (*chg*) genes in the liver of males, and several genes, including estrogen–androgen–thyroid receptors in the hypothalamus–pituitary–gonad/thyroid axis (HPG/T). After reviewing 205 articles, we identified 108 (52.68%), 46 (22.43%), 19 (9.26%), 22 (17.18%), and 26 (12.68%) papers that represented studies on estrogen endocrine disruptors (EEDs), androgen endocrine disruptors (AEDs), thyroid endocrine disruptors (TEDs), and/or steroidogenesis modulators (MOS), respectively. Most importantly, among 128 EDCs, 32 (25%), 22 (17.18%), 15 (11.8%), and 14 (10.93%) chemicals were classified as EEDs, AEDs, TEDs, and MOS, respectively. We also identified 43 (33.59%) chemicals as high-priority candidates for tier 2 tests, and 13 chemicals (10.15%) show enough potential to be considered EDCs without any further tier-based studies. Although our literature search was unable to identify the EATS targets of 45 chemicals (35%) studied in 60 (29.26%) of the 205 articles, our approach has sufficient potential to further move the laboratory-based research data on Japanese medaka for applications in regulatory risk assessments in humans.

## 1 Introduction

Due to the increase in industrial and agricultural activities, endocrine-disrupting chemicals (EDCs), defined by the World Health Organization (WHO) as “Exogeneous substances that alter function(s) of the endocrine system and consequently cause adverse health effects in an intact organism or its progeny, or (sub)populations,” are accumulated in the environment. A strategic approach to identify EDCs would be utilized by the existing knowledge to prioritize and focus on the screening and environmental monitoring efforts of these chemicals. The European Commission also set criteria for the identification of EDCs that require regulatory action. Currently, endocrine disruptors (EDs) are identified on a case-by-case basis using the available guidance provided in the OECD Guidance Document 150 (2018). The OECD Conceptual Framework for Testing and Assessment of EDs provided a tiered framework for the organization of study information to assess endocrine activity. This framework provides guidance for prioritizing relevant data streams and methods according to the type and level of information needed for a regulatory assessment. In the USA, EPA’s EDSP has developed the requirements for the prioritization, screening, and testing of environmental contaminants, including pesticides, commercial chemicals, and agricultural products, for their potential to impact the endocrine system, especially in relation to estrogen, androgen, and thyroid (EAT) hormones and their nuclear receptors (NIEHS, 2018). Moreover, the perturbation of the enzymes of steroidogenesis by EDCs has potential effects on EAT pathways. Therefore, a two-tier testing approach was designed by EDSP. Tier 1 assays detect the potential effects of a chemical by various modes of action (Tier 1: screening) on EATS pathways. The results of the Tier 1 assays are evaluated by using a “weight of evidence” approach to determine whether the potential of the chemical is to interact with EATS and whether a Tier 2 assay is necessary. The purpose of Tier 2 studies is to use *in vivo* testing to further characterize the EATS effects and establish a dose–response relationship for adverse effects produced by the chemicals. Tier 2 tests are much longer-term studies that include exposure during critical life stages and have a broad range of more tightly spaced treatment than Tier 1. Moreover, Tier 2 tests can encompass multiple generations, covering effects on fecundity and fertility, development, growth, and sexual maturity. The successful completion of Tier 2 testing provided information to establish exposure and effect relationships, and assessed relevant endpoints across most life stages.

In aquatic environments, fish are considered one of the primary risk organisms for EDCs, especially those interacting with reproductive hormones. Sex determination in fish is very labile and can be disrupted or functionally reversed by external agents at critical developmental stages (Francis, 1992). Fish populations are directly exposed to a wide variety of EDCs, originating from industrial, agricultural, or municipal effluents (Ternes et al., 1999; Chen et al., 2007; Kim et al., 2014a). Evidence shows that EDCs can have long-term effects on reproduction and subsequent population development in natural fish populations (Kidd et al., 2007). The effects of EDCs on nuclear receptors have been studied extensively in small fish models like zebrafish, Japanese medaka, stickleback, and roach (*Rutilus rutilus*) (Iguchi et al., 2006; Lange et al., 2009; Tohyama et al., 2015). Since endocrine disruptions are linked to the receptor level, to predict ED effects, the identification of appropriate biomarkers at molecular levels is necessary.

Japanese medaka (*Oryzias latipes*) fish are small, freshwater teleost fish that inhabit gently flowing rivers and waterways. Like zebrafish (*Danio rerio*) and fathead minnows (*Pimephales promelas*), it is one of the small fish models (vertebrate) used in EDC studies (OECD, 2018). The sex determination locus has been identified in this fish species, and external sex-specific markers (chromatophores, shape of the anal and dorsal fins, anal fin papillae) can be used to easily differentiate males from females both from phenotypic and genotypic standpoints (Scholz and Mayer, 2008). Several OECD test guidelines (OECD TG 229; OECD TG 240) were used during the evaluation of EDCs in Japanese medaka, following tier-based approaches (Tier 1 and Tier 2). Moreover, the effects of endocrine active chemicals on Japanese medaka were reviewed previously (Urushitani et al., 2007; Flynn et al., 2017; Onishi et al., 2021; Kawashima et al., 2022). Based on the available publications found in public databases, we hypothesized that a literature search can identify the number and sources of EDCs that disrupted the EATS-related pathways of Japanese medaka (*O. latipes*) and correlate the effects with specific receptors at the molecular level.

In this review, we summarized the data on EDCs available in public databases, highlighting the links between molecular, phenotypic, and physiological endpoints using Japanese medaka as a single fish species. Although majority of the data refer to interfering with reproductive and thyroid hormone signaling pathways (EATS), limited information about the disruption of other endocrine organs, like the endocrine pancreas and interrenal gland (fish homolog of the adrenal gland), is also available (Dasmahapatra and Tchounwou, 2022a; b, 2023a; b). We evaluated the selective effects of 128 EDCs reported in 205 articles. As a result, we believed that 43 of them (EDCs) show potential to proceed to Tier 2 tests, and 13 chemicals should be considered EDCs without any further tier-based studies.

## 2 Materials and methods

### 2.1 Literature search strategy

**FIGURE 1:**
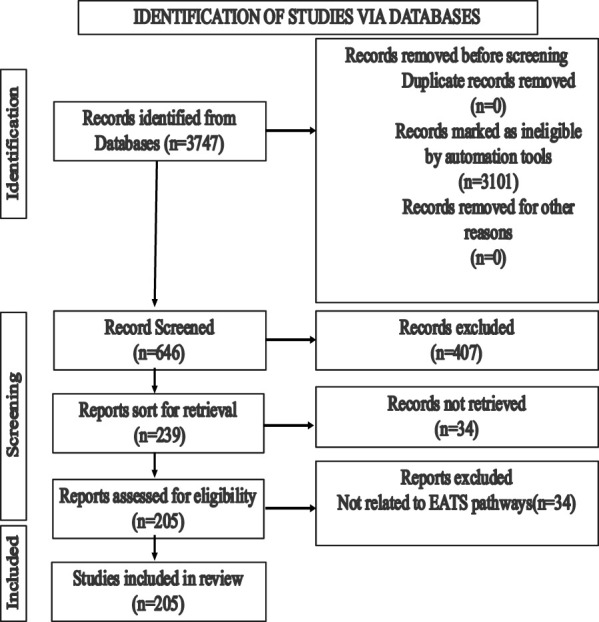
Strategies for the selection of literature reports from peer-reviewed articles published on Japanese medaka (*Oryzias latipes*).

**FIGURE 2:**
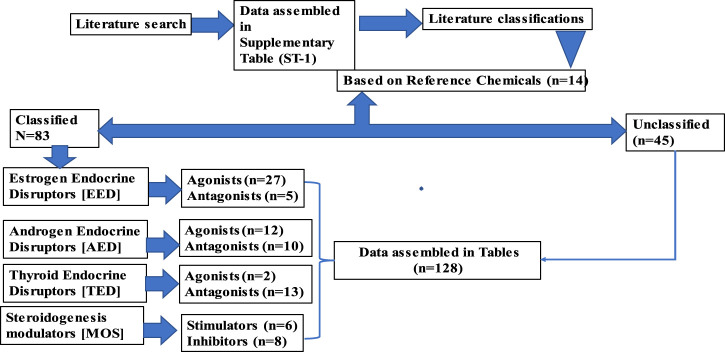
Strategies for the selection of chemicals from peer-reviewed articles published on Japanese medaka (*O. latipes*).

The objectives of the literature search were to identify the relevant studies published in peer-reviewed journals that focused on the endocrine disruption of Japanese medaka (*O. latipes*) induced by various chemicals detected in the aquatic environments. The search was performed in PubMed (http://www.ncbi.nlm.nih.gov/pubmed) and Google Scholar (https://scholar.google.com/). PubMed was considered the main and reliable source of information; however, Google Scholar was used if the full text article was not available in PubMed. We initiated our search in PubMed using the search term Japanese medaka (*Oryzias latipes*), which provided 3,747 results (until 30 June 2023). We narrowed down the search by adding the term “endocrine” (Japanese medaka, *Oryzias latipes*, and endocrine), which reduced the number to 646, and finally, the addition of the term “endocrine disruption” reduced the results to 239 (Figure 1). We finally sorted 205 articles for review that focused on EATS pathways of Japanese medaka (Figure 1). We identified 128 chemicals that have potential ED effects on this fish (Japanese medaka, *O. latipes*) (Figure 2). After a literature search, we assembled ED-related information in Supplementary Table S1, which was also deposited in Figshare (doi/10.6084/mg.figshare. 22598068). For classification of these compounds as selective disruptors of EATS pathways, 14 chemicals from 128 searched chemicals were selected as reference chemicals (Figure 2; Table 1). For estrogen endocrine disruptors (EEDs), E2 and EE2 were used as reference chemicals for agonists, and TAM was used for antagonists. For androgen endocrine disruptors (AEDs), 11-KT and TRB were used for agonists, and FLU and VIN were used for antagonists. For thyroid endocrine disruptors, (TEDs), T3 was used for agonists, and PFOA and TBBPA were used for antagonists. For steroidogenesis, TPA and TRF were used for stimulators, and KTC and PCZ were used for inhibitors (Table 1). After critical evaluation of the ED effects of these reference chemicals, the criteria of evaluation of endocrine disruption induced by an EDC on Japanese medaka are determined (Table 1). The chemicals which were unable to fulfill the criteria were considered unclassified.

**TABLE 1:** The apical endpoints of the reference chemicals related to EATS pathways in Japanese medaka.

Endocrine targets	Reference chemicals	Agonists/Antagonists/Stimulator/Inhibitor	Affinity	Literature	End points	
					Agonists	Antagonists
EED	E2	Agonist	*In vitro* reporter gene assay (affinity) (Tohyama et al., 2015)i) *esr1*: EC50 = 1.31 × 10^−10^ Mii) *esr1*: EC50 = 1.31 × 10^−10^ Miii) *esr2b*: EC50 = 8.16 × 10^−11^ M	Nimrod and Benson (1998), Patyna et al. (1999), Foran et al. (2000), Koger et al. (2000), Shioda and Wakabayashi (2000), Metcalfe et al. (2001), Tabata et al. (2001), Kang et al. (2002a), Kashiwada et al. (2002), Oshima et al. (2003), Balch et al. (2004b), Hall et al. (2005), Zeng et al. (2005), Balch and Metcalfe (2006), Hirai et al. (2006), Zhang et al. (2008d), Sun et al. (2009), Jin et al. (2011a), Kamata et al. (2011), Hirakawa et al. (2012), Lee et al. (2012), Flynn et al. (2013), Lei et al. (2013), Green et al. (2015), Tohyama et al. (2015), Inagaki et al. (2016), Flynn et al. (2017), Lee et al. (2017a), Lee Pow et al. (2017), Bertotto et al. (2019), Kang et al. (2019), Myosho et al. (2019), Ishibashi et al. (2020), Spirhanzlova et al. (2020), Onishi et al. (2021), Pandelides et al. (2021), Horie et al. (2022a).	1. Female biased sex ratio, testis-ova (male feminization)	
					2. Significant decrease in fecundity	
					3. Serum VTG level increased in males and females	
					4. Inhibition of swim bladder inflation	
					5. Intersex gonad	
					6. Increased HSI in males	
					7. Secondary sexual features reduced	
					8. *gsdf* expression in XY embryos remained unaltered	
					9. Vacuolization of hepatocytes in the liver	
					10. Hydropic degeneration in glomerulus of the kidney	
					11. Reproductive behavior suppressed with in both sexes	
					12. No effect on male sexual behavior	
					13. Histological structure of the kidney disrupted	
					14. Gene expression	
					A: Brain:	
					(i) Male:	
					1. Upregulation of *gnrh1*, *cyp19b*, *esr1*, *fshβ*, *lhβ*	
					2. Downregulation of *gnrhR1*, *gnrhR2*, *arα*	
					(ii) Female:	
					1. Upregulation of *esr1* and *esr2a*	
					2. *cyp19b* remained unaltered	
					B. Liver:	
					(i) Male:	
					1. Upregulation of *esr1*, *vtg 1*, *vtg 2*, *chgH*, *chgHm*, *chgL*, *cyp1c*	
					2. No alteration in *esr1*, *esr2a*, and *arα*	
					(ii) Female:	
					1. *vtg1* and *vtg2* remained unaltered	
			*In vitro* reporter gene assay (Onishi et al., 2021): EC50 = 0.00098 µM (medaka esr1); IC50 = 2.0 µM (medaka arβ)		2. *esr2a* and *arα* remained unaltered	
					C. Gonad	
					i) Testis:	
					1. Aromatase expression increased	
					2. DNA methylation pattern reduced	
					3. *esr1* transcripts decreased	
					4. Downregulation of *cyp11a*, *cyp17*	
					5. *fshR* reduced	
					(ii) Ovary:	
					1. Downregulation of *cyp19a*	
	EE2	Agonist	*In vitro* reporter gene assay: EC50 = 0.00088 µM (Medaka *esr1* agonist assay); IC50 = 0.14 µM (medaka *arβ* antagonist assay (Onishi et al., 2021)	Scholz and Gutzeit (2000), Metcalfe et al. (2001), Foran et al. (2002), Islinger et al. (2002), Lee et al. (2002), Seki et al. (2002), Balch et al. (2004a), Nozaka et al. (2004), Chikae et al. (2004), Hano et al. (2005), Zeng et al. (2005), Orn et al. (2006), Zhang et al. (2008c), Hashimoto et al. (2009), Park et al. (2009), Sun et al. (2011a), Hirakawa et al. (2012), Liao et al. (2014), Schiller et al. (2014), Abdel-Moneim et al. (2015), Bhandari et al. (2015), Bhandari et al. (2020), Thayil et al. (2020), Onishi et al. (2021), Pandelides et al. (2021), Horie et al. (2022a), Myosho et al. (2022)		
	TAM	Antagonist	*In vitro* reporter gene assay: IC50 = 0.14 µM (medaka *esr1* antagonist assay; (Onishi et al., 2021)	Chikae et al. (2004), Sun et al. (2007a), Sun et al. (2011a), Flynn et al. (2017), Onishi et al. (2021).		1. Significant reduction in fecundity
						2. Reduction in hatchability, hatching delay, developmental abnormalities
						3. Liver VTG in male increased, reduced in females
						4. Liver histology of male fish disrupted
						5. HSI remained unaltered
						6. Secondary sexual features reduced
						A: Brain:
						i) Male:
						1. Upregulation of *arα*, *esr1* and *cyp19a*
						2. Downregulation of *cyp19b*
						ii) Female:
						1. No alteration in *esr1* and *esr2*
						2. Upregulation of *arα* and *cyp19a*
						3. Downregulation of *cyp19b*
						B: Liver:
						i) Male:
						1. Upregulation of *vtg1*, vtg*2*, esr1, *arα*
						2. No alteration in *esr2a*
						ii) Female:
						1. Upregulation of *esr2a*
						2. Downregulation of *vtg1* and *vtg2* and esr1
						3. *arα* remained unaltered
						C; Gonad
						i) Testis:
						1. Upregulation of *StAR*, *cyp19b*
						2. Downregulation of *esr1* and *esr2a*, *cyp17a*, *cyp17b*, *cyp19a*
						3. *cyp11a*, *cyp11b* remained unaltered
						ii) Ovary:
						1. Upregulation of *StAR*, *cyp11a*
						2. Downregulation of *esr1*, *esr2a* and *arα*
						3. *cyp11b* remained unaltered
AED	11-KT	Agonist	*In vitro* reporter gene assay: IC50 = 0.0027 (medaka *arβ* agonist assay; Onishi et al., 2021)	Asahina et al. (1989), Leon et al. (2007), Leon et al. (2008), Grillitsch et al. (2010), Onishi et al. (2021), Watanabe et al. (2023).	1. Increased anal fin papillary processes (masculinization in females)	
					2. Enhancement of growth is sex-specific (males are larger than females)	
					3. Hypertrophy in thyroid follicular cells induced in both sexes	
					4. Germ cell necrosis is induced in both sexes	
					5. Male biased sex-ratio	
					6. Upregulation of *gsdf* mRNA in XX embryos (sex reversal of the XX fish)	
					7. Ovo-testis	
					8. Decrease in VTG content in females	
					9. Gene Expression	
					A: Brain:	
	TRB	Agonist	*In vitro* reporter gene assay: EC50 = 0.0036 µM(medaka *arβ* agonist assay; Onishi et al., 2021)	Orn et al. (2006), Seki et al. (2006), Zhang et al. (2008b), Park et al. (2009), Grillitsch et al. (2010), Flynn et al. (2013), Flynn et al. (2017), Abdel-Moneim et al. (2015), Mazukami-Murata et al. (2016), Kang et al. (2019), Onishi et al. (2021), Horie et al. (2022a), Myosho et al (2022).	i) Female	
					1. Upregulation of *gnrhR2*, *cyp19b*	
					B: Liver:	
					1. down regulation of *vtg1*, *vtg2*, *chgH*, *chgHm* mRNAs in the both sexes	
					2. Down regulation of *esr1* in males	
					3. Upregulation of *cyp3A* and *annexin max2* in females	
					C; Gonad	
					(i) Testis:	
					1. Down regulation of *StAR* and *cyp11b*	
					(ii) Ovary:	
					1. Upregulation of *cyp19a*	
					2. Upregulation of *a*	
	FLU	Antagonist	*In vitro* reporter gene assay: IC50 = 12 µM (medaka *arβ* agonist assay; Onishi et al. (2021), Horie et al. (2022a)	Chikae et al. (2004), Nozaka, (2004), Kang et al. (2006), Leon et al. (2007), Leon et al. (2008), Nakamura et al. (2014b), Schiller et al. (2014), Onishi et al. (2021).		1. Increased plasma VTG levels in females, not in males
						2. Hepatic VTG unaltered in males, decreased in females
						3. Fecundity and fertility were significantly decreased
						4. Growth (length and weight) was inhibited in males, not in females (sex-specific)
						5. No sex reversal
						6. Formation of testis-ova; disruption of spermatogenesis and ovarian cell necrosis
	VIN	Antagonist	*In vitro* reporter gene assay: IC50 = 5.1 µM(medaka *arβ* antagonist assay; Onishi et al., 2021).	Kiparissis et al. (2003b), Nakamura et al. (2014b), Sun et al. (2016b), Flynn et al. (2017), Onishi et al. (2021)		7. Hypertrophy of thyroid follicular cells in males
						8. Decrease in papillary process in the anal fin of male fish; females did not develop papillary process in the anal fin
						9. Reduced expression of *gnrhr2*, *cyp11b* and *3βhsd*
						10. Repressed *esr2a* and *cyp19a1b*
						11. No significant induction of *gsdf* expression in XY embryos
						12. Gene expression:
						A: Brain:
						(i) Male:
						1. Upregulation of *esr2a*, *arα* and *cyp19a* and *cyp19b* genes
						B: Testis:
						1*. esr1* and *cyp17b* mRNAs were upregulated
						2*. cyp19a* and *cyp19b* downregulated
TED	T3	Agonist		Godfrey et al. (2019), Horie et al. (2022d)	1. Decrease in the surface area of swim bladder in females	
					2. Upregulation of *trα* and *trβ* mRNAs	
	PFOA	Antagonists		Ji et al. (2008), Lee et al. (2017b), Kang et al. (2019), Godfrey et al. (2019)		1. Fecundity suppressed
						2. Females displayed larger swim bladder
						3. Thyroid follicles showed hyperplasia, hypertrophy, and colloidal depletion
						4. No change in sex ratio
	TBBPA	Antagonists		Horie et al. (2023a)		5. In liver, *vtg1* in males, and *vtg1* and *vtg2* in females increased; VTG protein in males reduced
						6. *chgH* and *chgHm* mRNA expression in liver of males
						7. Increased *tshβ*, *trβ* and *vtg* in females
						8. Upregulation of *esr2*a and *vtg* in males
						9. No effect on expression of *dio1* and *dio2*
MOS	TPA	Stimulator		Jang and Ji (2015).	1. Upregulation of *cyp19a*, *cyp19b*, *StAR*, and *cyp17* mRNAs in a concentration-dependent manner	
					2. *erα*, *vtg1*, *vtg2*, *cyp11a*, *hsd3b. trα*, *dio2*, *ahr* remained unaltered	
	TRF	Stimulator		Zhu et al., (2015)		
	KTC	Inhibitor	In vitro reporter gene assay: IC50 = 4.2 µM (medaka *arβ* antagonist assay) (Onishi et al., 2021)	Zhang et al. (2008a), Onishi et al. (2021)		1. Reduced fecundity
						2. Anal fin papillae increased in males not in females
						3. Liver VTG decreased in both sexes
						4. Gene expression
	PCZ	Inhibitor		Zhang et al. (2008a), Schiller et al. (2014), Flynn et al. (2017), Kang et al. (2019), Onishi et al. (2021)		A: Brain:
						(i) Male:
						1. Downregulation of *cyp19b*
						(ii) Female:
						1. Downregulation of *gnrhR2* and *gnrhR3*
						B: Liver:
						(i) Male
						1. Upregulation of *esr1* and *arα* mRNAs
						2. Downregulation of *chgH* and *chgHm*
						(ii) Female
						1. Upregulation of *arα*
						2. Downregulation of *esr1*, *vtgI*, *vtg2*, *cghL*, *chgH*, *chgHm*
						C: Gonad:
						(i) Testis:
						1. Upregulation of *esr2a*, *lhr*, *cyp19a*
						(ii) Ovary:
						1. Downregulation of *esr2*, *arα*, *lhr*
						2. Downregulation of *StAR*, *cyp11a*, *cyp11b*

### 2.2 Genes sensitive to EDCs within the EATS modalities of Japanese medaka

Within the EATS modalities, most of the EDCs function via the hormone-responsive element of a target gene by binding to the ligands of nuclear receptors (NRs), including ESRs (*esr1*, *esr2a,* and *esr2b*), ARs (*arα* and *arβ*), or TRs (*trα* and *trβ*). The effects of EDCs on NRs have been studied in Japanese medaka (Myosho et al., 2022; Tohyama et al., 2015). The expression of estrogen-responsive genes is known to be induced or suppressed by estrogen via ESRs with the estrogen-responsive elements (EREs) of responsive genes. Specifically, VTGs and CHGs encode complex precursor proteins in the egg yolk and eggshell, respectively, and are synthesized in the liver. EDCs with estrogenic potential induced the expression of VTG and CHG in juvenile and mature male fish, respectively, in which the expression levels of *vtg* and *chg* are typically low. Therefore, to evaluate the estrogenic potential of EDCs in Japanese medaka, *vtgs* (*vtg1* and *vtg2*) and *chg* (*chgL, chgH,* and *chgHm*) can be used as markers.

The androgenic effects of EDCs are mediated via direct binding to ARs (*arα* and *arβ*) with distinctive binding properties or transactivation activity (Onishi et al., 2021; Kawashima et al., 2022). Molecular effects of AEDs could be identified from secondary sex characteristics (anal fin papillae of males) or indirectly by analysis of the induction/suppression of VTG (*vtg1* and *vtg2*), LH, FSH, aromatase, ESRs, or T-hormone levels (Scholz and Mayers, 2008). The formation of papillary processes in the anal fin of Japanese medaka (males) is augmented by the bone morphogenic protein (*bmp7*) and lymphoid enhancer-binding factor (*lef1*), along with *arα* and *arβ* which can be used as markers for AEDs during evaluation (Ogino et al., 2014).

EDCs having TH-disrupting potential inhibit or accelerate TH-dependent processes, either directly or indirectly, including TH-dependent gene expression. The HPT axis is highly conserved among vertebrates, and the TH and receptors (*trα* and *trβ*) play crucial roles in the regulation of development, growth, and energy metabolism. A number of high-profile environmental pollutants adversely affect the TH system of Japanese medaka, including development, visual performance, malformation of the swim bladder, and TH-dependent gene (*tshβ*, *trα*, *trβ*, *dio1*, and *dio2*) expression (Godfrey et al., 2019; Dang et al., 2021; Horie et al., 2022c). Therefore, to evaluate the thyroid-disrupting potential of EDCs, in Japanese medaka, these genes (*tshβ*, *trα*, *trβ*, *dio1*, and *dio2*) can be used as markers for TEDs.

Moreover, within the estrogen–androgen–steroidogenesis (EAS) modalities, the steroid hormones, estrogen (E2) and androgen (A), are derived from cholesterol and secreted from the gonads (testis or ovary). The production, conversion, and breakdown of E2 and A in the endocrine glands and target tissues are carefully controlled by a range of steroidogenic enzymes (steroidogenesis), many of which belong to the cytochrome P450 family (CYP11, CYP17, and CYP19). Many EDCs have the abilities to disrupt the synthesis and function of steroidogenic enzymes, resulting in inappropriate concentrations of E2 or A, which impacts the reproduction, development, growth, and metabolism of fish (Japanese medaka). The enzyme aromatase (CYP19) converts testosterone/androgen (A) into estradiol/estrogen (E) and controls the fine balance between these two potent sex steroids. Therefore, the genes that show potential to regulate steroidogenesis in Japanese medaka are used as markers during EDC evaluation.

## 3 Results

Depending on the ED effects, we sorted 205 articles (Table 2) consisting of 128 chemicals (1.6 articles/chemicals or the approximate ratio is 8 articles:5 chemicals) that showed potential effects on Japanese medaka. Furthermore, based on the apical endpoints selected from 14 reference chemicals (Table 1) and after reviewing 165 articles, we identified 83 chemicals that target EATS pathways of Japanese medaka (Tables 3–10), and due to the lack of sufficient information, 45 chemicals reviewed from 60 articles remained unclassified (Table 11). Moreover, among the 83 chemicals that target EATS pathways, 43 chemicals were recommended for Tier 2 tests, and 13 chemicals show enough potential to be considered EDCs without any further tier-based studies (Tables 3–10). The rest of the EATS chemicals need further studies on Tier 1 screening. Moreover, with regard to the apical endpoints, the EATS chemicals were further classified as agonists and antagonists of EEDs, AEDs, and TEDs, and stimulators or inhibitors of steroidogenesis (Figure 2; Tables 3–10).

**TABLE 2:** Literature reports sorted for the evaluation of the effects of EDCs on Japanese medaka (*Oryzias latipes*).

	Literature author	EED	AED	TED	MOS	Unclassified
1	Abdel-Moneim et al. (2015)					
2	Asahina et al. (1989)					
3	Asala et al. (2021)					
4	Asala et al. (2022)					
5	Balch et al. (2004a)					
6	Balch et al. (2004b)					
7	Balch and Metcalfe (2006)					
8	Beltran et al. (2022)					
9	Bertotto et al. (2019)					
10	Bhandari et al. (2015)					
11	Bhandari et al. (2020)					
12	Cheek et al. (2001)					
13	Chen et al. (2022)					
14	Chikae et al. (2004)					
15	Chu et al. (2016)					
16	Coronado et al. (2008)					
17	Dasmahapatra et al. (2020a)					
18	Dasmahapatra et al. (2020b)					
19	Dasmahapatra and Tchounwou (2022a)					
20	Dasmahapatra and Tchounwou (2022b)					
21	Dasmahapatra and Tchounwou (2023a)					
22	Dasmahapatra and Tchounwou (2023b)					
23	Devoy et al. (2023)					
24	Edmunds et al. (2000)					
25	Flippin et al. (2007)					
26	Flynn et al. (2013)					
27	Flynn et al. (2017)					
28	Flynn et al. (2018)					
29	Foran et al. (2000)					
30	Foran et al. (2002)					
31	Foran et al. (2004)					
32	Fujita et al. (2022)					
33	Godfrey et al. (2019)					
34	Gonzalez-Doncel et al. (2016)					
35	Gonzalez-Doncel et al. (2014a)					
36	Gonzalez-Doncel et al. (2014b)					
37	Gonzalez-Doncel et al. (2017)					
38	Gray and Metcalfe (1997)					
39	Gray et al. (1999a)					
40	Gray et al. (1999b)					
41	Green et al. (2015)					
42	Grillitsch et al. (2010)					
43	Gronen et al. (1999)					
44	Hall et al. (2005)					
45	Hall et al. (2007)					
46	Hamm and Hilton, (2000)					
47	Han et al. (2010)					
48	Hano et al. (2005)					
49	Hano et al. (2007)					
50	Hashimoto et al. (2009)					
51	Hirai et al. (2006)					
52	Hirakawa et al. (2012)					
53	Hirako et al. (2017)					
54	Hishida and Kawamoto. (1970)					
55	Hong et al. (2007)					
56	Horie et al. (2017)					
57	Horie et al. (2018)					
58	Horie et al. (2019)					
59	Horie et al. (2021)					
60	Horie et al. (2022a)					
61	Horie et al. (2022b)					
62	Horie et al. (2022c)					
63	Horie et al. (2022d)					
64	Horie et al. (2023a)					
65	Horie et al. (2023b)					
66	Horng et al. (2010)					
67	Inagaki et al. (2016)					
68	Inui et al. (2003)					
69	Ishibashi et al. (2004)					
70	Ishibashi et al. (2006)					
71	Ishibashi et al. (2020)					
72	Islinger et al. (2002)					
73	Jang and Ji (2015)					
74	Ji et al. (2008)					
75	Ji et al. (2010)					
76	Ji et al. (2012)					
77	Jin et al. (2011a)					
78	Jin et al. (2011b)					
79	Jin et al. (2020)					
80	Kamata et al. (2011)					
81	Kang et al. (2002a)					
82	Kang et al. (2002b)					
83	Kang et al. (2003)					
84	Kang et al. (2006)					
85	Kang et al. (2008)					
86	Kang et al. (2019)					
87	Kashiwada et al. (2002)					
88	Kawashima et al. (2022)					
89	Kim et al. (2007)					
90	Kim et al. (2012)					
91	Kim et al. (2014)					
92	Kim et al. (2017)					
93	Kim et al. (2020)					
94	Kiparissis et al. (2003a)					
95	Kiparissis et al. (2003b)					
96	Knorr and Braunbeck (2002)					
97	Kobayashi et al. (2005)					
98	Koger et al. (2000)					
99	Kuhl and Brouwer., 2006					
100	Kwak et al. (2018)					
101	LaLone et al. (2013)					
102	Lee et al. (2002)					
103	Lee et al. (2011)					
104	Lee et al. (2012)					
105	Lee et al. (2013)					
106	Lee et al. (2014)					
107	Lee et al. (2017a)					
108	Lee et al. (2017b)					
109	Lee et al. (2019a)					
110	Lee et al. (2019b)					
111	Lee Pow et al. (2017)					
112	Lei et al. (2013)					
113	Lei et al. (2016)					
114	Leon et al. (2007)					
115	Leon et al. (2008)					
116	Li et al. (2016)					
117	Li et al. (2017)					
118	Li et al. (2019)					
119	Liang et al. (2020)					
120	Liao et al. (2014)					
121	Lin et al. (2014)					
122	Liu et al. (2018)					
123	Matten et al. (2023)					
124	Mazukami-Murata et al. (2016)					
125	Metecalfe et al. (2001)					
126	Mihaich et al. (2019)					
127	Miyagawa et al. (2014)					
128	Myla et al. (2021a)					
129	Myla et al. (2021b)					
130	Myosho et al. (2019)					
131	Myosho et al. (2022)					
132	Nair et al. (2017)					
133	Nakamura et al. (2014a)					
134	Nakamura et al. (2014b)					
135	Nakayama et al. (2011)					
136	Nimrod and Benson (1998)					
137	Nozaka et al. (2004)					
138	Ogino et al. (2014)					
139	Onishi et al. (2021)					
140	Orn et al. (2006)					
141	Oshima et al. (2003)					
142	Pandelides et al. (2021)					
143	Papoulias et al. (2000)					
144	Park et al. (2008)					
145	Park et al. (2009)					
146	Patyna et al. (1999)					
147	Paul et al. (2021)					
148	Powe et al. (2018)					
149	Richter et al. (2016)					
150	Robinson et al. (2017)					
151	Saunders et al. (2015)					
152	Schiller et al. (2013)					
153	Schiller et al. (2014)					
154	Scholz and Gutzeit (2000)					
155	Seki et al. (2002)					
156	Seki et al. (2003a)					
157	Seki et al. (2003b)					
158	Seki et al. (2004)					
159	Seki et al. (2006)					
160	Shioda and Wakabayashi (2000)					
161	Smith et al. (2019)					
162	Song et al. (2020)					
163	Spirhanzlova et al. (2017)					
164	Spirhanzlova et al. (2020)					
165	Su et al. (2023)					
166	Sun et al. (2007a)					
167	Sun et al. (2007b)					
168	Sun et al. (2009)					
169	Sun et al. (2011a)					
170	Sun et al. (2014)					
171	Sun et al. (2016a)					
172	Sun et al. (2016b)					
173	Sun et al. (2016c)					
174	Suzuki et al. (2004)					
175	Tabata et al. (2001)					
176	Tabata et al. (2004)					
177	Tagawa and Hirano (1991)					
178	Teather et al. (2005)					
179	Thayil et al. (2020)					
180	Thresher et al. (2011)					
181	Tilton et al. (2003)					
182	Tohyama et al. (2015)					
183	Uchida et al. (2010)					
184	Wagner et al. (2017)					
185	Wang et al. (2016)					
186	Watanabe et al. (2017)					
187	Watanabe et al. (2023)					
188	Yamamoto et al. (2007)					
189	Yamamoto et al. (2011)					
190	Yan et al. (2020)					
191	Yokota et al. (2000)					
192	Yokota et al. (2001)					
193	Yokota et al. (2005)					
194	Yokota et al. (2017)					
195	Yokota et al. (2018)					
196	Yum et al. (2010)					
197	Zeng et al. (2005)					
198	Zha et al. (2006)					
199	Zhang and Hu (2008)					
200	Zhang et al. (2008a)					
201	Zhang et al. (2008b)					
202	Zhang et al. (2008c)					
203	Zhang et al. (2008d)					
204	Zhang et al. (2008e)					
205	Zhu et al. (2015)					

Only the names of the authors are listed in the first column of the table. The cells filled in colors (red = EED; blue = AED; green = TED; yellow = MOS; black = unidentified pathways) represent the specific endocrine pathways/organs disrupted by EDCs. EDCs, endocrine-disrupting chemicals; EED, estrogen endocrine disruptor; AED, androgen endocrine disruptor; TED, thyroid endocrine disruptor; MOS, modulators of steroidogenesis.

**TABLE 3:** Potential EED agonist chemicals identified from the literature search.

Serial number	Name of the chemical	Nature (source)	Significant endpoints	Reference	Recommendation
1	AR-1260	Polychlorinated biphenyl (*persistent organic pollutant*)	1. Induced *vtg*, *chgL*, and *chgHm* mRNAs in the liver of males	Yum et al. (2010)	Tier 2 (thyroid-dependent mechanisms)
2	AZM	Organophosphate pesticide [*agricultural*]	1. Female-biased sex ratio	Teather et al. (2005)	Tier 1 (thyroid-dependent mechanisms)
3	BZP	Antimicrobial agent [*personal* *care product*]	1. Serum VTG enhanced in males	Yamamoto et al. (2007)	Tier 2 (thyroid-dependent mechanisms)
			2. *vtg1*, *vrg2*, *chgL*, *chgH*, *chgHm*, *esr1*, and *cyp1a* genes upregulated in the liver of males		
4	BMT	Synthetic glucocorticoid (*pharmaceuticals*)	1. Both *vtg1* and *vtg2* mRNAs were induced in the liver of male fish	Su et al. (2023)	Tier 2 (thyroid-dependent mechanisms)
			2. Ova found in the testis		
			3. Serum T reduced, while E2 induced		
5	BPA	Raw material for polycarbonate plastic [*industrial*]	1. Anal fin papillae in males disappeared	Shioda and Wakabayashi (2000)	(Thyroid-dependent mechanisms)
			2. Testis–ova	Yakota et al. (2000)	
			3. VTG mRNAs (*vtg1* and *vtg2*) and protein in the liver of males increased	Metcalfe et al. (2001)	
			4. Upregulation of *chgL* and *chgH* in the liver of male fish	Tabata et al. (2001)	
			5. Expression of *esr1*, *esr2a*, and *esr2b* genes remained unchanged	Kang et al. (2002b) Kashiwada et al. (2002) Lee et al. (2002) Zeng et al. (2005) Kamata et al. (2011) Lee et al. (2012) Schiller et al. (2014) Bhandari et al. (2015, 2020) Tohyama et al. (2015) Inagaki et al. (2016) Li et al. (2016); Li et al. (2017) Horie et al. (2019) Ishibashi et al. (2020) Thayil et al. (2020) Kawashima et al. (2022)	
6	CFR	Antibiotic (*pharmaceutical*)	1. Plasma E2 level increased in females	Kim et al. (2017)	Tier 2 (thyroid-dependent mechanisms)
			2. Sex-specific alteration in the gene expression pattern of the HPG axis		
7	CLT	Organochlorine pesticide (fungicide) [*agricultural*]	1. Female-biased sex ratio	Teather et al. (2005)	Tier 1 (gene analysis on EATS pathways}
8	CTC	Antimicrobial agent [*pharmaceutical*]	1. Enhancement of serum E2 and liver VTG content in male fish	Kim et al. (2007) Ji et al. (2010)	Tier 2 (thyroid-dependent mechanisms)
9	*p,p′*-DDE	DDT metabolite [*agricultural*]	1. Increased HSI	Zhang and Hu (2008)	Tier 2 (thyroid-dependent mechanisms)
			2. Development of intersex	Horie et al. (2022a)	
			3. Upregulation of *vtg1*, *vtg2*, *chgH*, *chgL*, and *esr1* genes in the liver of male fish		
10	*o,p′*-DDT	Organochlorine pesticide [*agricultural*]	1. Female-biased sex ratio in fish	Edmunds et al. (2000)	(Thyroid-dependent mechanisms)
			2. Ova–testis	Cheek et al. (2001)	
			3. *chgH*, *chgL*, *chgHm*, *vtg1*, *vtg2*, and *esr1* mRNAs induced in the liver of male	Kuhl and Brouwer (2006) Uchida et al. (2010)	
11	DES	Nonsteroidal estrogen [*pharmaceutical*]	1. *vtg1* mRNA was upregulated in males	Zeng et al. (2005)	Tier 2 (thyroid-dependent mechanisms)
			2. Sex-reversed males laid eggs	Lei et al. (2016)	
12	EDS	Organochlorine pesticide [*agricultural*]	1. Female-biased sex-ratio	Teather et al. (2005); Lee et al. (2013)	Tier 2 (thyroid-dependent mechanisms)
			2. Serum VTG induced in male fish		
13	EQ	Metabolite of the soy isoflavone daidzein [*natural product*]	1. Testis–ova formation in males	Kiparissis et al. (2003a)	Tier 2 (thyroid-dependent mechanisms)
			2. Intersex	Wang et al. (2016)	
			3.11-KT in the plasma reduced		
14	E3	Natural estrogen [*pharmaceutical*]	1. Testis–ova	Metcalfe et al. (2001)	Tier 2 (thyroid-dependent mechanisms)
			2. Induced *vtg1* mRNA in males	Zeng et al. (2005)	
15	E1	Natural estrogen [*pharmaceutical*]	1. Female-biased sex ratio	Metcalfe et al. (2001)	(Thyroid-dependent mechanisms)
			2. Liver VTG induced in both sexes	Nakamura et al. (2014a) Kawashima et al. (2022)	
16	4-MBC	Camphor derivative [*personal care product*]	1. Enhancement in the serum VTG in both sexes	Inui et al. (2003)	Tier 2 (thyroid-dependent mechanisms)
			2. Upregulation of *vtg1*, *vtg2*, *chgL*, and *chgH*, and *esr1* mRNAs in the liver of males	Liang et al. (2020)	
			3. Decrease in 11-KT in the plasma of males and enhancement of E2 in females		
			4. Inhibition of spermatogenesis in the testis		
17	MPB	Antimicrobial agent [*personal care product*]	1. Plasma VTG content increased in males	Yamomoto et al. (2011)	Tier 2 (thyroid-dependent mechanisms)
			2. Upregulation of *vtg2*, *chgL*, *chgH*, *chgHm*, and *esr1* in the liver of males	Kawashima et al. (2022)	
18	4-NP	Alkylphenol [industrial]	1. Males developed testis–ova with the sex ratio skewed toward female	Gray and Metcalfe, (1997); Nimrod and Benson, (1998); Shioda and Wakabayashi, (2000); Tabata et al. (2001); Yokota et al. (2001); Islinger et al. (2002); Kashiwada et al. (2002); Lee et al. (2002); Kang et al. (2003); Seki et al. (2003a); Nozaka et al. (2004); Kobayashi et al. (2005); Zeng et al. (2005), Balch and Metcalfe, (2006); Ishibashi et al. (2006); Jin et al. (2011a); Lee et al. (2012); Miyagawa et al. (2014); Tohyama et al. (2015); Watanabe et al. (2017); Ishibashi et al. (2020); Horie et al. (2021); Kawashima et al. (2022)	(Thyroid-dependent mechanisms)
			2. HSI in adult males increased		
			3. Serum VTG in males and hepatic VTG in both sexes increased		
			4. Female-like anal fins in some males		
19	OMC	Organic UV-B filter (PCP) [*personal care product*]	1. Enhancement of plasma VTG in males	Inui et al. (2003)	Tier 2 (thyroid-dependent mechanisms)
			2. Upregulation of *vtg1*, *vtg2*, *chgL, chgH*, and *esr1* mRNAs in males		
20	4-OP	De-ethoxylated alkylphenol [*industrial*]	1. Female-biased sex ratio	Knorr and Braunbeck (2002)	Tier 1 (thyroid-dependent mechanisms)
			2. Some F1 males developed testis–ova		
21	PCPL	Insecticide [*agricultural*]	1. Nonlinear enhancement in the plasma VTG levels in males and a concentration-dependent decrease in plasma VTG levels in females	Zha et al. (2006)	Tier 1 (thyroid-dependent mechanisms)
			2. Testis–ova formation in males and a degenerative ovary in females		
22	PPB	Personal care product [*personal care product*]	1. Enhancement in the plasma VTG content in males	Inui et al. (2003)	Tier 2 (interruption in swim bladder inflation needs further studies in thyroid-dependent mechanisms)
			2. Upregulation of *vtg1*, *vtg2*, *chgL*, *chgH*, and *esr1* in the liver of male fish	Gonzalez-Doncel et al. (2014a) Kawashima et al. (2022)	
23	4t-OP	Alkylphenol [*industrial*]	1. Sex ratio skewed toward females	Gray et al. (1999a)	Effect on swim bladder inflation needs further study on thyroid-dependent mechanisms
			2. Testis–ova observed in male fish	Gray et al. (1999b)	
			3. Liver VTG increased in both sexes	Gronen et al. (1999)	
			4. Inhibition of spermatogenesis	Seki et al. (2003a)	
			5. HSI in adult males increased	Nozaka et al. (2004)	
			6. Basophilia in the male liver	Flynn et al. (2017) Horie et al. (2022a) Kawashima et al. (2022)	
24	4t-PP	Alkylphenol [*industrial*]	1. The appearance of secondary sexual features was reduced in males	Seki et al. (2003b)	Tier 2 (thyroid-dependent mechanisms)
			2. Testis–ova in the gonad of males	Yokota et al. (2005)	
			3. Hepatic VTG enhanced in both sexes	Onishi et al., 2021	
			4. HSI increased in males	Kawashima et al. (2022)	
25	TBCO	Brominated flame retardant [*industrial*]	1. Upregulation of *chgHm* in the liver of males	Saunders et al. (2015)	Tier 2 (thyroid-dependent mechanisms)
			2. Upregulation of *chgH*, *vtg2*, and *esr1* in the liver of females	Sun et al. (2016c)	
			3. Downregulation of *esr1, esr2a,* and *arα* in both the testis and ovary	Devoy et al. (2023)	

**TABLE 4:** Potential EED antagonist chemicals identified by the literature search.

Serial number	Name of the chemical	Nature (source)	Significant endpoint	Reference	Recommendation
1	ATZ	Herbicide [*agricultural*]	1. *cyp19a* mRNA upregulated in the brain	Zhang et al. (2008d)	Tier 2 (thyroid-dependent mechanisms)
			2. Downregulation of *esr1* mRNA in the testis	Richter et al. (2016)	
			3. VTG in the liver of females reduced		
2	MET	Drug (*pharmaceutical*)	1. Intersex observed in females	Lee et al. (2019a)	Tier 2 (studies related to thyroid-related gene expression are necessary)
			2*. vtg1* declined in males		
			3. Thyroid histology remained unchanged		
3	PFOA	Fluorinated organic compounds (wastewater effluent)	1. Reduced fecundity	Lee et al. (2017a)	Tier 2 (studies related to thyroid-related gene expression are necessary)
			2. Increase in the serum VTG content in F2 males		
			3. Male-biased sex ratio with no change in intersex either in F1 or F2		
4	TPhP	Flame retardant/plasticizer [*industrial*]	4. Larval exposure reduced ovarian development in females	Li et al. (2019)	Tier 2 (thyroid-dependent mechanisms)
			5. Plasma T enhanced in females	Kawashima et al. (2022)	
			6. Hepatic VTG in females reduced		

**TABLE 5:** Potential androgen endocrine-disrupting agonist chemicals identified from the literature search.

Serial number	Name of the chemical	Nature (source)	Significant endpoint	Reference	Recommendation
1	11-OA	Glucocorticoid metabolite [*pharmaceutica*l]	1. Male-biased sex ratio	Grillitsch et al. (2010)	Tier 1 (gene expression analysis and thyroid-dependent mechanisms)
2	BF	Pyrethroid insecticide [*agricultural*]	1. Induced masculinization in the anal fin papillae	Bertotto et al. (2019)	Tier 2 (gene expression analysis related to EATS pathways and thyroid-dependent mechanisms)
			2. Male-biased sex ratio		
3	CFD	Antibiotic (*pharmaceutical*)	1. Plasma E2 was decreased in males and enhanced in females	Kim et al. (2017)	Tier 2 (thyroid-dependent mechanisms)
			2. Downregulation of *cyp19a* in the testis and upregulation of *cyp19*a in the ovary		
			3. Sex-specific alteration in the gene expression of the HPG axis		
4	DHT	Metabolite of testosterone [*pharmaceutical*]	1. Anal fin papillae increased in both sexes	Spirhanzlova et al. (2020); Onishi et al. (2021)	Tier 1 (gene expression analysis related to EATS pathways and thyroid-dependent mechanisms)
			2. Sex ratio skewed toward males		
5	GEN	Isoflavone [*natural Product*]	1. Masculinization features in the secondary sex characteristics of XX females	Hishida and Kawamoto (1970); Kiparissis et al. (2003a) Schiller et al. (2013, 2014)	Tier 2 (downregulation of *dio2* indicated more studies on thyroid-dependent mechanisms are necessary)
6	LNG	Second-generation progestin (*pharmaceutical*)	1. Liver VTG downregulated in females	Onishi et al. (2021); Pandelides et al. (2021); Watanabe et al. (2023)	Tier 2 (effects on the swim bladder suggest more studies on thyroid-dependent mechanisms are necessary)
			2. Masculinization of the anal fin papillae in females		
			3. Ovotestis in females		
7	MT	Synthetic androgen [*pharmaceutical*]	1. Sex reversal of XX females	Papoulias et al. (2000); Chikae et al. (2004); Nozaka et al. (2004); Seki et al. (2004); Kang et al. (2008); Ogino et al. (2014); Myosho et al. (2019); Onishi et al. (2021)	Thyroid-dependent mechanisms
			2. Serum VTG decreased in females		
			3. Upregulation of *gsdf* mRNA in XX fish		
8	P4	Female hormone (steroid) [*pharmaceutical*]	1. Females developed papillae on the anal fin rays	Onishi et al. (2021)	Tier 2 (thyroid-dependent mechanisms)
9	SPR	Synthetic aldosterone receptor agonist [*pharmaceutical*]	1. Anal fin papillae increased in both sexes	LaLone et al. (2013)	Tier 2 (thyroid-dependent mechanisms)
			2. Hepatic *vtg* reduced in female fish		
10	T	Male hormone (steroid) [*pharmaceutical*]	1. Intersex gonad	Koger et al. (2000)	Tier 1 (thyroid-dependent mechanisms)

**TABLE 6:** Potential AED antagonist chemicals identified by the literature search.

Serial number	Name of the chemical	Nature (source)	Significant endpoint	Reference	Recommendation
1	CPA	Male contraceptive [*pharmaceutical*]	1. Testis–ova observed in male fish	Kiparissis et al. (2003b)	Tier 2 (gene expression analysis of EATS pathways and thyroid-related mechanisms)
			2. No difference in the phenotypic sex ratio		
			3. Inhibition of spermatogenesis		
2	DZ	Organophosphate insecticide [*agricultural*]	1. Number of anal fin papillae in F1 male fish reduced	Hamm and Hinton, (2000); Flynn et al. (2018); Kawashima et al. (2022)	Tier 2 (effects on the swim bladder suggests more studies on thyroid-dependent mechanisms are necessary)
3	2-EHHB	Antimicrobial agent (*personal care product*)	1. Hepatic *vtg1* upregulated in F1 males and downregulated in F2 males	Matten et al. (2023)	Tier 2 (gene expression analysis of EATS pathways and thyroid-related mechanisms)
			2. Anal fin papillae in F2 males reduced		
			3. Delay in reproductive tract development in F1 males		
			4. Eosinophilia observed in renal ducts (kidney) of females		
4	FNT	Organophosphate pesticide [*agricultural*]	1. Number of papillary processes decreased in XY medaka	Horie et al. (2017; 2022a)	Tier 1 (thyroid-related mechanisms)
5	KC-400	Polychlorinated biphenyl (*industrial*)	1. Downregulation of *chgL*, *chgHm,* and *arα*, in both males and females	Nakayama et al. (2011)	Tier 1 (thyroid-related mechanisms)
			2. Downregulation of *vtg1* in males and upregulation in females		
6	LD-BP	Structural analog of bisphenol A [*industrial*]	1. Liver VTG in males and females increased	Li et al. (2016, 2017)	Tier 1 (thyroid-related mechanisms)
			2. Aggregation and hyperplasia of interstitial cells occurred in the testis, while atretic follicles, with interstitial cell fibrosis, occurred in the ovary		
7	PCB 126	Coplanar PCB (*persistent organic pollutants*)	1. Downregulation of *chgL*, *chgHm*, and *arα*, in both males and females	Nakayama et al. (2011)	Tier 1 (thyroid-related mechanisms)
			2. Downregulation of *vtg1* in males and upregulation of *vtg1* in females		
8	TCrP	Organophosphate flame retardant [*industrial*]	3. Suppression of 11-KT and T levels and enhanced E2 level in the plasma of male fish	Chen et al. (2022)	Tier 1 (thyroid-related mechanisms)
			4. Dilated the efferent duct of the testis		
			5. Intersex development		

**TABLE 7:** Potential TED agonist chemicals identified from the literature search.

Serial number	Name of the chemical	Nature (source)	Significant endpoint	Reference	Recommendation
1	MTC	Herbicide [*agricultural*]	1. Upregulation of the expression of *trα, trβ,* and *dio2* mRNAs in females	Jin et al. (2011b)	Tier 1 (thyroid histopathology and EATS-dependent mechanisms)

**TABLE 8:** Potential TED antagonist chemicals identified from the literature search.

Serial number	Name of the chemical	Nature (source)	Significant endpoint	Reference	Recommendation
1	ATBC	Non-phthalate plasticizer [*industrial*]	1. Disruption of swim bladder inflation	Horie et al. (2022b); Horie et al. (2023b)	Tier 1 (downregulation of *vtg1* and *vtg2* mRNAs in the liver of XX fish indicated more studies needed on EAS pathways are required)
			2. Downregulation of *trα*, *trβ*, and *dio2*		
2	DEHS	Plasticizer [*industrial*]	1. Downregulation of *dio2*	Horie et al. (2022c)	Tier 1 (more studies needed on EATS pathways)
3	DIC	NSAID [*pharmaceutical*]	1. Swim bladder inflation inhibition in larvae	Hong et al. (2007); Lee et al. (2011): Yokata et al. (2017), Yokata et al. (2018); Pandelides et al. (2021)	Tier 2 (more studies needed on EATS pathways)
4	EHMC	Organic ultraviolet UV-B filter [*personal care products*]	1. T3 and T4 concentrations decreased	Lee et al. (2019b)	Tier 2 (studies other than those based on EATS pathways are necessary)
			2. Downregulation of *dio2*		
			3. Upregulation of *trh*		
5	PFBA	Halogenated chemical [*industrial*]	1. No swim bladder inflation	Godfrey et al. (2019); Horie et al. (2022d)	Tier 1 (more studies needed on EAS pathways)
6	(PFOS/PFOSA)	Halogenated compound [*industrial*]	1. Hyperplasia, hypertrophy, and colloidal depletion in thyroid follicles	Ji et al. (2008)	Tier 1 (more studies needed on EATS pathways)
				Kang et al. (2019)	
7	PTU	Anti-thyroid medicine [*pharmaceutical*]	1. Modulation of swim bladder inflation	Horie et al. (2023a)	Tier 1 (studies related to EAS pathways)
8	SPC	Anti-thyroid chemical [*industrial*]	1. Downregulation of *trα* and *trβ* genes	Lee et al. (2014)	Tier 1 (studies related to EAS pathways)
			2. Upregulation of *dio2*		
			3. Decrease in T4 levels but T3 remained unaltered		
			4. Fecundity decreased with the increase in temperature		
9	TU	Anti-thyroid chemical [*industrial*]	5. Decreased thyroid hormone levels in adult fish and fertilized eggs	Tagawa and Hirano (1991)	Tier 1 (although anti-thyroid effects were established, EAS-mediated pathways need to be investigated)
			6. No effect on the length and weight of the larvae		
10	RND	Herbicide (commercial formulation of glyphosate) [*agricultural*]	1. Uninflated swim bladder	Smith et al. (2019)	Tier 1 (studies related to thyroid histophysiology and thyroid-dependent gene expression)
11	TDCPP	Halogen-containing organophosphorus compound [*industrial*]	1. Females failed to inflate the swim bladder	Godfrey et al. (2019)	Tier 1 (upregulation of *vtg1* and *vrg2* mRNAs indicates further studies on EAS mechanisms are necessary)
				Horie et al. (2022a); Horie et al. (2022d)	

**TABLE 9:** Potential steroidogenesis stimulating EDCs identified from the literature search.

Serial number	Name of the chemical	Nature (source)	Significant endpoint	Reference	Recommendation
1	OCL	Organic UV filter (PCP) [*personal care product*]	1. Upregulation of *fshβ*, *lhβ, fshr*, *lhr, ar*, *esr1*, *esr2a*, StAR, *hsd3β*, *cyp17α,* and *cyp19β* mRNAs in the HPG axis	Yan et al. (2020)	Tier 2 (thyroid-dependent mechanisms)
			2. E2 and 11-KT increased in plasma		
			3. Upregulation of *vtg* in the liver of males and females		
2	FPN	Phenylpyrazole insecticide [*agricultural*]	1. Upregulation of *StAR, cyp17a*, and *cyp19b* in males	Sun et al. (2014)	Tier 2 (thyroid-dependent studies)
			2. Upregulation of both *vtg1* and *vtg2* mRNAs in both sexes	Wagner et al. (2017)	
			3. No alteration occurred in *esr1*, *esr2a*, and *arα* in both sexes		
3	RCT	β-adrenergic agonist drug [*pharmaceutical*]	4. Upregulation of *cyp19a* and *cyp19b* mRNAs in females	Sun et al. (2016a)	Tier 2 (thyroid-dependent mechanisms)
			5. Upregulation of *vtg1*, *vtg2*, *esr1*, and *esr2* mRNAs in females		
4	TRI	Pharmaceuticals [*pharmaceutical*]	1. Upregulation of *StAR*, *3β-hsd*, *20β-hsd*, *cyp11a*, *cyp11b*, *cyp17a*, *cyp17b*, and *cyp19a* in males	Sun et al. (2014)	Tier 2 (thyroid-dependent mechanisms)
			2. Upregulation of *vtg1* and *vtg2* in males and downregulation of *vtg1* and *vtg2* in females		
			3. Upregulation of *esr1* and *arα* in males		

**TABLE 10:** Potential steroidogenesis inhibitory EDCs identified by the literature search.

Serial number	Name of the chemical	Nature (source)	Significant endpoint	Reference	Recommendation
1	BP	UV filters used in cosmetics [*personal care product*]	1. Liver VTG in both male and females increased by BP2	Coronado et al. (2008)	Tier 2 (thyroid-dependent mechanisms)
			2. Enhanced T concentration in the serum of male fish by BP3	Kim et al. (2014); Kawashima et al. (2022)	
			3. Upregulation of *vtg1* and *vtg2* mRNAs, and the VTG protein in the liver of male fish by BP3		
			4. Downregulation of gonadal *StAR*, *cyp17*, *hsd3b*, *hsd17b3*, and *cyp19a* by BP3		
2	FAD	Nonsteroidal aromatase inhibitor [*pharmaceutical*]	1. Aromatase enzyme activity reduced	Suzuki et al. (2004)	Tier 2 (thyroid-dependent mechanisms)
			2. Upregulation of *cyp19a* in the ovary	Kuhl and Brouwer, (2006)	
			3. Downregulation of *esr1* and *chgL* in the liver of females	Thresher et al. (2011) Park et al. (2008) Zhang et al. (2008b)	
3	LET	Nonsteroidal triazole [*pharmaceutical*]	1. Male-biased sex ratio	Sun et al. (2007b, 2009, 2011a)	Tier 2 (thyroid-dependent mechanisms)
			2. Downregulation of *esr1, vtg1,* and *vtg2* in the liver of males	Liao et al. (2014)	
			3. Serum VTG levels remained unaltered in males and decreased in females		
			4. Upregulation of *StAR*, *cyp11a*, *cyp11b*, *cyp17a*, *cyp17b*, and *esr2* and downregulation of cyp19 b and arα in the ovary		
			5. Upregulation of *cyp11a* and *cyp11b* and no alteration in *cyp17a, cyp17b, cyp19a,* and *cyp19b* mRNA*s* in the testis		
4	LNR	Herbicide [*agricultural*]	1. Downregulation of *3β-hsd* and *cyp11b*	Schiller et al. (2014)	Tier 1 (gene expression analysis related to EATS pathways and thyroid-dependent mechanisms)
			2. E2 or T-induced expression of *chgH* was downregulated	Spirhanzlova et al. (2017)	
5	PRN	Herbicide [*agricultural*]	1. Downregulation of *cyp11b*, *3β-hsd*, *gnrhr2*, and *cyp19a1b*	Schiller et al. (2014)	Tier 1 (gene expression analysis related to EATS pathways and thyroid-dependent mechanisms)
6	TPT-Cl	Organotin compound [*industrial*]	1. Downregulation of *17β-hsd1* and *cyp19a* in the ovary	Zhang et al. (2008e)	Tier 1 (studies on EATS pathways and thyroid-dependent mechanisms)
			2. Upregulation of *cyp1a* and *cyp2a1*	Horie et al. (2022a)	
			3*. ugt2a3* and *17β-hsd1* in the liver of both sexes		
			4. No change in *gsdf* mRNA expression in both XX and XY embryos		

**TABLE 11:** Potential EDCs with unidentified EATS pathways.

Serial number	Name of the chemical	Nature (source)	Reference	Reason
1	ACT	NSAID (*pharmaceutical*)	Kim et al. (2012)	Limited data (nonlinear induction of hepatic VTG in males was due to stress)
2	AMT	Herbicide [*agricultural*]	Horie et al. (2022a)	Insufficient data
3	BZT-UV	UV stabilizer; persistent organic pollutants (POPs) [*personal care product*]	Fujita et al. (2022)	Due to stress
4	BKC	Quaternary ammonium compound [personal care product]	Kim et al. (2020)	Insufficient data (enhancement of *vtg1* in the whole body was probably due to stress)
5	i-BP	Antimicrobial [personal care product)	Yamamoto et al. (2007)	Insufficient data (estrogenic potential)
6	n-BP	Antimicrobial [personal care product)	Yamamoto et al. (2007)	Insufficient data (estrogenic potential)
7	Cd	Metal [*inorganic*]	Tilton et al. (2003), Hirako et al. (2017)	Insufficient data (anti-androgenic effects were probably mediated through stress)
8	ClxBPA	Chlorinated product of BPA	Tabata et al. (2004)	Limited data (the compound showed estrogenic potential with regard to serum VTG in male fish)
9	CMP	Biocide [*personal care product*]	Flynn et al. (2017); Onishi et al. (2021)	Inconsistent alteration of liver VTG in both sexes indicate the estrogenic potential of the compound
10	CYN	Herbicide [*agricultural*]	Kawashima et al. (2022)	Effects are not mediated through EATS pathways
11	CHDM	Plasticizer [*industrial*]	Jang and Ji, (2015)	Effects are not mediated through EATS pathways
12	DBP	Plasticizer [*industrial*]	Nozaka et al. (2004)	VTG in male fish remained unchanged
13	DEHP	Plasticizer [*industrial*]	Metcalfe et al. (2001)	Effects are not mediated through EATS pathways
14	DIBP	Plasticizer [*industrial*]	Kawashima et al. (2022)	Limited AED features (hepatic VTG reduced in females)
15	END	Organochlorine pesticide [*agricultural*]	Horie et al. (2022a)	Limited information (not related to EATS-mediated pathways)
16	FNC	Insecticide [*agricultural*]	Spirhanzlova et al. (2017)	Effects not related to EATS pathways
17	FV	Pyrethroid insecticide [*agricultural*]	Kawashima et al. (2022)	No effects on estrogen-dependent mechanisms
18	FLX	Antidepressant [*pharmaceutical*]	Foran et al. (2004)	Mostly due to toxicity and not mediated through EATS pathways
19	FLR	Herbicide (*Agricultural*)	Jin et al. (2020)	Effects are mediated through oxidative stress
20	GLP	Herbicide [*agricultural*]	Smith et al. (2019)	Effects are mediated through oxidative stress
21	GO	Nanocarbon [*inorganic*]	Dasmahapatra et al. (2020a, b)	Effects are not mediated through EATS pathways
			Myla et al. (2021a); Myla et al. (2021b)	
			Asala et al. (2021); Asala et al. (2022)	
			Dasmahapatra and Tchounwou (2022a); Dasmahapatra and Tchounwou (2022b); Dasmahapatra and Tchounwou (2023a); Dasmahapatra and Tchounwou (2023b)	
22	IBP	Nonsteroidal anti-inflammatory drug [*pharmaceutical*]	Flippin et al. (2007); Han et al. (2010)	VTG induction in male fish serum is probably due to stress
23	LIN	Antibiotic (*pharmaceutical*)	Kim et al. (2012)	Insufficient data (insignificant increase in hepatic VTG in male fish)
24	MTZ	Goitrogen [*pharmaceutical*]	Godfrey et al. (2019)	Insufficient data (vtg gene expression upregulated in males)
25	MXC	Organochlorine pesticide [*agricultural*]	Nimrod and Benson (1998)	Insufficient data
			Zeng et al. (2005)	
26	MCB	Fungicide [*agricultural*]	Lin et al. (2014)	Induced cyp3a enzyme activities
27	1NT	Insecticide [*agricultural*]	Kawashima et al. (2022)	Limited data (hepatic VTG enhanced in females)
28	NPX	NSAID [*pharmaceutical*]	Kwak et al. (2018)	Although transcription of *vtg1, erβ2,* and *cyp17* genes significantly increased, data are still limited for consideration as EEDs
29	NDEA	Carcinogen [*industrial*]	Nair et al. (2017)	Limited data (sex-specific reduction in germ cells occurred only in the ovary)
30	OYZ	Herbicide [*agricultural*]	Hall et al. (2005, 2007)	Insufficient data (induction of choriogenin in liver and abnormal gonad histology)
31	OXF	Herbicide [*agricultural*]	Powe et al. (2018)	Toxicological effects
32	OTC	Antibiotic [*pharmaceutical*]	Ji et al. (2010, 2012)	Insufficient data
33	PDM	Herbicide [*agricultural*]	Kawashima et al. (2022)	Insufficient data (hepatic VTG enhanced in males)
34	PHN	Aromatic hydrocarbon [burning of fuels]	Horng et al. (2010)	No significant EATS-mediated effects
35	PHT	Epileptic drug [*pharmaceutical*]	Kawashima et al. (2022)	Insufficient data
36	RLX	SERM [*pharmaceutical*]	Onishi et al. (2021)	Insufficient data (liver VTG enhanced in males and reduced in females)
37	SFT	Veterinary pharmaceutical [*pharmaceutical*]	Ji et al. (2010)	Limited data (enhancement of the serum E2 level in male fish)
38	SRF	Herbicide [*agricultural*]	Hall et al. (2005)	Limited data (only *chg* in males enhanced)
39	BDE-47	Flame retardants [*industrial*]	Gonzalez-Doncel et al. (2014b); Gonzalez-Doncel et al. (2016)	Lack of ED effects related to EATS pathways
			Gonzalez-Doncel et al. (2017)	
			Beltran et al. (2022)	
40	TRA	Metabolite of TRB (*agricultural*)	Robinson et al. (2017)	Lack of adverse effects on fecundity
41	TRF	Fungicide (*agricultural*)	Lin et al. (2014)	Induced cyp1a and cyp3a activities in the liver
42	TRD	Fungicide [*agricultural*]	Lin et al. (2014); Chu et al. (2016); Liu et al. (2018)	Limited information (upregulation of *vtg2* and *cyp3a40* and downregulation of *cyp3a38*, *vtg1*, *esr1*, and c*yp1a* in the liver of females)
43	TBT	Biocide [*agricultural*]	Nozaka et al. (2004)	Limited data (inhibition of brain aromatase)
			Kuhl and Brouwer, (2006)	
			Hano et al. (2007)	
			Zhang et al. (2008d)	
			Horie et al. (2018, 2022a)	
44	TCS	Antimicrobial [*industrial*]	Foran et al. (2000)	Inconsistent data (hepatic VTG increased in males)
			Ishibashi et al. (2004)	
			Mihaich et al. (2019)	
			Song et al. (2020)	
			Kawashima et al. (2022)	
45	Nano zinc oxide (nZnO)/zinc sulfate (ZnSO_4_)	Metal [*inorganic*]	Paul et al. (2021)	Toxic effects (reduced follicular growth and maturation in the ovary)

### 3.1 EEDs

For the identification and classification of EEDs from the searched chemicals, we considered three chemicals as references, E2 and EE2 as agonists, and TAM as antagonists (Table 1). Based on these reference chemicals, several endpoints, such as the female-biased sex ratio, induction of serum VTG (protein) in male fish, alteration of the secondary sex characteristics (anal fin papillae in the male fish), and up- or downregulation of *vtg* and *chg* genes/mRNAs in the liver of male fish, as well as the estrogen receptors (ERs) of the HPG axis in both sexes, were considered (Table 1). Using these strategies, we reviewed 108 articles, which is 52.68% of the searched articles, consisting of 25 chemicals as agonists and 4 chemicals as antagonists (Tables 3, 4). Adding three reference chemicals to the list, the number of EED agonists increased to 27 (21.09% of 128 chemicals) and antagonists to 5 (3.9% of 128 chemicals), altogether 32, which is 25% of the total (128 chemicals) chemicals searched by the literature survey. Alternatively, it appears that for every 100 EDCs, ∼21 of them are identified as EED agonists and ∼4 of them are identified as EED antagonists. Moreover, considering the 108 articles that studied EEDs, every EED chemical was studied in 3.375 articles (27 articles: 8 EEDs). Moreover, among EED agonists, other than two reference chemicals (E2 was reviewed in 37 articles and EE2 in 27 articles), 4-nonylphenol (4-NP; 23 articles), bisphenol A (BPA; 21 articles), and 4-*tert*-octylphenol (4-t-OP; 8 articles) are the most studied EED agonist chemicals in Japanese medaka (Table 3). Among others, *o,p*′-DDT (4 articles), 4t-PP (4 articles), E1 (3 articles), PPB (3 articles), and TBCO (3 articles) have drawn significant interest among investigators. The remaining 17 estrogen agonists were studied either twice (8 chemicals) or once (9 chemicals). For EED antagonists, the reference chemical TAM was studied in five articles, whereas ATZ was studied twice (Ritcher et al., 2016), MET once (Lee et al., 2019), and TPhP in two articles (Li et al., 2019; Kawashima et al., 2022). Moreover, 16 of the EEDs as agonists and 4 as antagonists were recommended for Tier 2 tests. Therefore, based on the literature search, we recommend that eight chemicals (E1, E2, EE2, BPA, *o,p*′-DDT, 4-NP, 4-t-OP, and TAM) showed enough potential to be considered EEDs in Japanese medaka and did not require any further Tier 2 tests for estrogen signaling mechanisms. Furthermore, except PPB (Gonzalez-Doncel et al., 2014a), 4t-OP (Gray et al., 1999b), and MET (Lee et al., 2019), in most of the EED chemicals, whether agonists or antagonists, the thyroid-related endpoints remained uninvestigated, even though the reference agonists (E2 and EE2) have the potential to inhibit swim bladder inflation (a thyroid-related endpoint) in a concentration-dependent manner in larvae if the embryos were exposed either to E2 or EE2 during development (Pandelides et al., 2021).

### 3.2 AEDs

For AEDs, four chemicals, 11-KT and TRB as agonists and FLU and TRB as antagonists, were considered references (Table 1). Based on these reference chemicals, the apical endpoints, such as masculinization of females (development of anal fin papillae), male-biased sex ratio, upregulation of *gsdf* mRNA in XX embryos, ovotestis, and downregulation of *vtg1*, *vtg2*, *chgH*, and *chgHm* gene transcripts in the liver of both male and female fish (Table 1), were mostly considered during the evaluation of AEDs. With these efforts, from 46 articles, which is 22.43% of the sorted articles (Table 2), we identified 10 chemicals as agonists (Table 5) and 8 chemicals as antagonists (Table 6). With the addition of four reference chemicals, the number of AEDs increased to 22 (∼9% agonists and ∼8% antagonists), which is 17.18% of the 128 chemicals screened through the literature search. Alternatively, for every 100 EDCs, ∼9 chemicals are identified as AED agonists and ∼8 chemicals are identified as AED antagonists. Moreover, with regard to 46 articles that studied 22 AEDs, it appears that one AED chemical was studied in 2.09 articles (approximately 2 articles:1 AED). Moreover, among the reference chemicals, effects of TRB were observed in 14 articles, FLU was in 8 articles, and 11-KT and VIN were included in 5 articles (Table 1). Other than the references, the ED effects of three compounds, DHT, LNG, and P4, were evaluated together (Onishi et al., 2021). Furthermore, among the androgen agonists, the AED effects of MT were peer-reviewed in eight articles, followed by GEN (four articles) (Table 4). Among the other agonists, LNG was reviewed in three articles, and the remaining seven chemicals were studied only once (Table 5). Among the apical endpoints, masculinization was induced by BF, GEN, LNG, P4, and SPR, while downregulation of hepatic *vtg* in females was observed in MT and SPR (Table 5). Among the eight chemicals identified as potential antagonists, the most studied chemical was DZ, which was studied in three articles (Hamm and Hinton, 2000; Flynn et al., 2018; Kawashima et al., 2022), followed by LD-BP and FNT, which were studied in two articles each (Li et al., 2016; 2017; Horie et al., 2017; 2022a). Other than these chemicals, the remaining five chemicals were studied once (one article/chemical). Moreover, based on the targeted apical endpoints related to AED and the literature review, we recommend that nine chemicals showed enough potential to proceed to Tier 2 tests, and five chemicals (FLU, 11-KT, MT, TRB, and VIN) did not require Tier 2 tests for the evaluation of androgen signaling mechanisms. In addition, similar to EEDs, the thyroid-related apical endpoints, such as hypertrophy of thyroid follicular cells, were induced by 11-KT (reference agonist) and FLU (reference antagonist) in Japanese medaka (Leon et al., 2007). Other than the references, LNG (agonist) and DZ (antagonist) showed the potential to modulate swim bladder inflation in Japanese medaka larvae during development (Hamm and Hinton, 2000; Pandelides et al., 2021). Furthermore, GEN (agonist) shows potential to regulate the expression of *dio2* mRNAs in larvae if the embryos were exposed to GEN during development (Schiller et al., 2013; 2014). Therefore, during the classification of EDCs as AED, the thyroid-related apical endpoints should not be ignored.

### 3.3 TEDs

For TEDs, three chemicals, T3 as the agonist and PFOA and TBBPA as antagonists, were considered references (Table 1). The apical endpoints, such as swim bladder inflation in larvae, disruption of thyroid histopathology, and up- or downregulation of TH receptor genes (*trα* and *trβ*) and deiodinases (*dio1* and *dio2*), were considered during TED evaluations. Our literature search found only 19 articles, which is 9.26% of the total articles (205 articles) sorted are focused on TED. From these articles, 12 chemicals, one as agonist (Table 7), and 11 chemicals as antagonists, were identified as TEDs (Table 8). Considering three references, 15 chemicals, 2 as agonists (1.56% of 128 EDCs) and 13 as antagonists (10.16% of 128 EDCs), which is only 11.72% of the screened chemicals (128 chemicals), showed TED effects on Japanese medaka. Alternatively, for 100 EDCs, 1.56 chemicals are identified as TED agonists, and ∼10 chemicals are identified as TED antagonists. Moreover, 19 articles identified 15 chemicals, which indicated that one TED was reviewed in 1.266 articles (approximately 5 articles:4 chemicals). Moreover, 5 chemicals, including three references and two antagonists (DIC and EHMC), were recommended to proceed to Tier 2 tests. The reference agonist T3 was studied in two articles, and the reference antagonists PFOA and TBBPA were included in four articles and 1 article, respectively (Table 1). Other than the references, the most studied chemical as a TED antagonist in Japanese medaka was DIC, which was peer-reviewed in five articles (Hong et al., 2007; Lee et al., 2011; Yokota et al., 2017; 2018; Pandelides et al., 2021). Other chemicals, such as ATBC, PFBA, PFOS/PFOSA, and TDCPP, were studied in two articles each. The remaining four antagonists were studied only once (Table 8). Although ATBC and TDCPP were evaluated as TED antagonists, the downregulation of liver *vtg1* and *vtg2* genes in XX fish by ATBC (Horie et al., 2023b) and upregulation of *vtg* mRNA in both male and female larvae by TDCPP (Godfrey et al., 2019) indicated that TED chemicals have the potential to regulate EAS pathways, which need further verifications.

### 3.4 MOS

For identification of the MOS chemicals in Japanese medaka, four chemicals, TPA and TRF as stimulators and KTC and PCZ as inhibitors, were used as reference chemicals (Table 1). The apical endpoints selected for steroidogenesis are either the up- or downregulation of *cyp19* genes that show potential to regulate the aromatase enzyme activity and lead to an increase or decrease in the circulating estrogen level in Japanese medaka. Our literature search selected 26 articles, which is 12.68% of the sorted articles, for the evaluation of steroidogenesis in Japanese medaka (Table 2). After reviewing these literature reports, four chemicals were considered stimulators of steroidogenesis and six chemicals were considered inhibitors (Table 9). Including the references, the total number of chemicals that interrupt steroidogenesis is 14, 6 stimulators (∼5%), and 8 inhibitors (∼6%), which is 10.93% of the identified chemicals that showed potential ED activities in Japanese medaka. Alternatively, for every 100 EDCs, 5 chemicals show potential to stimulate steroidogenesis and 8 chemicals inhibit steroidogenesis. Moreover, 14 MOS were identified after reviewing 26 articles, which indicated that for the identification of a chemical as MOS, 1.857 articles/MOS are reviewed (approximately 9 articles: 5 chemicals). Moreover, although the thyroid-related endpoints were not considered in these chemicals, including two references (TPA and TRF as agonists), nine chemicals (six as agonists and three as antagonists) were recommended for Tier 2 tests (Tables 9, 10). Among the stimulators, the ED activities of FPN, an insecticide, and TRI, a pharmaceutical product, were studied together (Sun et al., 2014). However, FPN was included separately in two articles (Sun et al., 2014; Wagner et al., 2017); the remaining three chemicals, OCL, RCT, and TRI, were investigated once (Sun et al., 2014; 2016a; Yan et al., 2020) (Table 9). Among inhibitors, the most studied chemical is FAD, a nonsteroidal aromatase inhibitor, which was studied in five articles (Suzuki et al., 2004; Kuhl and Brower, 2006; Zhang et al., 2008b; Park et al., 2008; Thresher et al., 2011). Moreover, LET, a nonsteroidal triazole, was included in four articles (Sun et al., 2007b; 2009; 2011a; Liao et al., 2014). BP, a UV filter used in cosmetics, was evaluated in three articles (Coronado et al., 2008; Kim et al., 2014; Kawashima et al., 2022), while the herbicide LNR and the organotin compound TPT-Cl were studied in two articles each (Table 10), and PRN was studied only once (Schiller et al., 2014). Although the apical endpoints of MOS are mainly concentrated on aromatase enzyme genes and enzyme activities, the ED effects of these compounds on Japanese medaka either as an EED or AED can also be observed in TRI (Yan et al., 2020), RCT (Sun et al., 2016a), BP (Coronado et al., 2008; Kawashima et al., 2022), FAD (Zhang et al., 2008b), and LET (Sun et al., 2007b).

### 3.5 Unclassified

Due to limitations in the selection of apical endpoints, we were unable to identify the targeted EATS pathways of 45 chemicals (35.15% of the EDCs) identified from 60 (29.26% of the articles sorted) articles (Table 11). Alternatively, among 100 EDCs, 35 chemicals remained unclassified within the EATS modalities due to the lack of sufficient information (Table 11). Moreover, 45 unidentified EDCs in 60 sorted articles indicated that one chemical remained unidentified in 1.33 articles reviewed (4 articles:3 chemicals). Among these chemicals, the ED potential of GO was described in the maximum number of articles (10 articles) targeting the gonads, thyroid, interrenal glands, and endocrine pancreas of Japanese medaka (Dasmahapatra et al., 2020a; Dasmahapatra et al., 2020b; Myla et al., 2021; Asala et al., 2021; Myla et al., 2021; Dasmahapatra and Tchounwou, 2022a; Asala et al., 2022; Dasmahapatra and Tchounwou, 2022b; Dasmahapatra and Tchounwou, 2023a; Dasmahapatra and Tchounwou, 2023b). Moreover, TBT, a biocide used in agriculture, has been studied in six articles and showed the potential to inhibit brain aromatase in Japanese medaka (Nozaka et al., 2004; Khul and Brouwer, 2006; Hano et al., 2007; Zhang et al., 2008; Horie et al., 2018; Horie et al., 2022a). Furthermore, TCS, an antimicrobial product, was peer-reviewed in five articles that showed potential to enhance hepatic VTG in male fish (Foran et al., 2000; Ishibashi et al., 2004; Mihaich et al., 2019; Song et al., 2020; Kawashima et al., 2022). In addition, the flame retardant 2,2′,4,4′-BDE47 was peer-reviewed in four articles, although it was unable to target any of the EATS-related pathways in Japanese medaka (Gonzalez-Doncel et al., 2014b; 2016; 2017; Beltran et al., 2022). Among others, ACT, BKC, ClxBPA, CMP, IBP, LIN, MET, PDM, RLX, SFT, and TCS, although studied in a limited number of articles (except TCS, in most cases one or two articles), showed estrogenic potential by inducing the serum or liver VTG content in male fish (Foran et al., 2000; Ishibashi et al., 2004; Kim et al., 2012; Flynn et al., 2017; Godfrey et al., 2019; Mihaich et al., 2019; Kim et al., 2020; Song et al., 2020; Onishi et al., 2021; Kawashima et al., 2022). Furthermore, NPX, a NSAID, showed estrogenic potential by upregulating the expression of *vtg1, erβ*, and *cyp17* genes in Japanese medaka (Kwak et al., 2018). Moreover, the potential ED effects produced by the rest of the chemicals (Table 11) are either due to induction of stress or mediated through pathways other than EATS.

## 4 Discussion

Japanese medaka (*Oryzias latipes*) is one of the small laboratory fish models used for the evaluation of EDCs found in the environment (OECD, 2018). Like all other vertebrates, EATS pathways and their associated hypothalamus pituitary-releasing and -stimulating hormones are targeted by EDCs and disrupt the normal development and reproductive processes of this fish. For the identification of EDCs that specifically affect the endocrine systems of Japanese medaka (*O. latipes*), we searched the research articles in PubMed (http://www.ncbi.nlm.nih.gov/pubmed) and Google Scholar (https://scholar.google.com/) databases with the search terms, Japanese medaka, *O. latipes*, and endocrine disruptions. We hypothesized that literature search and evaluation can identify the number and sources of EDCs that disrupted the EATS-related pathways of Japanese medaka (*Oryzias l*ati*pes*) and provide additional evidence for the selection of a chemical as to whether to proceed to Tier 2 tests or not.

We sorted 205 articles that involved 128 chemicals for review (Figures 1, 2; Tables 1–11). Due to wide variations in experimental protocols and methodologies described in the research articles (n = 205), especially in non-TG studies, interpretation of the data from the literature survey became more complex. Moreover, the use of different life stages (embryos/larvae/adults), diversity in the modes of exposure (injection, immersion, and feeding), or in the duration of exposure (restricted either only in one generation or continued through multiple generations) made the problem even more complex. Therefore, to maintain consistency in the apical endpoints associated with ED effects, among the 128 identified chemicals, we selected 14 chemicals as the reference (Table 1). These chemicals (references) are either evaluated in this model (Japanese medaka) as reference chemicals by other investigators or screened through Tier 2 tests, following OECD guidelines (Flynn et al., 2017; Onishi et al., 2021; Kawashima et al., 2022; Myosho et al., 2022). Among these chemicals, E2 and EE2 (estrogen agonists), TAM (estrogen antagonist), 11-KT and TRB (androgen agonists), FLU and VIN (androgen antagonists), and KTC and PCZ (steroidogenesis inhibitors) were verified as agonists or antagonists for *esr1* (for estrogen) and *arβ* (androgen) genes of Japanese medaka *in vitro* by RGA (Onishi et al., 2021; Kawashima et al., 2022). Additionally, the potential of E2, TAM, TRB, VIN, KTC, and PCZ as an EDC was evaluated in medaka through Tier 2 tests, following the MEOGRT protocol (Flynn et al., 2017). For stimulators of steroidogenesis, we considered TPA and TRF as reference chemicals (Jang and Ji, 2015; Zhu et al., 2015). For the thyroid, T3 as the agonist and PFOA and TBBPA as antagonists were considered, which were recently referenced by Godfrey et al. (2019) and Horie et al. (2023a) in Japanese medaka. Therefore, we think that the selection of reference chemicals for the identification of EATS-related apical endpoints and to set up guidelines is very reasonable and acceptable. Our approach identified 69 chemicals that show potential to target the EATS pathways of Japanese medaka, and 45 chemicals remained unclassified due to limited information, even though these unclassified chemicals induced ED-like effects in Japanese medaka (Table 11). Taken together, considering 14 references, 83 (69 identified +14 references = 83) chemicals are identified as EDCs (∼65%) that disrupt EATS pathways of Japanese medaka (*O. latipes*), and 45 EDCs (∼35%) remain unclassified due to the lack of sufficient information.

We further classified the EATS chemicals as agonists/stimulators and antagonists/inhibitors of EEDs, AEDs, and TEDs, and MOS. The apical endpoints selected for agonists should be in contrast with antagonists, and in many cases, these borderlines cannot be maintained. For example, one of the significant apical endpoints of an EED as an agonist is the upregulation of VTG in the liver of male (XY) medaka (Flynn et al., 2017); however, TAM, which was used as a reference chemical of the EED antagonist, increased the liver VTG content in male fish (Flynn et al., 2017). To avoid complicacy, during analysis, we ignored the classification of EATS chemicals as agonists and antagonists, and simply included all the agonists and antagonists together and expressed them as EEDs, AEDs, TEDs, and MOS where applicable (Table 2).

As mentioned previously, 128 EDCs were identified after reviewing 205 individual articles, which indicates that for the identification of a chemical as an EDC in Japanese medaka, more than one article was reviewed (1.60 articles/chemical, or the approximate ratio is 8 chemicals: 13 articles). Our studies also showed that after reviewing 165 articles, 83 EDCs were identified that targeted EATS pathways (Tables 3–10), and 45 chemicals remained unidentified after reviewing 60 articles (Table 11). Accordingly, approximately 65% of the EDCs were identified with their specific EATS targets after reviewing 80% of the searched articles and 35% of the EDCs remained unclassified after reviewing 20% of the searched articles (Table 2). Therefore, it appears that the databases consist of more articles as classified EDCs (related to EATS) than unclassified EDCs (Table 2). Moreover, as the EATS pathways are interdependent on each other through the common hypothalamus–pituitary axis (HP axis), it is very difficult to classify the EDCs on the basis of apical endpoints specific to the EATS pathways. However, our studies showed that more than 65% of the articles identified EDCs as EED, 28% of the articles identified EDCs as AED, 12% of the articles identified EDCs as TED, and 16% of the articles identified EDCs as MOS (Table 2), which can be arranged in the order of TED < MOS < AED < EED. Furthermore, among 83 EDCs that targeted EATS pathways, 39% of them are identified as EEDs, 27% are AEDs, 18% are TEDs, and 17% are MOS (Tables 3–10), and the order of arrangement appears to be MOS < TED < AED < EED. Therefore, the potential of literature searching to identify EATS-targeted chemicals in Japanese medaka partially supports the concept that the more the number of articles in the databases, the more the number of EDCs should be identified.

As recommended by USEPA, the effects of an EDC should be evaluated using a tier-based approach. In Tier-1 studies, the endpoints are focused mainly on lethal concentrations (LC/LD/IC_50_, NOEC, and LOEC), reproductive activity (fecundity, fertility, breeding behavior, and hatching of the embryos), sex reversal, secondary sexual features (the number of papillae in the anal fin rays which are present in juvenile/adult males and absent in female Japanese medaka), VTG (the egg yolk precursor protein), and choriogenins (the eggshell protein), which are absent in the liver of male fish, and histopathology of the gonad, liver, and kidney. The Tier 2 approach is multigenerational, consisting mostly of the same features evaluated in Tier 1 (fecundity, fertility, hatching, VTG content of the male fish liver, secondary sexual features, sex reversal, survivability of embryos, larvae, and adults, and histopathology of the gonad, liver, and kidney). Even though the Tier 2 tests are time-consuming, expensive, and need proper validation of the chemicals as an EDC through Tier 1 screening, for proper classification of the EDCs and their respective target endocrine organs or hormones in fish (Japanese medaka), multigenerational studies (Tier 2) are necessary (Kawashima et al., 2022). Accordingly, among 83 EATS (69 classified and 14 references), we recommend that six of the references (11-KT, T3, PFOA, TBBPA, TPA, and TRF), due to the limited number of articles (studies in Japanese medaka), should be considered high-priority candidate substances for Tier 2 testing. The eight other references (E2, EE2, TAM, TRB, FLU, VIN, KTC, and PCZ) were already verified either as reference chemicals during the evaluation of other EDCs or through multigenerational MEOGRT tests (OECD TG 240) (Flynn et al., 2017; Onishi et al., 2021; Kawashima et al., 2022). Therefore, these eight reference chemicals did not need any further Tier 2 tests for potential EAS-related effects; however, evaluation of thyroid-dependent mechanisms of these chemicals may require investigation (Myosho et al., 2019, 2021; Pandelides et al., 2021).

During screening of EEDs, among the identified chemicals, we recommend 16 (AR-1260, BZP, BMT, CFR, CTC, *p,p*′-DDE, DES, EDS, EQ, E3, 4-MBC, MPB, OMC, PPB, 4t-PP, and TBCO) as agonists, and 4 chemicals (ATZ, MET, PFAA, and TPhP) as antagonists were high-priority chemicals for Tier 2 tests. Among the rest, EED potentials of *o,p′*-DDT and 4-t-OP were evaluated by multigenerational MEOGRT tests (Flynn et al., 2017) and probably did not require any further Tier 2 tests as well (Flynn et al., 2017). In addition, BPA, E1, 4-NP, and 4-t-OP were recommended for Tier 2 tests after successful evaluation through the OECD TG 229 protocol (Kawashima et al., 2022). Moreover, our literature search found that BPA was reviewed in 21 articles, 4-NP in 23 articles, 4-t-OP in 8 articles, and E1 in 3 articles (Table 3). Therefore, we believe that these EEDs (E1, BPA, *o,p′-*DDT, 4-NP, and 4-t-OP) showed enough potential to be considered EED agonists without performing any further Tier 2 tests. In AEDs, six chemicals (BF, CFD, GEN, LNG, P4, and SPR) as agonists and three chemicals (CPA, DZ, and 2-EHHB) as antagonists were recommended for Tier 2 tests (Tables 5, 6). Moreover, our literature search showed that MT was studied in eight articles and probably did not require Tier 2 tests anymore. However, P4 and LNG, as progestins, induced secondary sexual features in female Japanese medaka (XX) (Onishi et al., 2021), and further evaluation by Tier 2 tests is necessary. Among TEDs, two antagonists (DIC and EHMC) were recommended for Tier 2 tests and for MOS, four chemicals (OCL, FPN, RCT, and TRI), as stimulators, and three chemicals (BP, FAD and LET), as inhibitors, were recommended for Tier 2 tests. Taken together, among the 83 EDCs that targeted EATS pathways, 43 chemicals were recommended for Tier 2 tests, and 13 chemicals can be considered potential EDCs without any further Tier 2 tests in Japanese medaka.

Our literature search did not classify the EATS pathways of 45 chemicals (35%), even though several of them induced specific EATS-related apical endpoints (Table 11). Generally, in *in vivo* studies, probably due to the HPG and HPT axes, the overlapping effects of the chemicals within the EATS pathways cannot be ruled out; therefore, many of these unclassified chemicals demonstrated effects on endocrine-related apical endpoints, such as alteration in the liver VTG content (upregulated by CMP, CHDM, IBP, MTZ, NPX, 1NT, PDM, and RLX, and downregulated by DIBP), upregulation of *chg* in the liver of male fish (OXY and SRF), impaired reproductive activity and gonad histology (Cd, LD-BP, and nZnO), histopathological changes in the thyroid (BDE-47), inhibition of aromatase (TBT), and regulation of the E2 concentration in the blood of fish (Cd and SFT) (Table 11). In addition, several of the unclassified EDCs have potential as ESR agonists (CMP, DIBP, and FU) or antagonists (CYN, PHT, and RLX), and the ESR agonist and ARβ agonist (INT) and ESR agonist and ARβ antagonist (CMP) were observed in *in vitro* RGA with medaka *esr1* and *arβ* genes (Onishi et al., 2021; Kawashima et al., 2022). Moreover, the nanocarbon, GO, was evaluated in 10 articles targeting the gonads, thyroid, interrenal glands, and pancreas in adults; and gonads, thyroids, and interrenal glands in larvae (Dasmahapatra et al., 2020a; Dasmahapatra et al., 2020b; Dasmahapatra and Tchounwou, 2022a; Dasmahapatra and Tchounwou, 2022b; Dasmahapatra and Tchounwou, 2023a; Dasmahapatra and Tchounwou, 2023b; Asala et al., 2021; 2022; Myla et al., 2021a; b). Despite the histopathological alterations and cellular disruptions induced in the gonads, liver, kidneys, thyroid, interrenal glands, and pancreas of the adults and larvae of Japanese medaka by GO, due to the lack of specific Tier 1 and Tier 2 tests, GO remained unclassified without identifying any EATS-specific pathways (Dasmahapatra et al., 2020a; Dasmahapatra et al., 2020b; Dasmahapatra and Tchounwou, 2022a; Dasmahapatra and Tchounwou, 2022b; Dasmahapatra and Tchounwou, 2023a; Dasmahapatra and Tchounwou, 2023b; Asala et al., 2021; 2022; Myla et al., 2021a; b). Therefore, we think that, before excluding the potential of these unclassified chemicals as an ED, further validations using tier-based approaches are necessary. Alternatively, the effects should be considered nonspecific, mediated through oxidative stress, or not related to EATS-specific mechanisms.

Although the effects of 128 EDCs in Japanese medaka are classified based on EATS modalities, the disruptions of non-EATS pathways by these chemicals need to be investigated carefully (Martyniuk et al., 2022). Moreover, compared to EATS, less attention has been given to other endocrine organs, including the endocrine pancreas and the interrenal gland (adrenal gland), which should belong to non-EATS pathways of Japanese medaka. Due to the lack of validated *in vivo* or *in vitro* methods and the availability of the appropriate literature in the public databases, the evaluation of EDCs targeting non-EATS modalities of Japanese medaka is not properly focused on this review. Our literature search on the effects of EDCs on the endocrine pancreas and interrenal glands of Japanese medaka found only four articles, two for pancreas (Dasmahapatra and Tchounwou, 2023a; Dasmahapatra and Tchounwou, 2023b), and two for interrenal glands (Dasmahapatra and Tchounwou, 2022a; Dasmahapatra and Tchounwou, 2022b) (Tables 2, 11) in PubMed (www.ncbi.gov). Therefore, despite the significant importance of non-EATS modalities in Japanese medaka, due to the lack of sufficient literature and standard methods, the evaluation of EDCs mediated through non-EATS pathways is not appropriately described in this review article.

In conclusion, our strategies on the literature survey sorted 205 articles on Japanese medaka (*O. latipes*) that focused on 128 chemicals as EDCs. We found that 83 chemicals (∼65%) show potential as EDs targeting the EATS pathways. Although the overlapping of the endocrine-related apical endpoints cannot be ruled out, from the literature search, we classified 32 chemicals from 108 articles as EEDs, 22 chemicals from 46 articles as AEDs, 15 chemicals from 19 articles as TEDs, and 14 chemicals from 26 articles as MOS, and 45 EDCs from 60 articles remained unclassified. The number of EATS chemicals arranged in order (MOS < TED < AED < EED) fits well with the numbers identified by the literature search (TED < MOS < AED < EED). Moreover, 43 EDCs belonging to EATS are recommended for Tier 2 tests (∼34%), and 13 chemicals showed enough potential to be considered EDCs without any further tier-based studies (∼10%). Our evaluation of EDCs in Japanese medaka shows significant potential to further apply the laboratory-based research data for applications in regulatory risk assessments in humans.

## Figures and Tables

**Table:** 

**ACT**	Acetaminophen
**ATBC**	Acetyl tributyl citrate
**APEO**	Alkylphenol polyethoxylate surfactants
**AMT**	Amitrole
**AChE**	Acetylcholinesterase enzyme
**11-OA**	5α-Androstan-3,11,17-trione
**AED**	Androgen endocrine disruptors
**AR**	Androgen receptor
**AR-1260**	Aroclor 1260
**ATZ**	Atrazine
**AZM**	Azinphos-methyl
**BP2**	Benzophenone 2
**BP3**	Benzophenone 3
**BF**	Bifenthrin
**DEHS**	Bis(2-ethylhexyl) sebacate
**BPA**	Bisphenol A
**BZT-UV**	Benzotriazole ultraviolet stabilizer
**BKC**	Benzalkonium chloride
**i-BP**	i-Butylparaben
**n-BP**	n-Butylparaben
**Cd**	Cadmium
**CFD**	Cefadroxil
**CFR**	Cefradine
**ClxBPA**	Chlorinated BPA
**CMP**	4-Chloro-3-methylphenol
**CLT**	Chlorothalonil
**CTC**	Chlortetracycline
**CHG**	Choriogenin
**CYN**	Cyanazine
**CHDM**	1,4-Cyclohexanedimethanol
**CPA**	Cyproterone acetate
**dpf**	Day post-fertilization
**dph**	Day post-hatch
**DZ**	Diazinon
**DBP**	Dibutyl phthalate
**DIC**	Diclofenac
**p,p′-DDE**	Dichlorodiphenyldichloroethylene
**o,p′-DDT**	Dichlorodiphenyltrichloroethane
**DEHP**	Diethylhexyl phthalate
**DES**	Diethylstilbestrol
**DHT**	5α-Dihydrotestosterone
**DIBP**	Diisobutyl phthalate
**EDs**	Endocrine disruptors
**EDSP**	Endocrine Disruptor Screening Program
**EDS**	Endosulfan
**END**	Endrin
**E2**	17-β-Estradiol
**E3**	Estriol
**ESR**	Estrogen receptor
**E1**	Estrone
**EQ**	Equol
**EAS**	Estrogen–androgen–steroidogenesis
**EATS**	Estrogen–androgen–thyroid–steroidogenesis
**EHMC**	2-Ethylhexyl-4-methoxycinnamate
**EFSA**	European Food Safety Authority
**EMA**	European Medicine Agency
**EE2**	17α-Ethinylestradiol
**EU**	European Union
**FAD**	Fadrozole
**FNT**	Fenitrothion
**FNC**	Fenoxycarb
**FV**	Fenvalerate
**FLFII**	Female leukophore-free strain
**FAT**	Fish acute toxicity test
**FELS**	Fish, early life stage toxicity test
**FET**	Fish embryo toxicity test
**FSTRA**	Fish short-term reproduction assay
**FPN**	Fipronil
**FLX**	Fluoxetine
**FLR**	Fluridone
**FLU**	Flutamide
**GEN**	Genistein
**GLP**	Glyphosate
**gsdf**	Gonadal soma-derived factor
**GSI**	Gonadosomatic index
**GO**	Graphene oxide
**HSI**	Hepatosomatic index
**HBCD**	Hexabromocyclododecane
**hpf**	Hour post fertilization
**HPA**	Hypothalamus–pituitary–adrenal axis
**HPG**	Hypothalamus–pituitary–gonadal axis
**HPT**	Hypothalamus–pituitary–thyroid axis
**IBP**	Ibuprofen
**KC-400**	Kanecholor 400
**KTC**	Ketoconazole
**11-KT**	11-Ketotestosterone
**LC**	Lethal concentration
**LET**	Letrozole
**LNG**	Levonorgestrel
**LD-BP**	Lignin-derived bisphenol
**LIN**	Lincomycin
**LNR**	Linuron
**LOEC**	Lowest observed effect concentration
**LOEL**	Lowest observed effect level
**MEOGRT**	Medaka extended one-generation reproduction test
**MELA**	Medaka embryo-larval development assay
**MDA**	Malondialdehyde
**MET**	Metformin
**MTZ**	Methimazole
**MXC**	Methoxychlor
**4-MBC**	3-(4-Methylbenzylidene) camphor
**MPB**	Methylparaben
**MT**	Methyltestosterone
**MTC**	Metolachlor
**MOE**	Ministry of Environment
**mph**	Month post-hatch
**MOS**	Modulator of steroidogenesis
**MCB**	Myclobutanil
**NPX**	Naproxen
**1NT**	1-Naphthol
**NDEA**	N-Nitrosodiethylamine
**NSAID**	Nonsteroidal anti-inflammatory drug
**4-NP**	4-Nonylphenol
**NP1EC; NP2EC**	Nonylphenol ethoxycarboxylate
**NP1EO; NP2EO; NP9EO**	Nonylphenol ethoxylate
**NPEO**	Nonylphenol polyethoxylates
**NOEC**	No-observed-effect concentration
**OCL**	Octocrylene
**OMC**	Octyl methoxycinnamate
**4-OP**	4-Octylphenol
**OECD**	Organization of Economic Cooperation and Development
**OYZ**	Oryzalin
**OXF**	Oxyfluorfen
**OTC**	Oxytetracycline
**PDM**	Pendimethalin
**PCPL**	Pentachlorophenol
**4t-PP**	4-Tert-pentylphenol
**PFAA**	Perfluoroalkyl acid
**PFBA**	Perfluorobutyric acid
**PFOA**	Perfluorooctanoic acid
**PFOS**	Perfluorooctane sulfonate
**PFOSA**	Perfluorooctane sulfonic acid
**PFAA**	Perfluoroalkyl acid
**PFBS**	Perfluorobutane sulfonate
**PFNA**	Perfluorononanoic acid
**PPAR**	Peroxisome proliferator-activated receptor
**PHT**	Phenytoin
**PCB 126**	Polychlorinated biphenyl 126
**PCZ**	Procloraz
**P4**	Progesterone
**PRN**	Propanil
**PPB**	Propylparaben
**PTU**	6-Propyl-2-thiouracil
**RCT**	Ractopamine
**RLX**	Raloxifene
**REP**	Relative estrogenic potency
**RGA**	Reporter gene assay
**RND**	Roundup
**SERM**	Selective estrogen receptor modulator
**SDS**	Sodium dodecyl sulfate
**SPC**	Sodium perchlorate
**SPR**	Spironolactone
**SFT**	Sulfathiazole
**SRF**	Surflan
**TAM**	Tamoxifen
**TPA**	Terephthalic acid
**TG**	Test guidelines
**4t-OP**	4-Tert-octylphenol
**T**	Testosterone
**TBBPA**	Tetrabromobisphenol A
**TBCO**	1.2,5,6-Tetrabromocyclooctane
**BDE-47**	2,2′,4,4′Tetrabromodiphenyl ether
**TU**	Thiourea
**TED**	Thyroid endocrine disruptors
**TH**	Thyroid hormone
**TR**	Thyroid hormone receptor
**TSH**	Thyroid-stimulating hormone
**T4**	Thyroxine
**TRA**	17α-Trenbolone
**TRB**	17β-Trenbolone
**TRD**	Triadimenol
**TRF**	Triadimefon
**TBT**	Tributyltin
**TCS**	Triclosan
**TCrP**	Tricresyl phosphate
**TRF**	Trifloxystrobin
**T3**	Triiodothyronine
**TRI**	Trilostane
**TPhP**	Triphenyl phosphate
**TPT-Cl**	Triphenyltin chloride
**TDCPP**	Tris (1,3-dichloro-2-propyl) phosphate
**USEPA; EPA**	United States Environmental Protection Agency
**USFDA**	United States Food and Drug Administration
**VIN**	Vinclozolin
**VTG**	Vitellogenin
**wph**	Week post-hatch
**nZnO**	Nano zinc oxide
**ZnSO4**	Zinc sulfate

## Data Availability

The datasets presented in this study can be found in online repositories. The names of the repository/repositories and accession number(s) can be found in the article/Supplementary Material.
