# Supplementary material for: A systematic review of the evaluation of endocrine-disrupting chemicals in the Japanese medaka (Oryzias latipes) fish
Source: Front Toxicol. 2023 Nov 27;5:1272368. doi: 10.3389/ftox.2023.1272368 (PMC10711633; doi:10.3389/ftox.2023.1272368)
Supplement: Supplementary file 1 [file Table1.pdf]

Supplementary Table-1 (ST-1): List of chemicals showing endocrine disrupting activities on medaka.

Serial number	Chemicals	Experimental conditions	Observations	References
1.	Acetaminophen (ACT)	.095, .95, 9.5, 95, 950, 9500, 95000 µg/L; fertilized eggs were exposed until 90dph	i) Survival of eggs and larvae were not affected<br>ii) Survivability of juveniles were reduced in a concentration-dependent manner<br>iii) Growth remained unaffected<br>iv) HSI and GSI remained unaffected<br>v) Non-linear increase in hepatic VTG in male fish	Kim et al., (2012)
2.	Acetyl tributyl citrate (ATBC)	100, 320, 1000, 3200, 10,000 µg/L, embryos (4hpf) exposed until swim bladder inflation (1 dph)	i) Hatching delay increased and decrease in hatching rate significantly decreased in the last two highest concentrations.<br>ii) Body curvature and edema occurred in the last two concentrations.<br>iii) Swim bladder inflation significantly lower in last three concentrations<br>iv) Induced growth inhibition.	Horie et al (2022b)

			v) No change in expression of *tshβ*, and *dio1*<br>vi) Suppression of *trα* occurred in all treatment groups.<br>vii) *trβ*, and *dio2* suppressed in 100, 320, and 1000 µg/L groups	
		0.1, 0.32, 1, 3.2 mg/L; embryos exposed 4hpf until 40 dph	i) LOEC is 1046.6 µg/L and NOEC is 392.6µg/L<br>ii) did not induce intersex or sex change<br>iii) the vtg1 and vtg2 expression in the liver of XX was reduced	Horie et al., (2023b)
3.	Amitrole	62.5, 125,250,500, 1000 µg/L, embryos (4hpf) exposed until 8dpf (stage 38)	i) *gsdf* expression in XX and XY embryos remained unaltered	Horie et al., (2022a)
4.	5α-androstan-3,11,17-trione (11-OA)	0.1, 1.0, 10, and 100 µg/L, 8-month-old fish, exposed for 21 days	i) Ratio of male medaka increased (significant at higher concentrations)<br>ii) NOEC=0.1 µg/L; LOEC=1.0 µg/L	Grillitsch et al., (2010)
5.	Arochlor 1260 (AR-1260)	100 µg/L; adult male fish exposed for 24h	i) Enhanced expression of *vtg1*, *chgL*, *chghHm* in liver of male fish	Yum et al., (2010)
6.	Atrazine (AT)	100 µg/L, Adult fish exposed for 3 weeks	*i)* *Esr1* transcripts were significantly decreased in the testis.	Zhang et al., 2008a

		0.5, 5.0, and 50 µg/L, adult fish exposed for 14 and 38 days	*i)* Egg production decreased.<br>Brain:<br>*ii)* Female brain: *cyp19a1b* induced only in 0.5 µg/L.<br>Liver:<br>*iii)* Female liver *vtg* reduced in 0.5 and 5 µg/L.<br>*iv)* Female liver *zp3.1* reduced in 0.5 and 5 µg/L.<br>Gonad:<br>*v)* Male gonad: *cyp17a1* induced in 0.5, 5, and 50 µg/L (not concentration-dependent)<br>*vi)* Male gonad *cyp11a1* induced in 0.5 and 5.0 µg/g	Richter et al., 2016
7.	Azinphos-methyl (AZM)	0.06 µg/L, 0 d of embryos exposed until 7 dph, depurated for 5 months	i) Length reduced (7 dph)<br>ii) Did not affect survival, hatching time and foraging ability<br>iii) Female-biased sex ratio	Teather et al., (2005)
8.	Benzalkonium Chloride (BKC)	1.6, 2.1, 3.0, 10.8, 113.4 µg/L; fertilized eggs were exposed until 38dpf	i) No effect on hatchability, survival, and growth of the larvae<br><br>ii) the expression of *vtg1* mRNA in the whole body of the larvae was enhanced in a nonlinear	Kim et al., (2020).

			fashion, while *vtg2* remained unaltered	
9.	Benzophenone 2 (BP2)	0.0943, 0.939, 9.53 mg/L.<br>16±2 weeks medaka were exposed for three weeks (OECD TG229)	iii) Liver *vtg* induction in males and females is concentration dependent.<br>iv) No alteration of secondary sex characters<br>v) Fecundity and fertility reduced in 9.53 µg/L	Kawashima et al., (2022)
	Benzophenone 3 (BP3)	10,100, 1000 µg/L, 3-month-old fish exposed for 21 days	i) Hepatic vitellogenin in male fish significantly increased (1000µg/L), remained unaltered in female.<br>ii) Hatching rate reduced	Coronado et al., (2008)
		EXPOSURE1: 15,50,150, 500 µg/L, Adult fish exposed for 14 days<br><br>EXPOSURE 2: 15,50,150 µg/L F0 fish exposed for 28 days, F1 eggs (0 hpf) exposed until 30 dph	Exposure 1<br>i) After 14 days of exposure (adult fish) plasma concentration of testosterone (T) increased in male fish (F0)<br>ii) Ratio of E2: T decreased in both male and female fish (F0)<br>iii) Transcription of *cyp19b* and *erβ* genes in the brain of both male and female fish are downregulated.	Kim et al., (2014)

			iv) Transcription of *vtg1* and *vtg2* in the liver of male fish (90 µg/L), not in female fish<br>vi) Down regulation of gonadal steroidogenic genes such as *StAR*, *cyp17*, *hsd 3b*, *hsd17b3*, *cyp19a* (F0).<br>EXPOSURE 2:<br>vii) After 28 days exposure, the daily average egg production per female was reduced at 26 µg/L; hatchability of F1 eggs not affected.<br>viii) After 30 dph exposure, no change in mortality (F1)	
10.	Benzotriazole ultraviolet stabilizer (BZT-UV)	36, 158, 634 ng/g food, adult fish exposed for 28 days.	i) No significant effect on egg production or fertilization success.<br>*ii)* No significant effect on the expression of hepatic *erα, vtg1, cyp1a*, or *cyp3a4* either in males or females.<br>*iii)* *cyp11a*, *cyp19a* were significantly lower (down regulated) in gonads (ovary) of female fish fed with 634 ng BZT-UV/g food.	Fujita et al., (2022)

			i) 11-KT remained unaltered in plasma.	
11.	Benzylparaben (BZP)	4,20,100, 500 µg/L; Adult male fish (2.5-month-old) exposed for 14 days	ii) Concentration-dependent increase in plasma VTG level in male fish (similar to that of female blank in male fish exposed to 500µg/L)<br>iii) Concentration-dependent up- (#6-41) and downregulation (#2-21)of genes .<br>iv) The upregulated genes arevtg1,vtg2,chgHm, chgL chgH esr1,cyp1a	Yamamoto et al., (2007)
12.	Betamethasone (BMT)	20 and 200 ng/L; 2 dph larvae were exposed for 110 days (F0); reproductive activity (exposed F0 males mated with unexposed F0 females) to get F1 fish	i) Body weight in males reduced in a concentration-dependent manner<br>ii) Testis weight decreased in a concentration-dependent manner<br>iii) No alteration in sexratio, however, ovotestis observed in male fish<br>iv) Expression of fshβ and lhβ decreased in the pituitary<br>v) The FSH and LH content in the pituitary decreased	

			vi) Upregulation of 17β-hsd3, 3β-hsd, cyp17 and cyp19a1, cyp11b1, and 11β-hsd2 mRNAs in testis<br>vii) Concentration of T in plasma was decreased while 11-KT, E2, and ARO were increased<br>viii) Expression of vtg1 and vrg2 was increased in liver of male fish and the hepatic VTG was also increased.<br>ix) Exposure of male fish to betamethasone impaired reproductive activities.	
13.	Bifenthrin (BF)	0.15 and 1.5 µg/L, 8 dph larvae exposed for 30 days, depurated until 8 weeks (at sexual maturity)	i) Decrease in survival<br>ii) Tended to induce masculinization as observed in anal fin papillary processes (only in 0.15 µg/L)<br>iii) Genotypic gender ratio did not alter.	Bertotto et al., (2019)
14.	Bis(2-ethylhexyl) sebacate (DEHS)		i) Expression of *tshβ*, *dio1*, and *trα* did not change.<br>ii) Expression of *dio2* suppressed.<br>iii) No induction of swim bladder inflation	Horie et al., (2022c)

			iv) No alteration in expression of *kiss1*, *gnrh, fshβ, chgH*, and *vtg* in a concentration dependent manner	
15.	Bisphenol A (BPA)	0.3, 1, 3, 10 µM/L, reproductively adult male fish exposed for 14 d.	i) Reduced egg production and hatching (10 µmol/L)	Shioda and Wakabayashi (2000)
		2.28, 13.0, 71.2, 355, and 1820 µg/L; fertilized eggs exposed until 60 dph.	i) No effect on hatching, hatching time, embryo morphology and mortality<br>ii) Total length and weight suppressed in concentration dependent manner<br>iii) Secondary sex characteristics of males disappear in 1820 µg/L with 32% of fish have testis-ova.<br>iv) LOEC is in between 355-1820 µg/L	Yakota et al., (2000)
		10, 50, 100, 200 µg/L; 1-90 dph	i) Length, weight, and sex ratio did not change.<br>ii) Testis-ova (8%) only in larvae exposed to 10 µg/L<br>iii) NOEL for testis-ova is 5.9 µg/L<br>iv) LOEL for testis-ova is <5.9 µg/L)	Metcalfe et al., (2001)

		0.1, 10, and 100 µg/L, mature male medaka exposed for 5 weeks;<br>0.1, 1.0, 25, 50 and 100 µg/L, hatched embryos exposed for 230 days	i) LC_{50} for adult fish is 7.5mg/L; serum vtg induction (female-specific protein) occurred in adult males (10 µg/L)<br>ii) LC_{50} values for embryos 5.1 mg/L; sex ratio did not shift towards female; abnormal testis (100 µg/L) observed.	Tabata et al., (2001)
		1,2, and 4 mg/L, adult male and female medaka exposed for 3 weeks	i) Fecundity, fertility, and sex ratio remained unaltered.<br>ii) Testis-ova observed and VTG in liver increased in males.	Kang et al, (2002b)
		0.1, 10 and 100 µg/L, mature male medaka exposed for 5 weeks.	i) LC_{50} values for mature male and female medaka (exposed for 72h) is 6.8 mg/L for males and 8.3 mg/L for females.<br>ii) LC_{50} values for embryos 5.1 mg/L<br>iii) IC_{50} for inhibition to egg hatching are 9.0 mg/L<br>iv) Male medaka were exposed to 0.1, 10, or 100 µg/L for 5 weeks induced female-specific protein was induced	Kashiwada et al., (2002)

			with concentrations 10 µg/L and above.	
		5, 50, 100, 200 and 500 µg/L, mature male medaka exposed for 144 h.	i) *chgL* mRNA was induced in 50 -500 µg/L in a concentration-dependent manner.<br>ii) *chgH* mRNA expression was observed in 100 µg/L	Lee et al., (2002)
		50,200, 1000, 5000 µg/L; transgenic adult males were exposed for 21 days	i) *vtg1* mRNA expression was induced in fish exposed to 200-5000 µg/L)	Zeng et al., (2005)
		0.5, 1, 2, and 4 mg/L; adult reproductively active male, exposed for 48h	(i) Concentration-dependent increase of *vtg1* and *vtg2* and *chgH* and *chgL* in the liver of male<br>(ii) The minimum effective dose of 0.5 mg/L for *chgH* and *vtg2*, and 2 mg/L for *vtg1* and 4 mg/L for *chgL*	Kamata et al., (2011)
		0.1, 1, 10, 100 ng/mL; transgenic medaka embryos 5 hpf exposed until hatching.	i) *gnrh1*, *gnrh2*, *gnrh3* gene expression down regulated at highest BPA concentration (100 ng/mL).<br>ii) *esr1*, *esr2a*, *esr2b* mRNA expression remained unaltered.	Lee et al., (2012)

			iii) *gnrh-R1*, *gnrh-R2*, *gnrh-R3* mRNAs remained unaltered.<br>iv) Effects on heart rate (LOEC 2dpf=10 ng/mL, 4dpf=2ng/mL) eye pigmentation (LOEC 2dpf=1 ng/mL; 4 dpf < 100 ng/mL), head growth (LOEC=100 ng/mL) hatching time (LOEC=1 ng/mL) significantly affected.	
		8.5 mg/L; 3-4 hpf embryos exposed until 7 dpf	i) EC_{50} is 9.8 mg/L.<br>ii) *cyp19a1b*, *esr2a*, *lss*, *sc4mol*, and *mvd* are induced; *vtg1*, *sc4mol*, *ar, 3β-hsd*, *cyp11b*, *gnrh2* gene expressions remained unaltered.	Schiller et al., (2014)
		100 µg/L, 8hpf embryos exposed for first 7 days	i) No apparent phenotypic abnormalities in F0 and F1 generation<br>ii) Significant reduction in fertilization rate in F2<br>iii) Reduction of embryo survival in F3	Bhandari et al, (2015)
		In vitro reporter gene assay; gene transcriptional activities for ESR subtypes	i) ESR1= EC_{50}= 7.88X 10^{-7}M [[relative potency=0.017, considering estrogen as 100]	Tohyama et al., (2015)

			ii) ESR2a: EC_{50}=6.11 X 10 $^{-7}$ M (relative potency=0.0053, considering estrogen as 100)<br>iii) ESR2b= not detected.	
		200 ng/L,<br>1 dpf embryos of transgenic medaka are exposed until 20 dpf	i) Increased fluorescence intensity of the GnRH3-GFP neuronal population of the terminal nerve (TN) of the forebrain at 3dpf but decreased intensity at 5 dpf<br>ii) Advanced eye pigmentation and accelerated hatching time.<br>iii) Suppression of locomotion in 20 dpf larvae (chronic exposure) both in speed of movement and distance covered.<br>iv) Decreased cell body sizes of individual TN-GnRH3 neurons but not in non -GnRH3 neurons	Inagaki et al., (2016)
		1.5 mg/L, Juvenile fish exposed for 60 days;	i) The 96h LC50 is 5.0 mg/L.<br>ii) Total number of eggs are 47% less than controls; total number	Li et al., 2016; 2017.

		8.5, 9, 9.5, 10, 12, mg/L for LC50 assay.	of broods were 46% lower than controls:<br>iii) GSI in female decreased.<br>iv) HSI unchanged.<br>v) Catalase activity was reduced only in ovary; other tissues (liver, gill, intestine, testis) no change.<br>ii) SOD unaltered, except intestine where 82% increase was observed; GST in liver and gills enhanced.<br>iii) MDA elevated in all five tissues (liver, gill, intestine, testis, ovary)<br>iv) AChE activity in liver reduced.<br>v) α-Gluconate in intestine was decreased by 69%<br>vi) Liver vtg both in males and females did not change.<br>vii) Testis had fewer spermatocytes and spermatids and slightly more spermatogonia than controls; intravascular proteinaceous fluid and germinal epithelium	

			hypertrophy were observed.<br>viii) In ovary, perifollicular cell hyperplasia and degeneration of chorion were observed.	
		45, 92, 326,1030, and 3406 µg/L, culture condition TG 234 [exposed 2hpf- stage 38, or until 30 or 60 dph]	i) At 1030 and 3406 µg/L concentrations, testis-ova are induced with female-type sexual characters in XY males on 30 and 60 dph.<br>ii) Suppressed (1030 and 3406 µg/L) *gsdf* mRNA expression and increased *cyp19a1a* mRNA expression in XY individuals at stage 38 and 30 dph, *foxl2* mRNA expression showed no change.<br>iii) The concentration of BPA that suppressed *gsdf* mRNA expression at the larval stage was consistent with that needed to induce testis-ova and sex reversal.	Horie et al., (2019)
		10 µg/L, 8hpf embryos exposed until 50 dpf	i) 651 differentially expressed genes (DEG)are revealed by transcriptional analysis.	Bhandari et al., (2020)

			ii) Gene ontology analysis (GO) showed enrichment of intracellular receptor signaling pathway, response to steroid hormone, and hormone mediated signaling pathways.<br>iii) Pathway analysis showed significant enrichment in integrin signaling pathway.<br>iv) Single nucleotide polymorphism (SNP) and insertion-deletion (Indel) analysis found no significant difference in mutation rates.	
		6.8-220 µM, 5hpf exposed for 14 days; 3.5-220µM, 1dph exposed for 96 h; 1.8-30 µM, 5hpf exposed for 7 and 9 dpf (hatched)	i) BPA at 54.5 µM concentration significantly decreased hatchability; embryos did not hatch when exposed to 110 and 220 µM.<br>ii) LC_{50} is 58 µM (larvae 96h); embryos 60 µM (14 days).<br>iii) LOEC in 14 dpf embryos regard to hatching is 55 µM; heart rate, 110 µM.	Ishibashi et al., (2020)

			iv) LOEC regarding estrogenicity in 7 dpf embryos is < 30 µM, 9 and 12 dpf larvae is 30 µM.<br>v) 7 days exposure of embryos (5 hpf to 7 dpf;1.8, 3.7, 7.5,15, 30 µM) or 9day larvae (exposure continued 5hpf-9hpf, until the embryos hatch) did not affect the expression *chgL*, *chgH*, *chgHm*, *vtg1*, *vtg2*, *cyp19a1b*, *esr1*, *esr2a* and *esr2b* genes.<br>vi) Larval exposure (9 dph [immediate hatching] to 12 dpf; 3 days exposure] enhanced *chgL*, *chgH*, and *cyp19a1b* genes at highest BPA concentration (30 µM).<br>vii) Relative estrogenic potency (REP) is $1X10^{-5}$ µM (considering E2 as 1).	
		100 µg/L, 8hpf- 7 dpf depurated to 150 dpf (F0); no further exposure in F1 and F2.	i) Hyperactive state of expression for kisspeptin (*kiss1* and *kiss2*) and their receptors (*kiss1r* and	Thayil et al., (2020)

			kiss2r) and *gnrh* and *gnrh* receptors (F2 generations)<br>ii) *Dnmt1* expression decreased in brain (F2 generation)<br>iii) Global hypomethylation pattern was observed in the testis of both F0 and F2 generation fish.	
		0.2, 1.0, 5 mg/L; 16±2 weeks medaka were exposed for three weeks (OECD 229)	i) HSI and hepatic vitellogenin increased in both male and females in highest dose (4.67 mg/L)<br>ii) Secondary sex characters did not change in both males and females<br>iii) Fertility rate decreased in highest dose (4.67 mg/L)	Kawashima et al., (2021)
16	i-Butylparaben (i-BP)	4,20, 100, 500 µg/L; Adult male fish (2.5 months old) were exposed for 14 days	i) Concentration-dependent enhancement in the serum VTG content of male fish	Yamamoto et al., (2007)
17	n-Butylparaben (n-BP)	8, 40, 200, 1000 µg/L; male fish exposed for 14 days.	i) Concentration-dependent enhancement in the serum VTG content of male fish	Yamamoto et al., (2007)
18.	Cadmium (Cd)	1, 5, and 10 µg/L, adult fish exposed for 7 weeks	ii) No change in plasma VTG, testosterone, or hepatic ER in both male	Tilton et al., (2003)

			and females after 7 weeks exposure.<br>iii) GSI in males decreased at 5 µg/L, 7 weeks exposure<br>iv) Plasma E2 enhanced in females at 5 µg/L but reduced significantly in 10 µg/L (7 weeks). No significant alterations in males<br>v) Gonadal estrogen and testosterone decreased in both males and females in all concentrations of Cd (7 weeks)	
		10 mg/L, adult males exposed for 96 h	i) The mortality rate enhanced with the duration of exposure.<br>ii) Numerous apoptotic cells were found in the spermatogonia and spermatocytes at 72 and 96 h after initiation of cadmium exposure.<br>iii) The Pyknotic index, TUNEL-positive rate, and elevated caspase-3-positive rate in the SPG and SPT of the cadmium treated groups are higher.	Hirako et al., 2017

			iv) Phospho-histone H3-positive rate in the SPG and SPT remained unaltered.<br>v) No edematous, hemorrhagic, or necrotic changes were observed within the testis.	
19.	Cefadroxil (CFD)	1, 7.8, 84.8, 718.9, 8,883.1 µg/L; four-month exposure study	i) No effect on hatching<br>ii) Survivability of the larvae and adults decreased in a concentration-dependent manner<br>iii) Plasma E2 was decreased in male fish and enhanced in female fish in a concentration-dependent manner<br>iv) The expression of *gnh2, gnrhr1, gnrh2, lhδ, esr1, esr2a*, and *cyp19b* mRNA in the brain and *fshr, lhr, StAR*, and *3βhsd* in the testis was significantly upregulated while *cyp19a* mRNA in the testis was downregulated.<br>v) In females, the expression of *gnrh1, gnrh2, gnrhr1, esr2a,*	Kim et al., (2017)

			and *cyp19b* in the brain, and *fshr, cyp11a, 3βhsd, cyp17*, and *cyp19a* in the ovary was upregulated, while the expression of *lhβ*, *hmgr*, and *StAR* mRNAs was decreased.	
20.	Cefradine (CFR)	1, 7.1, 73.9, 724.6, and 7, 758.5 µg/L; embryos were exposed and evaluated four months period.	i) Hatching unaffected<br>ii) Fecundity reduced in a concentration-dependent manner<br>iii) Plasma E2 level remained unaltered in males and increased in females<br>iv) Expression of *gnrh2*, *gnrhr1*, *esr1*, *esr2a*, and *cyp19b* mRNAs in the brain, and *hmgr*, *StAR*, *cyp11a, 3βhsd*, and *cyp19a* mRNAs in the testis of male fish significantly downregulated<br>v) In females, *gnrh2, gnrhr1, lhβ, lhβ, esr1 ,esr2a, cyp19b* mRNAs in the brain, and *fshr*, *lhr*, *hmgr*, *StAR*, *cyp11a*, and *3βhs*d mRNAs in	Kim et al., (2017)

			ovaries were downregulated.	
21	Chlorinated bisphenol A (2-(3-chloro-4-hydroxyphenyl)-2-(4-hydroxy-phenyl) propane (3-ClxBPA)	100, 200, 500 µg/L; Adult males were exposed for 5 weeks	i) LOEC was 500 µg/L; NOEC was 200 µg/L<br>ii) Concentration-dependent serum VTG induction occurred after 2 weeks of exposure	Tabata et al., (2004)
	2,2-bis(3-chloro-4-hydroxyphenyl) propane (3,3'-diClxBPA)	100, 200, 500, 1000 µg/L: Adult males were exposed for 5 weeks	i) LOEC was 500 µg/L; NOEC was 200 µg/L<br>ii) Mortality rate depends on the concentration and duration of exposure<br>iii) Concentration-dependent serum VTG induction occurred after 3 days of exposure	
	2-(3,5-dichloro-4-hydroxyphenyl)-2-(3-chloro-4-hydroxyphenyl) propane (3,3', 5-triClxBPA)	200, 500 µg/L: Adult males were exposed for 5 weeks	i) VTG was detected after 5 weeks of exposure in fish exposed to 500 µg/L	
	2,2-bis (3,5-dichloro-4-hydroxyphenyl) propane (3-,3'.5,5'-tetraClxBPA)	1000 µg/L: Adult males were exposed for 5 weeks	i) VTG induction in serum did not occur	
22.	4-chloro-3-methyl-phenol	20.7, 43.3, 88.0, 165.1, 344.7 µg/L; MEOGRT protocol (multigenerational exposure)	i) No mortality: the LOEC for larval and adult survival was >345 µg/L.<br>ii) LOEC for fecundity, fertility, hatching, liver VTG content, secondary sexual features, body weight was >345 µg/L.	Flynn et al., (2017)

			iii) Liver vtg was induced in F0 fish (XX) at 88, 165 and 344 µg/L<br>iv) Liver vtg was decreased in F2 adults exposed to 345 µg/L<br>v) In F1 fish (XY) the length and weight were decreased in fish exposed to 165 and 345 µg/L	
		108, 350, 1060 µG/L; adult fish exposed for 21 days	i) No mortality<br>ii) Length and weight of the male and female fish remained unaltered.<br>iii) Liver weight (HSI) and gonad weight (GSI) remained unaltered in both male and female fish.<br>iv) Liver vtg in both male and female fish increased in a concentration-dependent manner.<br>v) Secondary sexual features (papillae in the anal fin) remained unaltered.<br>vi) Number of total eggs, fertilized eggs and fertility rate remained unaltered	Onishi et al., (2021)

23.	Chlorothalonil (CLT)	0.06 µg/L,<br>0 dph fries exposed for 7 days,<br>depuration 5 weeks.	i) Did not affect survival, hatching time, and foraging activity.<br>ii) The length of larvae reduced 7dph.<br>iii) Female biased sex ratio	Teather et al., (2005)
24.	Chlortetracycline (CTC)	0.1,1.,10, and 100 mg/L, adult fish (male and female) exposed 3-5 days	i) vtg induction occurred in male fish (10 mg/L) and above.	Kim et al., 2007
		4 and 40 mg/L, male fish exposed for 14 days	i) Blood E2 did not change in 4 mg/L group, however, enhanced in 40 mg/L	Ji et al., (2010).
25.	Cyanazine (CYN)	0.110, 0.349, 1.02 mg/L, adult fish (16±2 weeks) exposed for 3 weeks	i) Hepatic vitellogenin unaltered both in males and females.<br>ii) HSI decreased in in last two doses in both males and females (0.349, 1.02 mg/L)<br>ii) Secondary sex characters in male and female remained unaltered.<br>iii) Number of total eggs, fertilized eggs, and fertility rate inconclusive.	Kawashima et al., (2021)
26.	1,4-cyclohexanedimethanol (CHDM)	0.1. 1, and 10 mg/L, fertilized eggs exposed for 40 days	i) Significant decrease in hatchability and juvenile survival (10 mg/L)	Jang and Ji, (2015)

			ii) Body weight reduced in juveniles (1mg/L and greater)<br>iii) *vtg2* mRNA significantly upregulated in a concentration-dependent manner<br>iv) *cyp19b*, *esr1*, *vtg1*, *StAR*, *cyp11a*, *cyp17*, *hsd3b*, *cyp19a* mRNAs remained unaltered	
27.	Cyproterone acetate (CPA)	1, 10 µg/l, 1 dph fries exposed for 100 days	i) Spermatogenesis and oogenesis inhibited moderately.<br>ii) No difference in sex ratio<br>iii) Testis-ova observed in male fish in a concentration-dependent manner.	Kiparissis et al., (2003b)
28.	Diazinon (DZ)	1, 5,7, 9, 13,17, 22, 26 mg/L;<br><br>EXPOSURE 1: Fertilized eggs 1 dpf (stage 11), 3 dpf, and 5 dpf eggs were exposed for 96h and observed after hatching (10 dpf)<br><br>EXPOSURE 2:	i) Decrease in hatching<br>ii) Decrease in total length<br>iii) Decrease in swim bladder inflation<br>i) Edema, pericardial sacs and vitelline veins were increased in a concentration-dependent manner	Hamm and Hinton, (2001)

		Fertilized eggs 1 dpf and 3dpf exposed until 9 dpf		
		2.9, 5.2, 10.3,19.8, 40.2 µg/l; MEOGRT protocol (OECD TG 240)	i) LOEC for fecundity is 2.9 µg/L<br>ii) LOEC for growth is 5.2 µg/L<br>iv) Number of anal fin papillae in F1 males tended to be significantly lower in male fish compared to controls in a concentration-dependent manner.<br>v) *vtg* gene expression in liver did not alter<br>vi) pathological abnormalities was observed in kidney not in gonads	Flynn et al., (2018)
		38, 196, 598, 952 µg/L, 16±2 weeks old fish were exposed for three weeks (TG229)	ii) Secondary sex characters in male and females unaltered<br>iii) HSI and GSI decreased in both males and females at 196, 598, 952 µg/L concentrations.<br>iv) The number of total eggs, fertilized eggs and fertility rate reduced in higher concentrations.	Kawashima et al., (2021)

			v) Hepatic vtg remained unaltered in males enhanced in females (not concentration-dependent)	
29.	Dibutyl phthalate (DBP)	25.6, 64, 160, 400, 1000 µg/L; male and female fish exposed for 21 days.	i) HSI increased in males but not in females.<br>ii) Liver VTG remained unaltered after DBP exposure	Nozaka et al, 2004
30.	*p,p′*-DDE	1,5, 20, 100 µg/L, 20 dph male larvae exposed for 2 months	i) HSI increased and GSI decreased.<br>ii) Intersex was found in 100 µg/L<br>iii) Upregulation of *vtg1, vtg2, chgH, chgL, esr1* in the liver	Jhang and Hu, 2008
		1,3.2,10, 32,100 µg/L, 4 hpf embryos exposed until stage 38 (8dpf)	i) LOEC for intersex induction 32.4 µg/L<br>ii) No statistically significant difference was observed in *gsdf* expression in XY embryos	Horie et al., (2022)
31.	*o,p′*-DDT	20, 33, 237, 1079 ng /egg;<br><br>fertilized eggs injected and depurated for 10 weeks	i) 50 % mortality (511±22 ng/egg)<br>ii) 86% genetic males sex reversed to female (XY) phenotypes (227±22 ng/egg)<br>iii) 50% of the XY females produce eggs if paired	Edmunds et al., (2000)

			with XY males and hatched to viable larvae.	
		0.5, 1.0, 2.5, and 7.5 µg/L,<br>2 or 8 wph larvae exposed for 2,4, or 8 weeks and then grown to sexual maturity	i) No effect on vtg expression after 2 weeks exposure<br>ii) Induced vtg after 8 weeks exposure.<br>iii) Ovotestis were observed after 2 or 4 weeks exposure to the two highest doses.<br>iv) Both 2 and 8 weeks exposure reduced fertility and hatching success at all doses with lower dose having a greater effect after longer exposure.	Cheek et al., (2001)
		7.5 µg/L, 0 dph fries exposed for 5,9, and 14 days.	i) The adult sex ratio altered after 2 weeks exposure<br>ii) mRNA expression in brain aromatase (*cyp19b*) and activity (protein) enhanced	Kuhl and Brouwer, (2006)
		1 and 100 µg/L, 3 months old male fish exposed for 48 h	i) Expression of choriogenins (*chgH*, *chgL*, and *chg minor*) was induced in the liver of male medaka (1 µg/L); *dexamethasone-induced-ras-related*	Uchida et al., (2010)

			protein I is also induced.<br>ii) Expression of *vtgI*, *vtg2* and *esr1* was induced in the liver of male medaka exposed to 100 ppb	
32.	Diclofenac (DIC)	<u>EXPOSURE 1</u>:<br>1, 12.5, 19, 25, 50, 100, and 120 mg/L; Juvenile medaka were exposed for 4 days.<br><u>EXPOSURE 2</u>:<br>1µg, and 8 mg/L; Adult male medaka exposed for 12h, 1 day, 2 days and 4 days	Exposure 1:<br>i) Survivability was concentration dependent. LC_{10} was 8 mg/L<br>Exposure 2:<br>i) Expression of vtg, cyp1a, and p53 mRNAs were enhanced in liver, gill, and intestine of male fish exposed either to 1µg/L or 8 mg/L DIC after 96 h exposure	Hong et al., 2007
		0.001, 0.01, 0.1, 1.0, 10.0 mg/l:<br>Fertilized eggs exposed<br>until 84 dph	i) Fertility and hatchability of the embryos tended to decrease in a concentration-dependent manner.<br>ii) GSI in female fish increased in a concentration-dependent manner.	Lee et al., (2011)
		7.1, 37, 78 µg/l; Reproductively adult male and female fish were exposed for 14	i) During exposure, the fecundity and fertility decreased in a	Yokota et al., (2017)

		days and depurated for 14 days.	concentration-dependent manner.<br>ii) Females have swollen abdomen.<br>iii) Some of the males showed defects in the lower jaw.<br>iv) During recovery period, the reproductive and morphological abnormalities recovered, while the defect in lower jaw of males were irreversible.	
		0.608, 2.15, 7.29, 26.5, 94.8 µg/L; embryos were exposed for 98 dph	i) No effect on mortality, hatching, and survivability of the embryos and larvae.<br>ii) No effect on growth and behavior.<br>iii) A concentration-dependent mandibular defects was observed (7.29 µg/L and above).<br>iv) Sex ratio with mandibular defects skewed towards male.	Yokota et al., (2018)
		0.32, 1, 3.2, 32, 100 mg/L;<br>Embryos (1 dpf) were exposed until hatching and observed 0-3 dph	i) Concentration-dependent reduction in hatching and embryo-larval survivability<br>ii) Concentration-dependent inhibition in	Pandelides et al., 2021

			swim bladder inflation in larvae	
33.	Diethylhexylphthalate (DEHP)	500, 1000, 5000 µg/L, 1 dph larvae exposed for 85-110 days	i) No change in sex ratio<br>ii) Testis-ova not observed	Metcalfe et al., (2001)
34.	Diethylstilbestrol (DES):	5 µg/L Transgenic medaka Males, exposed for 21 days.	i) Induction of *vtg1* mRNA in liver	Zeng et al., (2005)
		EXPOSURE 1:<br>1,10,100, 1000 ng/L, fertilized eggs were exposed for 40 days, depurated 30 days.<br>EXPOSURE 2:<br>10, 1000 ng/L, adult fish exposed for 21 days	EXPOSURE 1:<br>i) Hatchability significantly decreased and time of hatching and larval mortality and gross morphological abnormalities significantly increased at 100 and 1000 ng/L.<br>ii) HSI remained unchanged; GSI enhanced in 1000 ng/L group.<br>iii) Sex reversal occurred and sex-reversed male laid eggs.<br>iv) *esr1* mRNA was downregulated in liver of male and female fish in a concentration-dependent manner.<br>v) *vtg1* mRNA in liver was upregulated in female	Lei et al., 2016

			fish in a concentration-dependent manner.<br>vi) *vtg1* mRNA in liver of male fish was downregulated in a concentration-dependent manner.<br>EXPOSURE 2:<br>i) HSI and GSI remained unaltered, no sex reversal.<br>ii) *esr1* mRNA in both male and female fish downregulated in a concentration dependent manner.<br>iii) *vtg1* mRNA in male fish upregulated in 10 and 100 while in females downregulated at 1000 ng/L group.	
35.	5α-dihydrotestosterone (DHT)	500 ng/l, 700 ng/L and1µg/L, transgenic fish, embryos exposed from fertilization (0 dpf) to 16 dpf	i) Skewing of sex ratio towards male is concentration dependent.	Spirhanzlova et al., (2020)
		0.063, 0.26, 1.03, 2.97 µg/L; adult male and female fish exposed for 21 days (FSTRA study using TG 229 guidelines)	i) No effect on mortality, growth (length and weight), HSI, and GSI of both male and female fish	Onishi et al., (2021)

				ii) Secondary sexual features (anal fin papillae) increased in both male and female in a concentration-dependent manner.<br>ii) Liver vtg content in both male and female fish remained unaltered, however, the reproductive activity (fecundity and fertility) impaired.	
36.	Diisobutyl phthalate (DIBP)	35, 184,836 µg/L, 3-month-old fish exposed for 3 weeks		i) Hepatic VTZ reduced in females (184 and 836 µg/L), unaltered in males.<br>ii) Secondary sex characteristics remained unaltered in males and females.<br>iii) Number of total eggs, fertilized eggs and fertility rate remained unaltered	Kawashima et al., (2021)
37.	Endosulfan (EDS)	0.06 µg/L, 0dpf embryos exposed for 7 dph, depurated for 5 months		i) Did not affect survival, hatching time and foraging ability<br>ii) Female biased sex ratio	Teather et al., 2005
		EXPOSURE 1:<br>10, 15, 22.5, 33.75,50.63 µg/L;		i) LC_{50} for 96h exposure was 22.18 µg/L<br>ii) VTG induced in male fish (13.72 µg/L)	Lee et al., (2013)

		adult fish exposed for 96h;<br>EXPOSURE 2: 13.72, and 16.18 µg/L for 96h		
38.	Endrin (END)	12.5, 25, 100,200, ng/L, embryos (4hpf) exposed until 8dpf (stage 38)	i) *gsdf* expression significantly lower in XY embryos exposed to 25 ng/L; other concentrations have no effect on *gsdf* expression either in XY or XX embryos.	Horie et al., (2022a)
39.	Equol (EQ)	0.4, 0.8 µg/L,<br>0 dph larvae exposed for 100 days	i) No difference in sex ratio<br>ii) Concentration-dependent enhancement of testis-ova formation in males<br>ii) The ovary of females is also affected.	Kiparissis et al., (2003a)
		2,8, 40, 200, 1000 ng/L.<br>1 dph transgenic medaka larvae exposed for 100 dph	i) Induces intersex development in a concentration-dependent manner.<br>ii) Reduced plasma 11-KT level in male medaka	Wang et al., 2016
40.	17β-estradiol (E2)	0.01, 0.12, and 1.66 µg/L; exposed for one month following hatch; harvested on day 56 dph	i) Female based sex ratio<br>ii) Reduced fecundity	Nimrod and Benson, (1998)

		Male and female medaka exposed to 5mg/kg E2 through diet for 10 days. Juvenile medaka exposed for 170 days (184 dpf) to 0.05, 0.5 and 5.0 mg/kg E2 via food	i) Male and Female have increased level of serum vitellogenin (adults 10 days exposure) (juveniles 170 days)<br>ii) Female-based sex ratio (juvenile)<br>iii) Kidney abnormalities (juvenile)	Patyna et al., (1999)
		1µg/L; 2-16 dph; depuration 2 months	i) Female based sex ratio	Foran et al., (2000)
		15 µg/L; stage 10 embryos, and 1, 7, and 21 dpf larvae exposed for 6 days; observed after 5 months	i) Sex ratios were significantly biased towards females exposed to E2 in stage 10, and 1, and 7 dph larvae.<br>ii) Intersex gonads are also observed.	Koger et al., (2000)
		1,3, 10, 30, 100 nmol/L; Reproductively active adults exposed through water for 14 days	i) Reduced egg production and egg hatch.	Shioda and Wakabayashi, (2000)
		0.001, 0.01, 0.1, 1 µg/L; Exposed 1-100 dph)	i) At 1.0 µg/L only few fish survived, however, all the survived fish are female: generalized edema, hydropic degeneration in glomerulus of the kidney and	Metcalfe et al., (2001)

			vacuolization of hepatocytes in the liver<br>ii) Sex ratio (0.1µg/L) 65% are female than 35% in female in controls; males have testis ova (15 males)<br>iii) 0.01 µg/L 10% male with testis ova; 0.1µg/L: all male with testis ova; 1µg/L high mortality and only females surviving.	
		0.005, 0.05, and 1ppb [adults exposed for 5 weeks]<br>0.1, 1.0, 25, 50, and 100 ppb [hatched embryos were exposed for 200-230 dpf]	i) Female-specific protein in the serum of male fish was detected.<br>ii) Female-based sex ratio; test-ova observed.	Tabata et al., (2001)
		29.3, 55.7, 116, 227, 463 µg/L; adult medaka exposed for 21 days	i) 96 h LC_{50} for adult medaka is 2.0 mg/L<br>ii) Fecundity and fertility reduced in medaka exposed to 463 µg/L E2.<br>iii) Males developed testis-ova<br>iv) Hepatic VTG enhanced in male fish exposed to 55.7 µg/L and above E2 concentrations.	Kang et al., (2002a)

		0.05, 0.5, 1.0 µg/L; embryos were exposed from 0 dpf until hatching of all eggs in controls, adult males were exposed for 5 weeks.	i) LC_{50} for embryos were 0.46 mg/L (72 h)<br>ii) LC_{50} values (72 h exposure) of adult male and female medaka were 3.5 and 3.5 mg/L<br>iii) IC_{50} for inhibition of egg hatching 0.47 mg/L.<br>iv) Female -specific protein (FSP) were induced in the blood of male medaka that were exposed for 5 weeks to E2 higher than 0.005 µg/L	Kashiwada et al. (2002)
		3 and 30 µg/g through diet for 2 weeks.	i) Fecundities reduced.<br>ii) Male sexual behavior (dancing, floating, crossing) remained unchanged.	Oshima et al., (2003)
		1µg/L, female leukophore free (FLFII) strain, exposure period 2 - 102 dph.	i) Female-based sex ratio<br>ii) Exhibited histological alterations in kidney.	Balch et al (2004b)
		15 ng/mL, 6-7 months old male, exposed for 3 days or 16 days.<br>6 months old male medaka were exposed to 15 ng/L of E2 for 16 days	i) Induction of choriogenins and vitellogenin in the liver exposed 3 days or 16 days.	Hall et al., (2005)

		0.05, 0.1, 1.0 µg/L; Homozygous F3 transgenic male fish; exposed for 21 days.	i) *vtg1* mRNA in liver was induced in a concentration-dependent manner	Zeng et al., (2005)
		1 µg/L; exposed for 1-100 dph	i) Female biased sex ratio as observed from gonad histology	Balch and Metcalfe, (2006)
		150 ng/L; 20-60 dph exposure	i) Female biased sex ratio	Hirai et al., (2006)
		200 ng/L; adult fish exposed for 3 weeks.	i) *esr1* transcripts decreased in testis.	Zhang et al., (2008d)
		(200 ng/L); 5 months of age, 21 days exposure	i) Impaired reproductive capacity (both fertility and fecundity decreased).<br>ii) Hepatosomatic index (HSI) in males significantly increased.<br>iii) Induction of plasma vitellogenin in males and in females (no difference with controls).	Sun et al., (2009)
		5 and 50 ng/L.<br>0 dph, 1 month old and 4 months old-7dph; 1 month old, exposed for 7 days; photoperiod and temperature cycle: 10L14D-10°, 12L12D- 20°, 10L14D-30°	i) The *vtg1* mRNA level in larval medaka increased when exposed to high concentration of E2 (50 ng/L) both in 20°C-12L12D and 30 °C14L10D.<br>ii) The degree of increase of *vtg1* mRNA in male and female juveniles	Jin et al. (2011a)

			depend on photoperiod-temperature regime.<br>iii) Induction of *vtg2* in males and females (observed only 50 ng/L E2) in all temp/photoperiod regime.<br>iv) *esr1* remained unaltered in males, but in female juveniles' significant induction was observed only in 20°C-12L.	
		10, 30, 100, 300 ng/L); male medaka (5-6 months old) exposed for 24, 48 and 72 h	i) E2 (concentration dependent; after 48 h increased the precursor of egg protein vitellogenin (*vtg1* and *vtg2*) and precursors of egg envelop subunit proteins choriogenins (*chgH* and *chgL*) in the liver of male fish)<br>ii) The minimum effective dose of E2 in the liver of male medaka (48 h exposure; 5-6 months old) is 10 ng/L for *chgH* and *chgL*, and 30 ng/L for *vtg1* and *vtg2*	Kamata et al., (2011)
		800 ng/L, medaka adults, 3 weeks	i) Testis ova are induced after 3weeks exposure.	Hirakawa et al., (2012)

			ii) Upregulation of genes related to zona pellucida (Microarray analysis) and oocyte marker gene *42Sp50* in testis.	
		0.001,0.01,0.1 and 1 ng/mL,<br>transgenic medaka embryos exposed from 5hpf until hatching	i) Expression of *GnRH1*, *GnRH2*, *GnRH3* mRNAs were down regulated in embryos exposed to 1 ng/mL E2<br>ii) *esr1* expression was upregulated (1 ng/mL).<br>iii) Expression of *GnRH-R1*, *GnRH-R2*, *GnRH R3* and *esr2a* and *esr2b* mRNAs remained unaltered.<br>iv) Other parameters such as heart rate, eye pigmentation, head growth, and time to hatch was affected.	Lee et al, (2012)
		1, 10, 100, 1000 ng/L (β-estradiol-17 valerate);<br>Exposed 0-70 dpf	i) Female biased sex ratio<br>ii) Adverse effects in hatchability and hatching time (10 ng/L and above)<br>iii) HSI increased in males and females<br>iv) GSI increased in males and decreased in females.<br>v) Sex reversal occurred.	Lei et al., (2013)

			vi) *erα* and *vtg1* mRNAs in the liver of females are down- regulated; upregulation of *vtg1* mRNA in the liver of male fish.	
		50ng/L.<br>0-98 dpf exposure	i) Female based sex ratio	Green et al., (2015)
		In vitro reporter gene assay; gene transcriptional activities for E2	i) ESR1: EC_{50}= 1.31X10 $^{-10}$ M<br>ii) ESR2a: EC_{50}= 3.25X 10^{-11}M<br>iii) ESR2b: EC50= 8.16X 10^{-11}M	Tohama et al., (2015)
		1 ng/mL, transgenic medaka embryos and larvae; exposure period 1 dpf-20 dpf	i) Advances eye pigmentation<br>ii) No hatching delays.<br>iii) Decreased locomotor activity.<br>iv) Diameter of GnRH3 neurons decreased	Inagaki et al., (2016)
		0.92, 2.8, 8.9, 27.9, 84.3 ng/L adult fish (F0) were exposed for 4 weeks, F1, 15 weeks, F2, 15 weeks (total 29 weeks) MEOGRT protocol	i) Fecundity and fertility significantly reduced (LOEC was 28 ng/L)<br>ii) No effect on hatching (LOEC <84 ng/L)<br>iii) Vtg enhanced significantly (LOEC 28 ng/L)<br>v) Secondary sexual features reduced (LOEC was 28 ng/L)	Flynn et al., (2017)

		10 and 100 ng/L; Exposure protocol TG240	i) Female biased sex ratio	Lee et al (2017a)
		2 ng and 50 ng/L, embryos 4hpf- until hatching (10 dpf), evaluated on 112 dpf; larvae, 1 dph-30 dph; juvenile (30 dph) exposure period 30-80 dph; adult fish exposure period 14 days.	i) No effect on hatching and growth<br>ii) Sex reversal (female-based)<br>iii) No effect on fecundity	Lee Pow et al (2017)
		100 ng/L; 16±2 weeks of age; 21 days exposure [TG 229]	i) Fecundity reduced only after 21 days exposure.<br>ii) Secondary sexual features in males (number of papillary processes in the fin rays) remained unaltered.<br>iii) *vtg1* and *vtg2* genes in the liver of male and female medaka increased after 7-, 14-, and 21-days exposure.<br>iv) Hepatic vitellogenin protein in male liver significantly increased in 7, 14, and 21st day observations.	Kang et al., (2019)

		0.57-36.8 µM, 1dph for 96h;<br><br>2 µM, 5hpf-9 dpf;<br><br>0.1-222 nM,<br>5hpf embryos exposed for 7 and 9 dpf and 0- 3 dph (12 dpf)	i) Hatchability and time to hatching decreased in a concentration-dependent manner.<br>ii) 96h LC_{50} in larvae is 9.4 µM; 14 days LC_{50} for embryos 3.4µM.<br>iii) Upregulation of *chgL* and *chgH* in embryos exposed to 2 mM E2 from 3-9 dpf embryos and 9 dpf larvae, not in 1 and 2 dpf embryos.<br>iv) *chgHm* and *vtg1* mRNA did not respond to 2 µM E2 (1-8 dpf) but increased in 9 dpf larvae; no effect on *vtg2*, *cyp19a1b, esr1*, *esr2a*, *esr2b transcripts*.<br>v) *chgL*, *chgH*, and *chgHm* mRNAs (5 hpf to 7 dpf exposure) responded to E2 in a concentration-dependent manner; no effects on *vtgs*, *cyp19a1b* and, *esr1*, *esr2a*, *esr2b* transcripts.<br>vi) *chgL*, *chgH*, *chgHm*, *vtg1*, *vtg2* and *cyp19a1b* mRNAs (exposure 5 hpf to 9 dpf) upregulated in	Ishibashi et al., (2020)

			a concentration-dependent manner.<br>vii) *chgL*, *chgh*, *chgHm*, *vtg1*, and *vtg2*, *cyp19a1b, and erα* in 12 dpf (0 dph-3 dph) upregulated in a concentration-dependent and developmental-stage- specific manner.; *esr2a* remained unresponsive.	
		500, 700, and 1000 ng/mL; transgenic medaka, 0-16 dpf exposure	i) Female based sex-ratio	Spirhanzlova et al., (2020)
		22.1, 115, 553 ng/L; reproductively adult male and female medaka exposed for 21 days (TG229 protocol)	i) Survivability of the fish affected with the increase in the concentration of E2<br>ii) Reproductive activity (fecundity and fertility) impaired with the increase in the concentration of E2 in the medium.<br>ii) No alteration in the growth of the fish (length and weight)<br>iii) No effect on HSI and GSI of both male and female fish	Onishi et al., (2021)

			iv) Liver vtg content in male and female fish enhanced in a concentration-dependent manner.<br>No change in secondary sex character (papillae in the anal fin)	
		1,10,32,320, 1000 µg/L; embryos 1 dpf were exposed until hatching and observed 0-3 dph	i) Inconsistent effect on hatchability<br>ii) Swim bladder inflation was significantly reduced by E2 exposure in a concentration-dependent manner	Pandelides et al., (2021)
		6.2,12.5,25, 50, 100ng/L; 4hpf to 8 dpf (Stage 38)	i) LOEC for induction of intersex is 23.8 ng/L.<br>ii) No significant difference in *gsdf* gene expression in XY embryos after 17β-estradiol exposure until stage 38 of development (8dpf)	Horie et al., (2022a)
41.	Estriol (E3)	0.01, 0.1, 1, 10 µg/L, exposure period 1-90 dph	i) Higher proportion of males were observed in 0.01 and 1.0 µg/g group.<br>ii) Testis-ova found in males.<br>iii) Eosinophilic fluid observed in body cavities in highest E3 concentration.	Metcalfe et al., (2001)

			iv) LOEL is 0.75 µg/L and NOEL is 0.075 µg/L	
		0. 5, 1, and 10.0 µg/L, transgenic medaka exposed for 21 days.	i) *vtg1* mRNA induced in concentration-dependent manner in males.	Zeng et al., (2005)
42.	Estrone (E1)	0.01, 0.1, 1, 10 µg/L, exposure period 1-90 dph	i) Kidney being the most affected organ in 10 µg/L group<br>ii) Majority of the group from 1-10 µg/L is female.<br>iii) Testis ova are found in all males survived (1-10 µg/L)	Metcalfe et al., (2001)
		6.25,12.5,25.0, 50.0, and 100 ng/Lng/L, exposed for 27 weeks for 3 generations.	i) No effect on reproduction was observed in first generation<br>v) Decline in egg production and fertility in the second generation exposed to 91.4 ng/L<br>vi) Histopathological abnormalities observed in 3rd generation exposed to 47.1 and 91.4 ng/L<br>vii) No effect on HSI of both male and females in first and second generation; no effect on the number of anal fin	Nakamura et al., (2014)

			papillae in the first and second generation males; females developed no papillae.<br>viii) Testis-ova appear in the second and third generation.<br>ix) Liver vitellogenin concentration increased in a concentration-dependent manner in first and second generation in males and only in second generation in females.	
		29,112, 272, 1009 ng/L, 16±2 weeks medaka were exposed for three weeks (TG 229)	i) Hepatic vtz enhanced in males and females in a concentration-dependent manner.<br>ii) HSI enhanced in male in at 272 and 1009 ng/L concentration; GSI decreased in male at 1009 ng/L concentrations.<br>iv) Secondary sex characters remained unaltered in males and females.<br>v) Number of fertilized eggs, total eggs and fertility rate unaltered.	Kawashima et al., (2022)

43.	17α-ethinylestradiol (EE2)	1, 10, and 100 ng/L, exposed 60 days, depuration 6 weeks.	iii) All genetic males (XY) sex-reversed to functional females at 100 ng/L group (immature ovary with different stages of ovarian maturity). No sex reversal at lower concentrations<br>iv) Reduced egg production in females (10 ng/L)<br>v) Aromatase expressed in testis of XY males exposed to 10 ng/L	Scholz and Gutzeit (2000)
		0.0001, 0.001, 0.01, 0.1, 1 µg/L, 1dph exposed for 100 days.	i) Toxic at 1.0 µg/L<br>ii) Female-based sex ratio<br>iii) Eosinophilic fluids observed in organs and body cavity (0.1-1.0 µg/L)<br>iv) Testis-ova observed in males	Metcalfe et al., (2001)
		Larval exposure: 0.2, 5, 500, and 2000 ng/L; 2 dph larvae were exposed for 2 weeks.<br>Adults exposed in larval stage (0.2, 5, 500 and 2000) were reexposed to 0.8, 20, 2000, and 8000 ng/L for 2 weeks.	i) Hatchling exposure produced no changes in VTG content in the liver, no change in circulating steroids, no change in reproductive activity<br>ii) Reexposure of the adults inhibited reproduction, increased hepatic VTG and ER	Foran et al., (2002)

		In ovo exposure: 0.2, 5, 500, and 200 ng/L; parents (breeders) were exposed for 24h and the eggs were collected within 2 h of light turned on and grown to adults. Half of the animals reexposed to 0.8,20, 2000, and 8000 ng/L	content and increased E2 concentration in males.<br>iii) Parental exposure produces permanent change in in hepatic ER and VTG in adults	
		2.5, 25, and 100 ng/L, male medaka exposed for 7 days	i) The LOEC for *vtg* mRNA induction in liver of male medaka are 25 ng/L	Islinger et al., (2002)
		10, 20, 50, 100, 200 µg/L, mature male medaka exposed for 6 days	i) *chgL* mRNA in liver was induced in 10-200 µg/L in a concentration-dependent manner<br>v) *chgH* mRNA expression was observed in 20 µg/L and above	Lee et al., (2002)
		32.6, 63.9, 116, 261, 488 ng/L, mature medaka for 21 days.	i) Decrease in fecundity (488 ng/L)<br>ii) Males with testis ova (63.9-488 ng/L) (still fertile)<br>iii) Reduced egg production and high mortality (488 ng/L)	Seki et al., (2002)

			iv) Hepatic vitellogenin concentration in males increased (63.9 ng/L)<br>vi) Reproductive based LOEC is 488 ng/L; hepatic vtg and testis-ova based LOEC is 63.9 ng/L	
		0.2, 2.0, 10 ng/L, exposure period 2-5 dph -until mature (4 and 6 months of age)	i) Reproductive behavior was suppressed with in both male and female fish.<br>ii) Male fish with testis ova (gonadal intersex) are still capable of reproductive behavior and could fertilize eggs.	Balch et al., (2004a)
		15.6, 31.3, 62.5, 125, 250 ng/L; adult male and female fish exposed for 21 days	i) A duration and concentration-dependent enhancement in the liver vtg contents occurred in males and females	Nozaka et al., (2004)
		0.02, 0.2, 2, 20, and 40 µg/g diet; adult fish fed for 7 days.	i) The plasma vtg content of both male and female fish enhanced in a dose-dependent manner	Chikae et al., (2004)
		0.1, 0.5, 2.5, 5, ng/ egg of *olvas*-GFP/ST-II YI medaka line; evaluated 10 dph for fluorescence; the fluorescence negative	i) Showed general toxicity, no effect on total length or body weight.<br>ii) Germ cell prevention was prevented in XX females.<br>iii) Complete sex reversal.	Hano et al., (2005)

		fish were evaluated on 100 dph for gonad histology		
		0.05, 0.1 and 1 µg/L, transgenic homozygous male fish, exposed for 21 days	i) vtg1 mRNA was increased in a concentration-dependent manner	Zeng et al., (2005)
		10 and100 ng/L, exposure period 1-60 dph	i) Sex reversal concentration-dependent<br>ii) Female-based sex ratio<br>iii) VTG protein in whole body was enhanced in 100 ng/L	Orn et al., (2006).
		5,50, 500 ng/L, 4-month-old exposed for 7 days.	i) Down regulation of male brain *gnRH R1* and *arα* and testicular *cyp17*mRNA expression<br>ii) Suppression of male sexual behavior<br>iii) Up regulation of *cyp19b* in male brain<br>iv) Enhancement of HSI and up regulation of *erα, vtg I, vtg II, chgH, chgHm, chgL* in liver of male fish.<br>v) Down regulation of *cyp3* and *annexin max2* in the liver of male fish<br>vi) Down regulation of *cyp19a* in the ovary of female fish	Zhang et al., (2008c)

		60, 120, 240, 480 ng/L, adult male exposed for 3 weeks.	i) Vtg1 and vtg2 levels in serum is increased in male fish (60 ng/L and above)<br>ii) Velocity of sperm was found to be increased after 3weeks of exposure at 60-480 ng/L.<br>iii) Inhibitory effects were observed in fertilization rate, hatchability (highest inhibition was at 60 ng/L)	Hashimoto et al., (2009)
		5, 50, and 500 ng/L, adult males and females exposed for 7 days.	i) No change in HSI and GSI.<br>ii) EE2 at 500 ng/L reduced fecundity and altered gonad histology: induced *vtg2* expression in male liver and gonad (not in females).<br>iii) EE2 at less than 50 ng/L *cyp19a* expression in early-stage oocytes was greater in females (reversed in 500 ng/L)	Park et al., (2009)
		20 ng/L, adult fish exposed for 72 h	Brain:<br>i) In male brain only *esr1* and *cyp19b* upregulated, no change in *esr2a* and *arα*	Sun et al., (2011a)

			ii) In female brain, except *arα*, both *esr1* and *esr2*a were down regulated. No alteration in *cyp19b*<br>Liver:<br>i) Enhanced hepatic *vtg1* and *vtg2* mRNA transcripts in male medaka, however, *esr1*, *esr2a*, and *arα* remained unaltered.<br>ii) In females, except *esr1*, other genes (*esr2a*, *arα* and *vtg 1* and *vtg2* remained unaltered.<br>Testis:<br>i) In testis *cyp11a* is down regulated after EE2 exposure.<br>ii) EE2 exposure (10, 100 ng/L) 1-60 dpf; histological observation showed female biased sex ratio	
		100 ng/L, adult males exposed for 3-5 weeks.<br>20 ng/L, adult males exposed for 1-6 weeks.	i) Occurrence of ova in the testis (testis-ova)<br>ii) *vtg1*, *chgH*, *chgHm*, *chgL* are highly expressed in liver of 100 ng/L EE2; *cyp1c1* also induced in liver; apolipoprotein L1 and	Hirakawa et al., (2012)

			heart type fatty acid binding protein reduced.<br>iii) Upregulation of zona pelucida related proteins and oocyte marker gene *42Sp50* in testis<br>iv) *Zpc5* gene in testis could be used as a marker gene for testis-ova induction in testis.	
		1µg/L, stage 10 embryos (FLFII strain) exposed for 42 days.	i) Did not show any significant alteration in embryonic hatchability or abnormalities in gross development.<br>ii) Secondary sexual features altered in both males and females.<br>iii) Females showed completely mature oocytes in gonads; however, males are feminized (100% males with ovaries).<br>iv) Hepatic CYP3A activity remained unaltered in both males and females	Liao et al., (2014)
		1.1 mg/L, embryos exposed for 7 days.	i) EC_{50} is 1.7 mg/L<br>ii) Induced the expression of *cyp19a1b*, *esr2*, *vtg1* and isprenoid and cholesterol synthesizing	Schiller et al., (2014)

			gene *lss*, *sc4mol* and *mvd*<br>iv) Did not induce *esr1* gene.<br>v) Androgen receptor (*ar*) was down regulated	
		5 ng/L, 15 dph larvae exposed for 10 days	i) No change in ovarian structure protein (*osp1*) expression in male larvae<br>ii) *vtg* did not show any significant changes in male larvae	Abdel-Moneim et al., (2015)
		0.05µg/L, exposure period 8hpf-7dpf	i) No apparent phenotypic abnormalities in F0 and F1 generation.<br>ii) Significant reduction in fertilization rate in F2.<br>iii) Reduction of embryo survival in F3.	Bhandari et al., (2015)
		0.01 µg/L, exposure period 8hpf- 50 dph.	i) Transcriptional profiling revealed 1475 differentially expressed genes (DEG) in testis.<br>ii) Gene ontology (GO) analysis showed significant enrichment in cilium organization, microtubule-based process and organelle assembly.<br>iii) Pathway analysis showed significant	Bhandari et al., (2020)

			enhancement on integrin signaling pathway, cadherin signaling pathway, Alzheimer disease-presenilin pathway.<br>iv) Single nucleotide polymorphism (SNP) and insertion-deletion (Indel) analysis found no significant difference in mutation rates.	
		100 µg/L, F0 fish exposed 8hpf-7dpf.	i) mRNA expression of kisspeptin 1 (*kiss1*) and gonadotropin releasing hormone 1(*gnrh1*) in the brain of male fish was enhanced in F0 fish, while gnrh receptor 2 (*gnrhr2*) was reduced. Expression of *kiss1* and *kiss2* and *kiss1r* and *kiss2r* mRNAs enhanced in F2, while *gnrh1* mRNA was reduced.<br>ii) The mRNA expression pattern in *fshβ* and *lhβ* in the pituitary of male fish was enhanced and *fshr* was reduced in the testis of F0 males, F2 males, only the *lhβ*	Thayil et al., (2020)

			mRNA in the pituitary was enhanced.<br>iii) The steroid acute regulatory protein (*StAR*), *cyp19a1a*, *erα* and *arα* expression remained unaltered in F0, while in F2 only *star* was upregulated and *erα* was down regulated.<br>iv) Expression of *dnmt3bb* mRNA in brain of male fish was decreased in F0 and increased in F2; in testis, *dnmt3aa* was enhanced in F0, while in *dnmt1* and *dnmt3bb* were enhanced in F2.<br>v) Global DNA methylation pattern in testis showed significant reduction in both F0 and F2 generations.	
		17.8, 84.9, 424 ng/L; reproductively active adult male and female fish exposed for 21 days (FSTRA using TG229 protocol)	i) Although concentration-dependent effect on survivability of the fish was observed, no effect on growth (length and weight) and the HSI and GSI and secondary sexual characteristic features of both male	Onishi et al., (2021)

			and female fish was observed.<br>ii) Reproductive activity (fecundity and fertility) was impaired in a concentration-dependent manner<br>iv) Liver vtg content was enhanced in both male and female fish in a concentration-dependent manner.	
		1,10, 32, 320, 1000 µg/L; embryos 1 dpf were exposed until hatching	i) Concentration-dependent reduction in swim bladder inflation.	Pandelides et al., (2021)
		6.2,12.5,25, 50,100 µg/L, 4hpf-8dpf (stage 38)	i) LOEC for induction of intersex is 24.5 ng/L<br>ii) *gsdf* gene expression in XY embryos remained unaltered.	Horie et al., (2022a)
		10, 100, 1000, 10000 ng/L,<br>Posthatch larvae 0, 5, 10, 30, 70 dph and adults,<br>T4 and antithyroid compounds like TU, PFOA, TBBPA were used together with EE2	i) Concentration dependent enhancement (100- 10,000 ng/L) of *vtg* and *chgH* mRNAs occurred in males during development (5-70 dph); coexposure with T4 potentiated the effects of EE2.<br>ii) Antithyroid compounds like thiourea (TU), perfluorooctanoic acid	Myosho et al (2022)

			(PFOA), and tetrabromobisphenol A (TBBPA) suppressed EE2 (1000 ng/L)-induced *vtg* and *chgH* gene expression.<br>iii) Enhancement of *trα*, *esr1* and *esr2b* mRNAs occurred by EE2 (1000 ng/L); TU, TBBPA, and PFOA suppressed the expression of EE2-induced *trα*, *esr1*, and *esr2b* genes and that suppression was rescued by T4.	
		1, 10, 100 µg/L; Fertilized eggs were exposed until 1 d before hatching (8 dpf; stage 39); The hatched larvae were evaluated on 0 dph, 30 dph, and mature fish	i) A concentration-dependent enhancement in sex reversal (feminization) occurred in XY fish (XY female) not in XX fish.	Watanabe et al., (2023)
44	2-ethylhexyl 4-hydroxybenzoate (2-EHHB)	6.25, 12.5. 25, 50, 100 µg/L; MEOGRT protocol (multigeneralional total 32 weeks; F0 adults 3 weeks; F1; 15 weeks; F2 15 weeks)	i) Fecundity decreased<br>ii) Fertility rate diminished<br>iii) Inhibition of growth<br>iv) Delayed reproductive tract development in F1 subadults males<br>v) Masculinization of the renal phenotype in F1	Matten et al., 2023

			adult females (renal tubular eosinophilia)<br>vi) Reduced hepatic energy storage (liver glycogen vacuoles) in F1 and F2 males and females<br>vii) The *vtg1* in liver of male F1 fish significantly enhanced in a concentration-dependent manner. while in F2 males, liver *vtg1* mRNA reduced<br>viii) Decrease in anal fin papillae in F2 adult males	
45.	2-ethylhexyl 4-methoxycinnamate (EHMC)	0.05, 0.158, 0.5, 1.58, 5 mg/L;<br>Embryos (24hpf) were exposed until 5 months (F0) and the F1 fish exposed until 38 days	ix) After > 3 months of exposure, significant decrease in reproductive performance<br>x) No change in *esr1* and *arα* mRNAs in 17 and 38 dpf of F0 and on 38 dpf of F1 fish<br>xi) No change in plasma E2 concentration in both male and female adult fish<br>xii) In liver no change in mRNA expression in *esr1, arα*, and *vtg1*.	Lee et al., (2019b)

			xiii) Thyroid hormones (T3 and T4) decreased in F1 fish at 38 dpf<br>xiv) Downregulation of *dio2* and upregulation of *trh* in both F0 and F1 generations.<br>xv) Upregulation of *trα*, *trβ* in male adult fish	
46.	Fadrozole (FAD)	0.05,0.25, 0.5,1, 10 mg/g of food; 0 dph fries fed 90 dph	xvi) Suppressed the ovarian cavity formation from 30 dph.<br>xvii) Did not affect early oogenesis and folliculogenesis during ovarian development.<br>xviii) Spermatogenesis (Testis-ova) observed in some part of the ovaries (10 mg/g food)	Suzuki et al., 2004
		10, 50, and 100µg/L,<br><br>fries exposed for 2 weeks, assayed on 5-, 9-, and 14 days after exposure	i) No change in adult sex ratio<br>ii) Aromatase enzyme activity reduced at 100µg/L concentration after 14 days exposure.	Kuhl and Brouwer, 2006
		800 µg/g of dried food,<br>1dph (fry) larvae exposed for 6 months	i) Milt volume, initial sperm mortality, maximum sperm swimming duration, and sperm morphology did not alter.	Thresher et al., (2011)

			ii) Fertilization rates unaffected; hatching reduced by 31%<br>iii) Partial masculinization	
		1,10, 100 µg/L; adult fish exposed for 7 days	i) Upregulation of *cyp19a* in the ovary in a concentration-dependent manner	Park et al., (2008)
		50 µg/L, Adults exposed for 7 days.	i) Reduced fecundity<br>ii) FAD after 8h exposure downregulated *esr1* and *chgL* expression in the liver of females	Zhang et al., (2008b)
47.	Fenitrothion (FNT)	100, 300,1000, and 3000 µg/L, larvae (35 dpf) exposed for 28 days	i) The number of papillary processes was significantly decreased in a dose-dependent manner in XY medaka.<br>ii) No effect on total length and body weight (except 1000 and 3000 µg/L where total length and weight significantly reduced)<br>iii) No effect of the vtg concentration in the liver of male medaka; whereas significant increase in females (100-1000; no data in 3000 µg/L)	Horie et al., (2017).
		375,750,1500,3000 µg/L, embryos (4hpf)	i) *gsdf* expression in XY males exposed to	Horie et al., (2022a)

		exposed until 8dpf (stage 38)	fenitrothion are not significantly different from control XY male embryos (stage 38)	
48.	Fenoxycarb (FNC)	0.001,0.01,0.1, 1, 5 mg/L, in presence or absence of testosterone (28.57 mg/L),<br>0 dpf fries exposed for 24h and 96 h	i) 5 mg/L is lethal to the fries.<br>ii) No change in estrogen, androgen and thyroid signaling with or without testosterone	Spirhanzlova et al., (2017)
49.	Fenvalerate (FV)	(0.0619, 0.294, 1.30 µg/L, 16±2 weeks old male and females were exposed for three weeks	i) Hepatic VTG significantly decreased in males, no alteration in females.<br>ii) Secondary sex characteristics unaltered in males and females<br>iii) The number of total eggs, fertilized eggs, and fertility rate remained unaltered.	Kawashima et al., (2022)
50.	Fipronil (FPN)	3,10, 30 µg/L; newly hatched larvae were exposed until 28 dph.	i) Inhibited growth in both sexes.<br>ii) Down regulation of *fshr* and *lhr* in females, no alteration in males<br>iii) mRNA levels of *StAR*, *cyp17a*, and *cyp19b* increased in males.<br>iv) mRNA levels of *20β-hsd*, *cyp19a*, *cyp19b*	Sun et al., (2014)

			was significantly increased in females.<br>v) Upregulation of *vtg1* and *vtg2* mRNAs were observed in both sexes<br>vi) No alteration in *esr1* and *arα* mRNAs occurred in both sexes.	
		0.1, 1, 10, 100, 200, 400, 600, 800, and 1000 µg/L, 6hpf embryos exposed for 14 days (until hatching)	i) No embryo mortality.<br>ii) Concentration dependent decrease in hatching success.<br>iii) Tail curvature increased in a concentration-dependent manner (ED50= 668 µg/L).<br>iv) Impaired larval swimming (200 µg/L and above).	Wagner et al., (2017)
51.	Fluoxetine (FLX)	0.1, 0.5, 1, and 5 µg/L, adult fish exposed for 4 weeks	i) No significant change in egg production, fertilization rate and spawning or hatching.<br>ii) No incidence of developmental abnormality was observed.<br>iii) Gonadosomatic index, hepatic vitellogenin, and ex vivo gonadal steroidogenesis were also unaffected.	Foran et al., (2004)

			iv) Estradiol in plasma significantly increased in females.<br>v) Testosterone in plasma of male and females remained unaltered.	
52.	Fluridone (FLR)	A: EMBRYO EXPOSURE:<br>0.03, 0.5, 1,2, and 4 mg/L; fertilized eggs were exposed until 14dpf.<br>B: LARVAL EXPOSURE:<br>0.08, 0.8, 4.2, 21 and 104 mg/L;<br>7dpf larvae exposed for 6h.	i) Hatching was reduced in a concentration-dependent manner<br>ii) Lead to abnormal swimming behavior in larval medaka<br>iii) Transcriptome analysis identified a total of 799 differentially expressed genes (555 upregulated and 244 downregulated) in males.<br>iv) Gene set enrichment analysis identified AcHE, retinoic acid receptor, insulin receptor substrate, glutathione reductase, and glutathione-S-transferase, exhibited concentration and sex-dependent response.	Jin et al., (2020)
53.	Flutamide (FLU)	0.02, 0.2, 2, and 20 mg/g diet; adult fish fed for 7 days.	i) Increased the plasma *vtg* levels in females in a concentration-dependent manner.	Chikae et al., (2004)

			ii) Males remained unresponsive.	
		93.8, 188, 375, 750, 1500 µg/L, adult males and females exposed for 21 days.	i) No enhancement in vtz concentration in the liver of male and female fish.	Nozaka, (2004).
		0.101,0.202, 0.397, and 1.56 mg/L, adults exposed for 3 weeks	i) Fecundity and fertility were significantly decreased at 1.56 mg/L concentration.<br>ii) Testis-ova observed in 0.202, 0.397, and 0.787 mg/L groups.<br>iii) Vitellogenin remained unaltered (in both males and females).	Kang et al., (2006)
		0.032, 0.32, 1, and 3.2 mg/L; 7 dph larvae exposed for 96h depurated three months (90 days) until the development of sexual maturity	i) 96 h LC50 for males was 1.92 mg/L; for females no significant mortality was observed.<br>ii) Concentration-dependent reduction in the length and weight of males but not in females.<br>iii) The LOEC for induction of testis-ova in males is 0.32 mg/L.<br>iv) Formation of testis-ova, disruption of spermatogenesis, and ovarian cell necrosis.	Leon et al., (2007; 2008)

			v) Hypertrophy of thyroid follicular cells in males.<br>vi) Microarray analysis indicated upregulation of 75 and 853 genes in males and females, respectively related to sex differentiation and growth; however, 260 genes in males and 962 genes in females are downregulated.	
		125, 250, 500, 1000 µg/L<br><br>EXPOSURE 1: Adults (98 dpf) were exposed for 21 days.<br><br>EXPOSURE 2: Juveniles (40 dpf) exposed for 28 days	i) No significant effect on fertility either in adult or juvenile test.<br>ii) No significant effect on the number of male papillary processes in adults; however, in juveniles, at 1000 µg/L concentration. decrease in male papillary processes were observed.<br>iii) No female samples develop papillary processes either in adult of juvenile test.	Nakamura et al., (2014)
		6.2 mg/L, embryos (3-4 hpf) exposed for 7 days	iv) EC50 is 6.93 mg/L.<br>v) Reduced the expression of *gnrhr2*, *cyp11b* and *3βhsd*.	Schiller et al., (2014)

			vi) Repressed *esr2a* and *cyp19a1b*	
		119, 248, 497, 925 µg/L; reproductively active adult male and female fish were exposed for 21 days using FSTRA protocol following TG 229 guidelines.	i) No change was observed on mortality, growth (length and weight), secondary sexual features, HSI, GSI, liver VTG content as well as reproductive activities (fertility and fecundity) of both male and female fish.	Onishi et al., (2021)
		125,250, 500,1000 µg/L, embryos (4hpf) exposed until 8 dpf (stage 38)	i) No significant induction of *gsdf* expression in XY embryos	Horie et al., (2022a)
54.	Genistein (GEN)	25,50,100, 250, and 500 µg/g food, adult females exposed for 15 days	i) ED_{50} for the induction of papillary processes in the anal fin is 37µg/g food.<br>ii) ED_{50} for reversal of genotypic females to phenotypic males (androtermonic) is 110 µg/g food.	Hishida and Kawamoto, (1970)
		1,10,100, 1000 µg/L,<br><br>0dph larvae exposed for 100 days.	i) No difference in sex ratio<br>ii) Testis-ova seen in 12% males exposed to 1000 µg/L.<br>iii) Both Spermatogenesis and oogenesis affected	Kiparissis et al., (2003a)

		6 and 10 mg/L,<br>1-2 hpf embryos exposed for 7 days	i) Morphological deformities (20% of the embryos) such as reduced blood circulation, delayed blood vessel development, and malformation of notochord, tail and head were observed.<br>ii) *Cyp19a1b, vtg1, esr2α, pax2*, *dio2* are upregulated after 10 mg/L exposure.<br>iii) *Cyp19a1a*, lss, and *tp 53* are down regulated by 10 mg/L.<br>iv) *dio2* are down regulated by 6 mg/L.<br>v) Expression of *ar,3β-hsd, cyp11b*, and *gnrhr2* remained unaltered.	Schiller et al., (2013, 2014)
55.	Glyphosate (GLP)	0.5 mg/L, embryos (8hpf) exposed until 15 dpf, depurated until 100 dpf	i) Decreased hatching, increased developmental abnormalities<br>ii) Fecundity and fertilization efficiency did not alter in sexually mature fish<br>iii) *tet1* and *tet3* mRNAs increased while *dnmt3aa* and *dnmt1*	Smith et al., (2019)

			mRNAs decreased in 15 days embryos (fries)<br>iv) In testis, not in ovaries, expression of *fshr* and *arα, dnmt1, and dmrt1* significantly reduced.	
56.	Graphene oxide (GO)	EXPOSURE 1:<br>25,50,100, 200 µg/g, adults exposed through intraperitoneal injection, depurated 21 days.<br><br>EXPOSURE 2:<br>20 mg/L<br>Adult fish exposed for 4 days depurated for 21 days	EXPOSURE 1<br>i) LD_{50} found to be sex dependent.<br>ii) Fecundity unaltered; hatchability reduced (200µg/L).<br>iii) Histopathological alteration did not affect spermatogenesis or oogenesis.<br>iv) Morphology of the granulosa cells in the ovary or Leydig cells and Sertoli cells in the testis remained unaltered.<br>v) Accumulation of proteinaceous fluid in the hepatic vessels and hyperplasia in interstitial lymphoid cells in the kidney<br>vi) Inconsistent effects on interrenal glands (100µg/g)<br>vii) Inconsistent effects on pancreatic δ-cells.	Dasmahapatra et al., (2020a, b); Dasmahapatra and Tchounwou (2022a, b; 2023a, b)

			EXPOSURE 2:<br>viii) No mortality<br>ix) Inconsistent effects on interrenal glands<br>x) Inconsistent effects on pancreatic δ-cells	
		2.5, 5, 10, 20 mg/L, 1dpf larvae exposed for 4 days, depurated until 47 dph	i) Sex ratio remained unchanged.<br>ii) ovo-testis seen<br>iii) Gonad (testis or ovary) remained unaltered.<br>iv) Minimal effects on the histology of liver and kidney.<br>v) Nonlinear effects observed on the size of the thyroid follicles.<br>vi) Inconsistent effects on interrenal glands.	Myla et al. (2021a, b), Asala et al. (2021, 2022), Dasmahapatra and Tchounwou (2022a, 2022b)
57.	Ibuprofen (IBP)	1,10, 100 µg/L, adult fish exposed for 6 weeks	i) Increased the number of eggs but decreased the spawning events in a concentration-dependent manner.<br>ii) HSI of females tended to increase while HSI of males tended to decrease.<br>iii) Cyclooxygenase activity tended to decrease in females and increased in males.	Flippin et al., (2007)

			iv) No pathological damages in gill, liver, and head kidney.	
		0.01,0.1,1, 10, 100, 1000 µg/L, fertilized eggs exposed for 12 days (TG 210), depurated until 132 dph	i) NOEC was 0.0001 mg/L.<br>ii) Adult survivability reduced significantly in a concentration-dependent manner.<br>iii) Induction of VTG in male fish blood (1000 µg/L)<br>iv) Fewer broods per pair, and more eggs per brood (10 and 100 µg/L).<br>v) HSI and GSI are not affected except females exposed to 0.1 µg/L (not concentration dependent).<br>vi) No histological lesion in liver, gonads, and kidney.<br>vii) Prenatal exposure as low as 0.01 mg/L delayed hatching of eggs.	Han et al., (2010)
58.	Kanechlor 400 (KC-400)	1µg/g body weight. Both male and female adult fish fed for three weeks	i) The expression of *chgL*,*chgHm* and *arα* in the liver of both male and female fish were downregulated.	Nakayama et al., (2011)

			ii) The *vtg1* in liver of males was downregulated while in females upregulated.	
59.	Ketoconazole (KTC)	3,30, and 300 µg/L; adult fish exposed for 7 days.	i) Reduced fecundity is concentration and time-dependent manner.<br>ii) Down regulation of *esr1*, *vtgI*, *vtgII*, *cghL*, *chgH*, *chgHm*, and upregulation of *arα, annexin max2* mRNAs in liver of females<br>iii) In males *esr1* and *arα* mRNAs were upregulated while *chgH* and *chgHm* were downregulated in liver.<br>iv) In ovaries downregulation of *esr2*, *arα, lhr, hmgr, StAR, cyp11a*, *cyp11b activin BA*, *activin BB* were observed.<br>v) In testis, upregulation of *esr2a*, *lhr*,*ldlr*, *cyp19a*, *activin BA* occurred<br>vi) In brain downregulation of *gnrhR2* and *gnrhR3* occurred in females<br>vii) In males the *cyp19b* was downregulated in a concentration-dependent	Zhang et al., (2008a)

			manner (not significant).	
		105, 233, 405, 795 µg/L; reproductively active adult male and female fish exposed for 21 days in a FSTRA study following TG 229 guidelines	i) Survivability of the fish was affected by increasing concentration of KTC in the medium<br>ii) No alteration in growth (length and weight), HSI, and GSI of both male and female fish<br>iii) Number of anal fin papillae in the male fish increased in a nonlinear fashion; no alteration observed in females.<br>iv) Concentration-dependent decrease in liver VTG content occurred in both male and female fish	Onishi et al., (2021)
60.	11-ketotestosterone (11-KT)	2, 10, 50, 250 ng/mL, mature females exposed for 10 days	i) Mean total number of anal fin papillary processes had increased from approximately 10 (2µg/L) to 65 (250 µg/L).	Asahina et al., (1989)
		0.01, 0.1, 1.0 mg/L; 7dph larvae exposed for 96h depurated 90 dph.	i) Females significantly decreased body size and weight than controls, while males have larger body weight.	Leon et al., (2007)

			i) Thyroid follicular cell hypertrophy in both males and females.<br>ii) Germ cell necrosis in both males and females	
		100 µg/L, 1wph larvae (stage 40). exposed for 96 h, evaluated 3 months after exposure	i) In males 518 genes are upregulated and 650 genes are downregulated, related to sex differentiation and growth.<br>ii) In females 27 genes are upregulated and 116 genes are downregulated, related to sex-differentiation and growth.	Leon et al., (2008)
		0.1, 1.0, 10, and 100 µg/L, 8-month-old fish exposed for 21 days	i) The ratio of male medaka had risen [significant in 10 and 100µg/L) [NOEC= 1.0 µg/L; LOEC=10 µg/L).	Grillitsch et al., (2010)
		1, 10, 100 µg/L; fertilized eggs were exposed until 1 d before hatching (8 days) and evaluated 0, 30 and 100 dph	i) Decrease the ratio of masculinization (sex skewing; ratio of feminization was much lower than that of masculinization).	Watanabe et al., (2023)
61.	Letrozole (LET)	EXPOSURE 1:<br>1, 5, 25, 125, 625, 3125 µg/L, embryos (4hpf) exposed for 14 days.	EXPOSURE 1:<br>i) Hatchability, time to hatch, and gross morphology of the larvae did not alter.	Sun et al., (2007b)

		EXPOSURE 2:<br>1, 5, 25, 125, 625 µg/L, adult fish exposed for 21 days	EXPOSURE 2:<br>i) HSI (25 and 625 µg/L) and GSI (125 and 625 µg/L) in males increased.<br>ii) HSI in females decreased (25,125, and 625 µg/L); GSI increased in 625 µg/L.<br>iii) Fecundity and fertility decreased (125 µg/L)<br>iv) Ovary histology remained unaltered.<br>v) In testis, increase in lumina and sperm density in 625 µg/L group.<br>vi) Plasma vtg remained unaltered in males and decreased in females (concentration-dependent)<br>vii) Concentration-dependent increase in male population in F1 fish exposed in F0.	
		30, 100, and 300 µg/L,<br>Adult fish exposed for 72h	Brain:<br>i) No change in *esr1*, *esr2a*, or *arα* mRNAs in brain of female fish.	Sun et al., (2011a)

			ii) In males significant decrease in *esr1* in 100 - 300µg/L; while enhancement of *esr2a* mRNA in 30 µg/L group.<br>iii) No change in *cyp19a* in the brain of female and male fish except in 300µg/g where a reduction was seen.<br><br>Liver:<br>i) Down-regulation of *vtg1* and *vtg2* mRNAs in a concentration-dependent manner in females.<br>ii) *esr1* down-regulated, however *esr2a* upregulated only in females exposed to 100µg/L LET.<br>iii) *arα* remained unaltered.<br><br>Gonad:<br>i) No changes in *esr1* mRNA in females.<br>ii) *esr2a* increased in ovaries in 30 and 300 µg/L<br>iii) *arα* mRNA decreased by 300 µg/L in ovary	

			iv) In testis no alterations of *esr1*, *esr2a*, and *arα* mRNAs<br>v) *StAR* in ovary was upregulated only in 100µg/L, rest unaltered.<br>vi) *cyp11a* upregulated in ovary only in 100 µg/L group and *cyp11b* remained unaltered.<br>vii) *cyp17a* and *cyp17b* in the ovaries are upregulated.<br>viii) *cyp19a* in ovary enhanced in a concentration-dependent manner. *cyp19b* decreased in ovary<br>xi) in testis, *cyp11a* enhanced only in 30 and 100µg/L groups; *cyp11b* in 100 and 300µg/L groups.<br>xii) *cyp17a* and *cyp17b* in 100 µg/L, *cyp17b* in 100 and 300 µg/g, *cyp 19a* and *cyp19b* remained unaltered	
		50, 500 µg/L, embryos (FLFII), exposed at stage 10 until 42dph,	i) Male biased sex ratio (F1 embryos)<br>ii) No change in embryonic hatchability or	Liao et al., (2014)

		depuration 4-5 months	abnormalities in gross development<br>iii) Fecundity reduced.<br>iv) Fertility of the F1 embryos reduced; hatching rate of F1 embryos remained unchanged.<br>v) Secondary sexual features in males showed dose-dependent increase, with no change in females.<br>vi) Did not cause any sex reversal or intersex.<br>vii) Hepatic *cyp3A* activity inhibited in 50 µg/L while induced in 500 µg/L in males and females.<br>viii) Decreased expression of *esr1*, *vtg1*, *vtg2* and *cyp3A40* mRNAs in liver of males and *cyp19a* in testis<br>ix) Increased hepatic *cyp3A38*, and gonadal cyp*17A* in males; for females, expression of hepatic *esr1*, and *vtg1* and gonadal *cyp19A* was enhanced in 50	

			µg/L but inhibited with 500 µg/L<br>x) Expression of *cyp3A38* was upregulated in liver of females.	
	LET+E2	LET (10 or 50, or 250 µg/L+E2 (200 ng/L)<br>Adult 5 months age exposed for 21 days	i) No enhancement of plasma vtg in males, decreased in females.<br>ii) HSI decreased in males in a concentration dependent manner.<br>iii) GSI increased in males in a concentration-dependent manner.<br>iv) Impaired reproductive success at 250 µg/L lET+ E2 (200 ng/L)	Sun et al., (2009)
	LET+EE2	LET (30, 100, 300µg/L) + EE2 (20ng/L),<br><br>Adult fish exposed for 72h	Brain:<br>i) In males, transcription of *erα* reduced (not significant). Elevated expression of *esr2a* and *arα* was observed. *Cyp19b* unresponsive.<br>ii) In females, induction in *esr1*, *esr2a* and *arα* not significant; *cyp19b* reduced with highest LET (300 µg/L) coexposure.<br>Liver:<br>i) In males *vtg1* and *vtg2*, and *arα* transcription	Sun et al (2011a)

			reduced/down regulated; *esr1* and *esr2a* remained unaltered.<br>ii) In females, *vtg1* and *vtg2*, *esr1* decreased (concentration dependent with LET) [*esr2a* has upward tendency but not significant; *arα* no change.<br>Gonad:<br>i) In testis, concentration-dependent decrease in *esr1*, no change in *esr2a; arα* increased, *cyp11a* upregulated.<br>ii) In ovary, expression of *esr1*, *esr2a*, or *arα*; *StAR*, *cyp11a*, *cyp11b*, *cyp17A*, *cyp17B*, and *cyp19a* upregulated.	
62.	Levonorgestrel (LNG)	7.3, 42.2, 226 ng/L; reproductively active adult male and female fish were exposed for 21 days; TG 229 protocol	i) No significant difference was observed in survivability, growth (length and weight) and liver (HIS) and gonad (GSI) weight between control and LNG exposed male or female fish.	Onishi et al., (2021)

			ii) Liver vtg content of male fish did not change significantly, while in females a concentration-dependent reduction in liver vtg content was observed.<br>i) The number of papillae in the anal fin remained unaltered in male fish, while a concentration dependent increase in number of anal papillae was observed in female fish.<br>ii) Reproductive activity reduced (number of total eggs and fertilized eggs) in a concentration-dependent manner.	
		1,10,32,320, 1000 µg/L; 1 dpf embryos were exposed until hatch with semi-static renewal once in every 24; after hatching the larvae after 72 hph was evaluated	i) More than 80% of the embryos successfully hatched.<br>ii) Significant reduction in swim bladder inflation was observed	Pandelides et al., (2021)
		0.01, 0.1,1, 10, 100 µg/L; medaka embryos were	i) As the number of anal fin papillae increased and formation of	Watanabe et al., (2023)

		exposed for 8 days (1 day before hatching); samples were collected on 0 dph, 5 dph; 30 dph, and 100dph.,		ovotestis in XX females, the sex ratio (male: female) was decreased in a concentration-dependent manner (masculinization).<br>ii) Lower dose of LNG (<1 µg/L) induced feminization in XY fish (nonlinear)<br>iii) Significant transcriptional activities of *esr2a* and *esr2b*; no transcriptional activity was observed for esr1.<br>iv) Significantly reduced *chgH* expression in 5dph larvae in a concentration-dependent manner.<br>v) LNG significantly induced the expression of *arα* and *arβ* transcription.<br>vi) Expression of *chgH* mRNA 1 dph larvae (both male and female larvae) appeared to be biphasic in nature; enhanced in lower concentration (100 µg/L) and decreased in	

			higher concentration (300 µg/L).	
63.	Lignin-derived bisphenol (LD-BP)	2,4, 4.5, 5, 5.5 and 6 1.5 mg/L, juvenile fish (45-52 dph) exposed for 60 days	iii) The 96 h LC50 is 4.1 mg/L.<br>iv) Liver VTG in males are 125% higher than control males; 42% higher in females.<br>iii) The total number of eggs are 25% less than controls.<br>iv) GSI in females reduced; HSI in males and females enhanced.<br>v) The total number of broods was lower by 17% than controls.<br>vi) Catalase enzyme activity in liver, gill, testis, and ovary was reduced.<br>vii) SOD remained unaltered.<br>viii) Glutathions-transferase was enhanced in the liver and gill tissue. Malondialdehyde (MDA) activity elevated (liver, gill, intestine, testis, ovary)<br>ix) AChE in liver reduced 32%	Li et al., (2016; 2017)

			x) Intestinal α-glucosidase decreased significantly.<br>xi) In gonad histology, aggregation of interstitial cells and interstitial cell hyperplasia was observed.<br>xii) Atretic follicles in the ovary with late vitellogenic oocytes; follicular wall degeneration, interstitial cell fibrosis and possible increased perivascular smooth muscle was observed in the ovary.	
64.	Lincomycin (LNM)	0.42, 4.2, 42, 420, 4200 µg/L; Fertilized eggs were exposed until 90 dph	i) The survival of eggs and larvae was not affected<br>ii) Survivability of juvenile and adult fish was reduced in a concentration-dependent manner<br>iii) HSI and GSI remained unaffected<br>iv) The reproductive capacity remained unaltered<br>v) Hepatic VTG tended to increase (not significant)	Kim et al. (2012)
65.	Linuron (LNR)	7 mg/L, embryos (3-4 hpf) exposed for 7 days	xiii) EC_{50} is 8.5 mg/L<br>xiv) Downregulation of *cyp11b*, *3β-hsd*, and *gnrhr2*	Schiller et al., (2014)

		1,5, 10, 20 mg/L; 0 dph larvae (*chgH-gfp* transgenic lines) were exposed for 24h with or without E2 (180 ng/L or with and without testosterone (28.57 µg/L).	i) No mortality was observed in embryos exposed to linuron only (1-20 mg/L), however, embryos exposed to 10 and 20 mg/L are motion less.<br>ii) In the absence of testosterone, no fluorescence was detected in chgH-gfp fish.<br>iii) In presence of Testosterone or E2, linuron decreased fluorescence in a concentration-dependent manner	Spirhanzlova et al., (2017)
66.	Metformin (MET)	40, 120, 360 µg/L for 19 weeks.<br>F0 adults 4 weeks<br>F1 whole lifecycle 15 weeks.	F0:<br>i) Did not exhibit any significant change in fecundity, hatchability, and gross larval deformity<br>ii) Enhanced gene expression of *cyp19a*, and *esr1* in male fish.<br>iii) Decreased gene expression of *vtg2* and *esr2a* in females<br>iv) *vtg1* tended to increase in both sexes (not significant to controls)	Lee et al., (2019)

			v) Intersex occurred in females not in males<br>vi) Increased reactive oxygen species (ROS) and decreased glutathione content in male fish<br>vii) In females catalase activity increased<br>viii) In liver, cyp1a and cyp3a38 activities did not alter<br>ix) No change in the histology of liver, brain, kidney, gills, and thyroid<br>F1:<br>x) Did not exhibit any significant change in fecundity, hatchability, and gross larval deformity<br>xi) No change in *cyp19a* expression in both sexes<br>xii) In females *esr1* enhanced over control<br>xiii) In liver *vtg1* declined significantly in males, while *vtg2* did not change in both sexes<br>xiv) catalase and GST activity in liver did not alter	

			xv) intersex observed in Females<br>xvi) No change in the histology of liver, brain, kidney, gills, and thyroid	
67.	Methimazole (MTZ)	30 nM, embryos exposed to until 10 dpf (when swim bladder inflation is complete)	i) No mortality.<br>ii) Females are larger in length.<br>iii) No swim bladder inflation in females (~30%) and males (~10%).<br>iv) No change in gene expression (*tshβ, sp-a,sp-b.sp-c, trα, trβ, esr1,esr2a*) in males and females<br>v) *vtg* was enhanced in males, remained unaltered in females.	Godfrey et al., (2019)
68.	Methoxychlor (MXC)	0.2, 0.6, and 2.3 µg/L, 0dph fries exposed for 1 month and depurated until 56 dph.	i) No depreciation in reproductive capability as measured by fecundity, viability of eggs, or hatchability of eggs.<br>ii) No alteration in sex ratio.	Nimrod and Benson, (1998)
		5,10, and 20.0 µg/L, transgenic male fish exposed for 21 days.	i) No induction in vitellogenin mRNA was observed.	Zeng et al., (2005)

69.	3-(4-methylbenzylidene) camphor (4-MBC)	0.039, 0.39 and 3.9 mM; adult male fish were exposed for 1 week	i) Plasms VTG levels increased in a concentration-dependent manner.<br>ii) Enhancement in the serum VTG content and the expression of *vtg1*, *vtg2*, *chgL*, *chgH*, and the *esr1* mRNAs in liver in a concentration-dependent manner<br>iii) Expression of *esr2* and *ar* remained unaltered	Inui et al., (2003)
		5, 50 and 500µg/L, adult medaka exposed for 28 days	i) Fecundity and fertility were significantly decreased at 500µg/L.<br>ii) Spermatogenesis was inhibited in 50-500 µg/L.<br>iii) Significant decrease in plasma 11-ketotestosterone in male fish exposed to 50-500µg/L.<br>iv) Serum vitellogenin and estradiol level was increased in female fish exposed to 5 µg/L.<br>Brain:<br>v) The mRNA transcripts of *esr1*, *esr2a, arα, cyp 19b*, *fshb*, *lhb* in the brain of female medaka	Liang et al. (2020)

			were increased significantly (5-500 µg/L).<br>vi) The mRNA transcripts of *esr1*, *esr2a*, and *arα* was increased significantly in the brain of male fish (5-500µg/L).<br>vii) mRNA transcripts of *lhb* at 50-500 µg/L, and *fshb* and *cyp19b* at 5µg/L in brain of male fish increased.<br>Liver:<br>viii) The mRNA transcripts of *arα* and *StAR* significantly decreased in the liver of male fish exposed to 500 µg/L<br>ix) The mRNA of *vtz* in liver of female fish increased 5-50µg/L; males 5-500µg/L<br>x) The mRNA transcripts of *esr1* and *esr2a* in the liver of females in 50 µg/L, in male liver in 50-500 µg/L increased.<br>Gonad:<br>xi) The mRNA transcripts of *esr1*, *esr2a*, *cyp17α*, *hsd3b*, *star*, *fshr*.*lhr*, are	

			increased in testis (5-500 µg/L)<br>xii) The mRNA transcripts of *lhr* and *vtg*, in the ovary was upregulated (5-500µg/L).<br>xiii) The mRNA transcripts *arα*, *esr2a*, *cyp17α* in the ovary was upregulated only at 500 µg/L.	
70.	Methylparaben; (MPB)	0.01, 0.04, 0.2, 1,5, and 25 mg/L.<br>Adult male fish exposed for 14 days	i) Plasms VTG content increased in a concentration-dependent manner.<br>ii) Upregulation of 13 genes including vtg2, chgL, chgH, chgHm, esr1 occurred.	Yamomoto et al., (2011)
		0.357,1.90, 9.75 mg/L, 16±2 weeks fish were exposed for three weeks (TG229 assays);	i) Hepatic vtz content in males increased in a concentration dependent manner; females remained unaltered.<br>ii) Secondary sex characters unaltered in males and females.<br>iii) Number of total eggs, fertilized eggs and fertility rates were decreased in highest concentration used (9.75 mg/L).	Kawashima et al., (2022)

71.	Methyltestosterone (MT)	0.8, 8, 80, 800, 8000 ng/egg.<br>Fertilized eggs were injected and evaluated 103 dpf	i) Sex reversal occurred in XX females in a dose-dependent manner.<br>ii) Gonad weight (GSI) in both XX males and XY males remained equal.	Papoulias et al., (2000)
		0.02,0.2, 2, 20, and 40 mg/g diet, adult fish were fed for 7 days	i) Inconsistent enhancement of serum vtg in males (0.02-0.2 mg/L).<br>ii) In females serum vtg decreased in 20 mg/g diet.	Chikae et al., (2004)
		25,50, 100, 200, 400 ng/L;<br>adult male and females exposed for 21 days.	i) Liver vtg content did not change in males; in females, significant decrease was observed in last two concentrations exposed for 21 days.	Nozaka et al., (2004)
		0.35, 1.09, 3.29, 9.98, 27.75 ng/L;<br>Embryos (12hpf) were exposed until 101 days.	i) Induced masculinization in both secondary sex characters and gonads (testis-ova).<br>ii) Swollen abdomen with enlarged ovaries with declined fecundity and fertility.<br>iii) No embryological abnormalities or hatching failures of fertilized eggs in F1 fish	Seki et al., (2004)

			iv) No abnormalities observed in growth, sex-ration or in appearance of secondary sex characteristics in F1 fish.<br>v) Hepatic vtg level in F1 males increased in a concentration-dependent manner (9.98 ng/L) and decreased in F1 females exposed to all MT concentrations.	
		25, 50, 100, 200, 400 ng/L, 0 dpf fries exposed until 60 dph.	i) Masculinization of female fish with regard to secondary sex character.<br>ii) Fecundity and fertility significantly decreased in concentrations 50 ng/L and above.<br>iii) GSI of females increased significantly in fish exposed to 50 ng/L and above.<br>iv) Degeneration of oocytes occurred in all MT exposed groups.<br>v) Hatchability and survival rate of offspring decreased in all MT groups.	Kang et al., (2008)

			vi) Hepatic vtg in females decreased significantly in 200 and 400 ng/L groups.	
		0.32 nM; female medaka were exposed for 18 h- 52 days	i) Females developed papillary processes in the posterior anal fins.<br>ii) *arα, arβ* mRNA expression remained unaltered in the posterior anal fins of females.<br>iii) mRNA expression of *bmp4, bmp7, lef1* were enhanced in posterior anal fins after 10 d MT treatment.	Ogino et al., (2014)
		1, 10 mg/L; Fertilized eggs were exposed until hatching, evaluated 60 dph	i) Male biased sex ratio in XX female genotypes.<br>ii) Induced *gsdf* mRNA accompanied by sex reversal of XX genotypes	Horie et al., (2016)
		0.2,1,5,25 mg/L, fertilized eggs exposed until 8dpf, depurated until maturity	i) XX fish were masculinized, frequencies decreased in a concentration-dependent manner.	Myosho et al., (2019)
		20.1, 77.4, 300 ng/L; reproductively adult male and female fish were exposed for 21 days (FSTRA assay	i) No effect was observed on growth (length and weight), HSI, and GSI.<br>ii) Secondary sexual features (papillae in the	Onishi et al., (2021)

		following TG 229 guidelines)	anal fin) increased in female fish in a concentration-dependent manner with no effect on male fish.<br>iii) Reproductive activity (fertility and fecundity) impaired in a concentration dependent manner.<br>iv) Liver VTG content remained unaltered in males while decreased significantly in females in a concentration-dependent manner.	
72.	Metolachlor (MTC)	1,10,100, 1000 µg/L One (juveniles)-and 4 month (adult)- old medaka exposed for 14 days	i) *dio2, trα, trβ* mRNA levels increased significantly in juvenile medaka females (not in males).<br>ii) Thyrotropin releasing hormone (trh) mRNA showed non-linear significant enhancement (only in 100 µg/L) only in females.<br>iii) The brain of adult female fish showed concentration-dependent enhancement in the mRNA levels of *trh, trα, trβ, dio2* genes;	Jin et al (2011b)

			males remained unresponsive.<br>iv) The expression of *trα* and *trβ* genes remained unaltered in the liver of both male and female adult medaka fish exposed to Metolachlor.<br>v) The expression of *dio2* mRNA in females was found to be increased in a concentration-dependent manner.	
73.	Myclobutanil (MCB)	EXPOSURE 1:<br>2 and 3.5 µM;<br>Adult fish (7 months old) for 3 and 7 days<br><br>EXPOSURE 2:<br>2, 3.5, and 5 µM	i) Significant increase of cyp1a and cyp3a activities after 7 days exposure, not after 3 days exposure	Lin et al., (2014)
74.	Naproxen (NPX)	0.005, 0.5, 5, and 50 mg/L, fertilized eggs exposed for 30 dph (OECD TG 210)	ii) Survivability was decreased (juvenile fish) at 0.5 mg/L; length, weight, hatchability, and time to hatch did not alter.<br>iii) Transcription of *vtg1*, *erβ2*, *cyp17* genes significantly increased at 5, 0.5, and 5 mg/L, respectively; expression of *cyp19a* remained unaltered.	Kwak et al., (2018)

75.	1-naphthol (1NT)	80, 258, 857 µg/L, 16±2 weeks medaka were exposed for three weeks (TG229)	i) Hepatic VTG unchanged in male but enhanced in females (highest concentration, 857 µg/L).<br>ii) Secondary sexual characters remained unchanged in males and females.<br>iii) GSI in female increased in highest concentration (857 µg/L).<br>iv) Number of total eggs, fertilized eggs appear to be reduced at highest concentrations (857 µg/L), however fertility rate significantly reduced at highest concentration (857 µg/L).	Kawashima et al., (2022)
76.	N-Nitrosodiethylamine (DEN)	Embryos:<br>0.01, 0.1, 1, 10 ng/L; embryos 1 hpf were exposed until 18 18dph<br>Adults:<br>1 and 10 ng/L for 10 days	i) Significantly reduced germ cell number in the ovary of XX (not in XY) fish in a concentration-dependent manner	Nair et al., (2017)
77.	4-nonylphenol (4-NP)	10, 50, 100 µg/L; post-hatch larvae (1-2 dph) exposed for 90 days.	i) Testis -ova formation in males is concentration-dependent.<br>ii) Female-based sex ratio.	Gray and Metcalfe, (1997)

		0.5, 0.8, 1.9 µg/L, 0 dph larvae exposed for 30 days, harvested on 56 days.	i) No depreciation in reproductive capability as measured by fecundity, viability of eggs, or hatchability of eggs.<br>ii) Increased egg production [0.54 µg/L).<br>iii) More males than females (90.77 µg/L)	Nimrod and Benson, (1998)
		0.03, 0.1, 0.3 µM/L, male fish were exposed for 2 weeks	i) No effect on fecundity and hatchability.	Shioda and Wakabayashi, (2000)
		0.1, 10, and 100 µg/L,<br>0 dpf embryos exposed for 230 days;<br>Adult males were exposed for 5 weeks	i) LC50 for embryos is 0.13 mg/L; for adults 0.13 mg/ L.<br>ii) 100 µg/L have abnormal gonad.<br>iii) Mature male medaka are exposed to (0.1, 10, and 100 µg/L for 5 weeks) found female-specifc protein in the blood (vitellogenin) in all concentrations after 5 weeks of exposure.	Tabata et al., (2001)
		<u>F0 fish</u>:<br>4.2, 8.2, 17.7, 51.5, 183 µg/L,<br>F0 fish exposed from 24 hpf to 104 days.<br><u>F1 fish</u><br>4.2, 8.2, 17. 7 µg/L,	i) 20 % male with testis ova and high mortality (F0)<br>ii) Sex ratio skewed towards female in 51.5 µg/L group (F0)	Yokota et al., (2001)

		F1 fish exposed 0 dpf until hatching.	iii) Testis-ova observed (17.7 and 51.5 µg/L (F0)<br>iv) LOEC is 17.7 µg/L and NOEC is 8.2 µg/L (F0)<br>v) No post hatch mortality or growth inhibition of larvae (F1)<br>vi) testis-ova were observed in 8.2 and 17.7 µg/L (F1)	
		2, 20, and 50 µg/L, Male medaka exposed for 7 days	i) The LOEC for vtg induction in male medaka are 20 µg/L	Islinger et al., (2002)
		0.1, 10, and 100 µg/L, male medaka exposed for 1,2,3, and 5 weeks	i) LC_{50} values of adult male and female medaka (72 h exposure) 0.85 mg/L, for females 0.87 mg/L.<br>ii) LC_{50} values for embryos 0.13 mg/L.<br>iii) IC_{50} for inhibition to egg hatching were 0.85 mg/L.<br>iv) Mature medaka (male) were exposed to 0.1,10, or 100 µg/L of NP for 5 weeks. Female specific proteins (FSP) were induced in the blood higher than 0.1 µg/L.	Kashiwada et al., (2002)
		5, 50, 100, 200 and 500 µg/L, Mature	i) *chgL* mRNA was induced in 50 -500 µg/L	Lee et al., (2002)

		male medaka were exposed for 144h	in a concentration-dependent manner.<br>ii) *chgH* mRNA expression was observed in 100 µg/L.	
		24.8, 50.9, 101, and 184 µg/L, adult fish exposed for 3 weeks	i) Males with testis ova (24.8 µg/L).<br>ii) Decreased egg production (100 µg/L).<br>iii) Reduced GSI, low fertility and abnormal spermatogenesis in males (184 µg/L).<br>iv) Hepatic vtg enhanced in both sexes (50.9 µg/L and above).<br>v) LOEC for reproductive capacity (fertility and fecundity is 101 µg/L).<br>vi) LOEC for eliciting estrogenic response (induction of testis-ova and hepatic vtg is ≤24.8 µg/L	Kang et al., (2003)
		3.13, 6.25,12.5, 25, and 50 µg/L, fertilized eggs exposed until 60dph	i) LOEC is 11.6 µg/L (sexual differentiation and hepatic vitellogenin induction).<br>ii) Body weight significantly decreased in 25 and 50 µg/L.<br>iii) Significant enhancement of testis	Seki et al., (2003a)

				ova formation (50 and 100 µg/L).	
			iv)	VTG induction in liver occurred in both male and female fish [11.6 µg/L and above].	
			v)	Secondary sexual features reversed during depuration, while testis-ova still exists.	
		7.5, 15, 30, 60, 120 µg/L; adult male and female fish exposed for 21 days	i)	Concentration-dependent enhancement of liver vtg observed in males; in females only, significant increase was observed in the highest concentration used.	Nozaka et al., (2004)
		500 µg/L; adult male fish exposed until 1-, 3- and 12-days exposure	i)	The concentration of VTG in both liver and testis became gradually higher with longer treatments.	Kobayashi et al., (2005)
		50,100, 500, 1000 µg/L, transgenic male fish (*mvtg1:gfp*; the gfp reporter gene is under the control of medaka *vitellogenin1* gene promoter) were exposed for 21 days.	i)	*vtg1* mRNA in liver remained unaltered.	Zeng et al., (2005)

		1, 3, 10, 30, 100 µg/L, 1dpf larvae exposed from 1-100dpf	i) No difference in sex ratio<br>ii) Fish with mixed secondary sexual characteristics and gonadal intersex increased in 30 and 100 µg/L concentrations	Balch and Metcalfe, (2006)
		10, 50, 100µg/L, adult medaka exposed for 21 days	i) Reduced egg production and fertility.<br>ii) Hepatic vtg increased significantly in all three concentrations in males.<br>iii) In F1 generation the hatchability and time to hatching was adversely affected in 100µg/L group.<br>iv) Spawned eggs showed 4-NP accumulation at 2-7 µg/g eggs.	Ishibashi et al., (2006)
		5 and 50 ng/L<br><br>EXP1: newly hatched medaka exposed for 7 days at 10 °C-10L, 20°C-12 L, 30 °C-14L)<br><br>EXP2: 1 month old juvenile medaka of both sexes were exposed for 7 days	EXP1:<br>i) Significant induction was observed in vtg1 mRNA in 30°C-14L group when exposed to 50 µg/L NP.<br>ii) No induction of *vtg2* or *esr1* mRNAs occurred.<br><br>EXP2:<br>i) The mRNA transcripts of *vtg1* and *vtg2* of	Jin et al., (2011a)

		(10 °C-10L, 20°C-12 L, 30 °C-14L<br><br>Exp 3:<br>Four-month-old were exposed for 7 days (10°C-10L; 20°C-12L, 30°C-14L)	males and females was increased at higher NP (50 µg//L) in all three temperature and photoperiod regime in males and in females (except 10 °C-10L groups).<br>ii) No induction of *esr1* mRNA occurred in males but in females occurred in 20 °C-12L (50 µg//L NP).<br><br>EXP3:<br>i) The increase of estrogen-responsive genes in liver of male and female (except *esr1*) medaka increased in all concentrations and all temperature and photoperiod regime.<br>ii) *esr1* mRNA increased in 50 µg//L NP in 20 °C-12 L and 30 °C-14L groups in males only, no induction in females in any concentrations or temperature/photoperiod regime.	
		0.1,1,10, and 100 ng/mL, transgenic	i) Expression of *gnrh1, gnrh2* and *gnrh3* mRNA	Lee et al., (2012)

		medaka embryos (gfp protein is under the control of *gnrh3* promoter) were exposed until hatching	was down regulated by 100 ng/mL NP.<br>ii) *Esr1* mRNA expression was upregulated at 100 ng/mL NP.<br>iii) Expression of *gnrhR1*, *gnrhR2*, *gnrhR3* mRNAs and *esr2a*, and *esr2b* mRNAs remained unaltered.<br>iv) Other features such as heart rate, eye pigmentation, head growth, and time to hatch is also affected.	
		esr1 reporter gene assay	i) Transcriptional activities mediated by ESR was 0.036 µM [relative potency 0.471%, considering E2 as 100].	Miyagawa et al., (2014)
		In vitro reporter gene assay; gene transcriptional activities for E2	i) ESR1= EC_{50}= 6.20 x10 $^{-7}$ M (relative potency=0.021% considering estradiol as 100).<br>ii) ESR2a= not detected.<br>iii) ESR2b=EC_{50}= 8.64X10 $^{-7}$ M (relative potency= 0.0094% considering estrogen as 100).	Tohyama et al., (2015)
		1.27, 2.95, 9.81, 27.8, 89.4 µg/L, MEOGRT	i) F0 fish unaffected in terms of reproduction;	Watanabe et al., (2017)

		experimental condition (3 generation; 20 weeks) (F0= 3 weeks adult; F1=15 weeks full life span; F2=2 weeks (until hatching);<br>F0 adult fish exposed for 3 weeks		liver vtg increased in 2.95 µg/L and above concentrations.<br>ii) In F1, fecundity, fertility, and number of fertile eggs reduced with increasing concentrations.<br>iii) LOEC for total eggs in F1 is 1.27µg/L; for fertile eggs 1.27 µg/L, for fertility 27.8 µg/L.<br>iv) In F1, but not in F0, secondary sex characteristics (anal fin papillae in males) were suppressed in 27.8 µg/L concentration.<br>v) Vitellogenin induction in adult male liver is slightly weaker in F1 than F0.<br>vi) Gonadal sex abnormality and sex reversal occurred at 27.8 and 89.4 µg/g concentrations in F1 subadults. At 89.4 µg/L all genotypic F1 males in breeding pairs had female phenotype and some even demonstrate spawning.	

			vii) Concentrations lower than 89.4 µg/L did not affect F2 survival or hatching.	
		EXPOSURE 1:<br>5 hpf embryos exposed to NP (0.71-22.7 µM) for 14 days.<br><br>EXPOSURE 2:<br>1 dph larvae exposed for 96 h with NP (0.71-22.7 µM)<br><br>EXPOSURE 3:<br>5 hpf exposed for 7 days and 9dpf (newly hatched) for 3days to 0.09-1.5 µM NP.	i) Hatchability decreased significantly (2.8 and 5.7 µM); embryos exposed to 11.3 and 22.7 µM did not hatch.<br>ii) 96h LC_{50} for is 2.0µM (larvae); 14 d LC_{50} for embryos 2.7 µM.<br>iii) Embryonic exposure (5 hpf to 7 dpf) did not show any alteration in the mRNA expression level in nine genes (*chgL*, *chgH*, c*hgHm*, *vtg1*, *vtg2*, *cyp19a1b*, *esr1*, *esr2a* and *esr2b*.<br>iv) *chgH* upregulation in 1.5 µM was observed in 9dpf embryos exposed from 5 hpf to 9dpf (immediate hatching).<br>v) 9 dpf (immediate hatched larvae) exposed for 3 days (until 12 dpf) were also unresponsive to (0.09-1.9 µM) about 9 genes mentioned above.	Ishibashi et al., (2020)

		EXPOSURE 1:<br>32 or 100 µg/L, 4hpf exposed for 30 and 60 days.<br><br>EXPOSURE 2:<br>1-100µg/L, 4hpf-until stge38 of development<br>[TG 234; fish sexual development test OECD 2011]	i) At stage 38 (just before hatching) exposure at 1-100 µg/L did not affect *gsdf* expression in XX embryos.; in XY embryos the *gsdf* expression in the 100 µg/L was significantly lower than controls.; no significant difference in the expression of *foxl2*, *cyp19a1a* genes.<br>ii) In XY medaka at 60 dph, the number of fin ray joint plates showing papillary processes decreased as the exposure concentration increased; at 100 µg/L no papillary processes were observed.; no papillary processes were observed in female secondary sexual phenotypes. (LOEC for NP is 3.2 µg/L).<br>iii) Testis-ova seen in NP exposed XY fish on 30 dph as well as 60 dph (from 32 µg/L and above; 1, 3.2 and 10 µg/L NP exposed XY fish has normal testis).	Horie et al., (2021)

			XX fish has normal ovaries.<br>iv) Hatching rate unaffected; total body weight, total length unaffected.	
		5.63, 18.8, 51.8, 170 µg/L, 16±2 weeks medaka were exposed for three weeks (TG229 assays)	i) Hepatic vtz enhanced in male in a concentration-dependent manner, while in female enhancement is not concentration-dependent.<br>ii) HSI enhanced in male at 51.8 and 170 µg/L concentrations.<br>iii) Secondary sex characters remained unaltered in both male and females.<br>iv) Number of total eggs, fertilized eggs and the fertility rate decreased at highest two concentrations (51.8 and 170 µg/L).	Kawashima et al., (2022)
	Nonylphenol ethoxycarboxylate (NP1EC)	100, 300, 1000 and 3000 µg/L,<br>1 dph larvae exposed for 100 days	i) Mixed secondary sex characteristics observed (not significant)<br>ii) Absence of testis-ova.	Balch and Metcalfe, (2006)
	Nonylphenol ethoxylate mixture (NP1EO)	10, 30, 100, 300 µg/L, 1 dph larvae	i) 300 µg/L treatment induced mixed secondary sex	Balch and Metcalfe, (2006)

		exposed for 100 days.	characteristics and suppressed papillae development.<br>ii) Absence of testis-ova.	
	Nonylphenol monoethoxylate/ nonylphenol diethoxylate mixtures (NP1EO/NP2EO)	25, 50, 100 µg/L, 1dph larvae exposed for 85-110 days	i) No change in sex ratio.<br>ii) Testis-ova seen only in one fish.	Metcalfe et al., (2001)
	Nonylphenol ethoxylate mixture (NP4EO)	10, 30, 100, 300, 1000 µg/L, 1 dph larvae exposed for 100 days	i) Mixed secondary sex characteristics observed only in 30 and 300 µg/L groups (not significant).<br>ii) Absence of testis-ova.	Balch and Metcalfe, (2006)
	Nonylphenol ethoxylate mixture (NP9EO)	30, 100, 300, 1000 µg/L, 1 dph larvae exposed for 100 days	i) Mixed secondary sex characteristics observed only in 300 µg/L groups (not significant).<br>ii) Absence of testis-ova.	Balch and Metcalfe, (2006)
	Nonylphenol monoethoxycarboxylate/ nonylphenol diethoxycarboxylate (NP1EC/ NP2EC)	50 and 100 µg/L, 1dph larvae exposed for 85-110 days	i) No change in sex ratio.<br>ii) No testis-ova seen.	Metcalfe et al., (2001)
78.	Octocrylene (OCL)	5, 50, and 500 µg/L, adults (3-4 months old) exposed for 28 days	F0<br>i) Significant increase in HSI and GSI of F0 medaka (500 µg/L).<br>ii) No difference in fecundity.<br>iii) Fertility significantly decreased in all doses.	Yan et al., (2020)

			iv) Significant increase in the per cent of mature oocytes (5 and 500µg/L) in ovary; in testis, inconsistent alteration was observed in spermatogonium and spermatozoa.<br>v) Plasma sex hormones (E2 and 11-KT) and VTG levels significantly increased in males in all treatments and in females only in 50 and 500 µg/L.<br>vi) The *fshβ*, and *lhβ* in the brain and *fshr*, *lhr* and *cyp17α* were upregulated in males in all treatment groups.<br>vii) The levels of *ar*, *esr1*, *esr2a*, *cyp19β* mRNAs in the brains of males and females were upregulated.<br>viii) The upregulation of *vtg* in male and female livers was observed only in 500µg/L and upregulation of *StAR* and *hsd3β* was observed in testis in all treatment groups.	

			F1:<br>i) Continued exposure of the F1 embryos induced increase in the time of hatching, morphological abnormality rates, and cumulative death rates	
79.	Octyl-methoxycinnamate (OMC)	0.034, 3.4, and 34 mM; adult male fish exposed for 1 week.	i) Plasms vtg level increased in a concentration-dependent manner.<br>ii) mRNA expression pattern of vtg1, vtg2, chgL, chgH, esr1, enhanced ina concentration-dependent manner.<br>iii) mRNAs of esr2a and ar did not altered.	Inui et al., (2003)
80.	4-octylphenol (4-OP)	2, 20, and 50 µg/L, fertilized embryos (2-4 hpf) exposed for 98 days	i) The length and weight of F1 males and females significantly decreased (50 µg/L).<br>ii) Fertilization rate in F1 decreased in a concentration-dependent manner.<br>iii) Female biased sex-ratio<br>iv) Low fertilization ratio (20 µg/L)<br>v) 3% F1 males developed testis ova (50 µg/L).	Knorr and Braunbeck (2002)

			vi) In F2 larval deformities observed (20 µg/L and above).<br>vii) Exposed males and unexposed females showed decrease in fertilization rates.	
81.	Oryzalin (OYZ)	EXPOSURE 1:<br>1.5,2.2, and 3.3 mg/L, 7-month-old fish exposed for 3 days.<br><br>EXPOSURE 2:<br>0.25, 0.5, and 1mg/L, exposed for 16 days.<br><br>Exposure 3:<br>0.25,0.5, 1 mg/L Adult male or female medaka exposed to oryzalin for 21 days	i) Induction of choriogenin in liver occurred exposed either 3 days (3.3 mg/L) or 16 days (0.5 mg/L).<br>ii) No induction of liver *vtg* in any of the exposure conditions.<br>iii) Exposure of either male or female fish to oryzalin, significantly increased the number of unfertilized eggs laid by the females.<br>iv) Males exhibited significant increase in necrotic spermatids in the testis.<br>v) Females exhibited hyperplastic ovaries with fewer immature oocytes.	Hall et al., (2005; 2007)
82.	Oxyfluorfen (OXF)	0.5,1,2,4,8 mg/L, 1dpf embryos and 2 dpf larvae exposed	i) The calculated 96 h LC_{50} for larvae is 5.238 mg/L.	Powe et al., (2018)

		for 96 h, depurated 1week	ii) Embryo mortality significantly enhanced (2 mg/L).<br>iii) Skeletal growth inhibited (2 mg/L).<br>iv) Scoliosis in larvae.<br>v) RNA content reduced while total protein content remained unaltered in larvae (2 mg/L).<br>vi) Significant upregulation of zic5 mRNA in larvae.	
83.	Oxytetracycline (OTC)	5 and 50 mg/L, male medaka exposed for 14 days	i) E2 concentration in the blood did not change.	Ji et al, (2010).
		0.005, 0.05, 0.5, 5, 50 mg/L<br>Fertilized eggs were exposed for 90 days (TG 210)	i) Hatchability (NOEC=50 mg/L), time to hatch (NOEC= 50 mg/L) and survival and growth of larvae and adults (NOEC=50 mg/L) exhibited no significant difference.	Ji et al., (2012)
84.	Pendimethalin (PDM)	5.69, 28.8, 100 µg/L, 16±2 weeks fish were exposed for three weeks (TG229)	i) Hepatic vtg enhanced in male at highest dose (100µg/L), but no change in females.<br>ii) Secondary sexual characters unaltered in male and females.<br>iii) Total eggs unaltered, fertilized eggs and	Kawashima et al., (2022)

			fertility rates reduced at highest concentration (100 µg/L).	
85.	Pentachlorophenol (PCPL)	10, 20, 50, 100, 200 µg/L, adult medaka exposed for 28 days.	i) Length and weight of both males and females significantly decreased in 200 µg/L group.<br>ii) Plasma vtg level in males enhanced (non-linear enhancement); in females significant decrease seen in fish exposed to 100 and 200 µg/L.<br>iii) EROD activities in liver microsomes of females enhanced (nonlinear dose-response).<br>iv) Testis-ova observed in males exposed higher than 10 µg/L.<br>v) Degenerative ovary observed in females exposed to 50 µg/L or higher.<br>vi) Lesion in liver observed in males exposed to 10 µg/L and higher.<br>vii) Lesion in kidney observed in male medaka exposed to 20 µg/L or higher.	Zha et al., (2006)

			viii) Fecundity and fertility reduced in fish exposed to 100 µg/L and above.<br>ix) Hatching delay observed in embryos (4th week) exposed to 200 µg/L).<br>x) Reduced hatching rates observed from 4th week in fish exposed to 100 µg/L and above.<br>xi) In F1fish hatching rate and hatching time significantly affected in fish exposed to 200 µg/L.<br>xii) No difference in sex ratio (F1).	
86.	Perfluorobutyric acid (PFBA)	137 mg/L, embryos exposed until 10 dpf (when swim bladder inflation is complete)	i) Standard length of females increased than controls.<br>ii) No mortality<br>iii) No swim bladder inflation in males and females.<br>iv) No change in gene expression in females and males.	Godfrey et al., (2019)
		5, 10, 20, 40, 80 mg/L; 2 hpf embryos exposed until 4hph.	i) Swim bladder inflation was inhibited (LOEC: 40 mg/ml).<br>ii) No change in expression of *tshβ*, *trα*, and *trβ*.	Horie et al., (2022d)

87.	Perfluorooctanoic acid (PFOA)	0.1, 1, and 10 mg/L; F0 fish exposed for 14 days; F1 fish until 100 days hatching	i) The fecundity in F0 fish (number of eggs produced per breeding pair) reduced in a concentration-dependent manner.<br>ii) Survivability of F1 fish decreased in a concentration-dependent manner.<br>iii) The HSI and GSI in F0 fish remained unaltered.<br>iv) In F1 fish the thyroid follicles showed hyperplasia, hypertrophy, and colloidal depletion.	Ji et al., (2008)
		0.3, 3, and 30 mg/L; Adult fish (F0) exposed for 28 days; F1 and F2 fish additional 238 days (total 259 days)	i) Fecundity suppressed over three generations (F0-F2) in a concentration-dependent manner.<br>ii) Hatching rate, HSI, GSI, and *vtg* expression did not change significantly.<br>iii) In F1 and F2 accelerated gonad development was observed.<br>iv) Sex ratio remained unaltered.	Lee et al., (2017b)
		10 mg/L, Adult fish exposed 7,14 and 21 days.	i) NOECs for PFOA is 10 mg/L (21 days exposure).	Kang et al., (2019)

			ii) Fecundity (number of daily egg production) reduced.<br>iii) secondary sexual features in males (number of papillary processes in the fin rays) remained unaltered.<br>iv) *vtg1* genes in the liver of male medaka significantly enhanced after 7-, 14-, and 21-days exposure.<br>v) *vtg2* gene reduced after 14- and 21-days exposure.<br>vi) In females both *vtg1* and *vtg2* genes enhanced after 7,14-, and 21-days exposure.<br>vii) *chgH* and *chgHm* mRNA expression in liver increased and gradually reduced over time in male. *cghl* mRNA expression remained at the reduced state in liver of male fish until 21st day of exposure.	

			viii) VTG protein in liver of male fish reduced significantly over time.	
		4.7 mg/L, embryos were exposed until 10 dpf (after completion of swim bladder inflation)	i) Standard lengths increased in both males and females.<br>ii) Female displayed larger swim bladder, no mortality.<br>iii) Increased *tshβ*, *sp-b*, *trβ* and *vtg* in females.<br>iv) Upregulation of *esr2a* and *vtg* in males.	Godfrey et al., (2019)
88. .	Perfluorooctane sulfonic acid (PFOSA)	0.01, 0.1, 1 mg/L; F0 fish exposed for 14 days and F1 fish until 100 days hatching	i) No mortality: fecundity decreased in a concentration-dependent manner (F0 fish).<br>ii) The GSI in female F0 fish increased significantly than controls.<br>iii) The HSI in females increased in a concentration-dependent manner.<br>iv) In F1 fish, total length and weight significantly decreased than controls; GSI in male enhanced.<br>v) Histopathological alterations in thyroid follicles occurred in both F0 and F1 fish.	Ji et al., (2008)

	Perfluorooctane sulfonate (PFOS)	1 mg/L, adult fish exposed for 21 days	i) NOEC is 1 mg/L.<br>ii) Fecundity (number of daily egg production) reduced.<br>iii) secondary sexual features in males (number of papillary processes in the fin rays) remained unaltered.<br>iv) *vtg1* genes in the liver of male medaka significantly reduced after 14-days exposure. *Vtg2* remained unaltered. In females both vtg1 and vtg2 genes remained unaltered.<br>v) *ChgH* mRNA expression in male liver significantly decreased, *chgHm* decreased on 7- and 21-days observation and *cghL* only on 7 days of exposure.<br>vi) Vitellogenin protein in male remained unaltered.	Kang et al., (2019)
89	Perfluoroalkyl acid (PFAA)<br>[mixture of perfluorooctane	2, 20 µg/L, exposed for 3 generations: 238 days, [TG 229 and 240]	i) Male biased sex ratio in F1[20 µg/L).<br>ii) Significant increase of vitellogenin expression	Lee et al., (2017a)

	sulfonate (PFOS) perfluororoctanoic acid (PFOSA), perfluorobutane sulfonate (PFBS) perfluorononanoic acid (PFNA) with the ratio of 1:1:1:1]		only in F2 generations in male fish.<br>iii) Fecundity was suppressed as generation goes from F0 to F2.	
90.	Phenanthrene (PHN)	EXPOSURE 1:<br>0.32 (25%), 0.64 (50%), 0.96 (75%) and 1.28 (100%) ppm; fertilized eggs were exposed until hatching. After hatching larvae were fed with 11.8, 19.2, 32.2, 55 ppm in food for 60 days<br>EXPOSURE 2:<br>1.28 mg/L; fertilized eggs were exposed and the larvae after hatching continued exposure for 60 days	i) delayed hatching<br>ii) fecundity did not alter<br>iv) HIS, GSI did not alter<br>v) Liver *vtg* content did not alter<br>vi) Histology of testis and ovary did not alter	Horng et al., (2010)
91.	Phenytoin (PHT)	2.25, 4.76, 8.72, 18.5 mg/L, 16±2 weeks fish were exposed for three weeks (TG229)	i) Hepatic vtg in male and female unresponsive.<br>ii) HSI in males and GSI in females enhanced in 8.72 and 18.5 mg/L concentrations.<br>iii) Secondary sex characters unaltered.	Kawashima et al., (2022)

			iv) Number of total eggs, fertilized eggs, and fertility rate decreased in 8.72 and 18.5 mg/L concentration.	
92.	Polychlorinated biphenyls 126 (PCB 126)	0.01 µg/g; sexually mature male and female fish were fed for three weeks	i) Expression of *chgL*, *chgHm*, and *arα* in liver of both male and female fish decreased<br>ii) The vtg1 expression was decreased in male and increased in female fish	Nakayama et al., (2011)
93.	Prochloraz (PCZ)	30, 30 and 300 µg/L Adult fish exposed for 7 days	i) Concentration- and time-dependent reduction in fecundity.<br>ii) A concentration-dependent down regulation of *erα, vtg1, vtg2*, *chgL*, *chgH*, *chgHm* in liver of females.<br>iii) In males a concentration-dependent downregulation of *vtgII*, *chgH*, *chgHm* and upregulation of *erβ* in liver.<br>iv) Concentration-dependent upregulation of *cyp17* and *cyp19a* and downregulation of *activinBA* occurred in ovaries of females.	Zhang et al., (2008a)

			v) In testis, concentration-dependent upregulation of *arα*, *3β-hsd*, *cyp11b*, *cyp11a*, *cyp17*<br>vi) In brain, *gnrhII*, *gnrhIII*, and *cyp19b* were downregulated in females, but in males only *cyp19b* downregulated.	
		4.5 mg/L, embryos (3/4 hpf) exposed for 7 days.	i) *cyp11b*, *3β-HSD*, *gnhrh2*, *cyp19a1b* were downregulated and androgen receptor (*ar*) was repressed (compared to flutamide 6.2 mg/L for 7dpf)	Schiller et al., (2014)
		5.3,9.2,17.5,25.0, 41.1 µg/L; MEOGRT protocol (multiple generation; F0, F1 and F2; total duration 29 weeks)	i) LOEC for fecundity was 41 µg/L (F0)<br>ii) reduced hatching (LOEC 25 µg/L)<br>ii) in liver of females (XX) the *vtg* mRNA level reduced (LOEC 9 µg/L)<br>iii) modest reduction of anal fin papillae in F1 XY fish (subadult), but not in adults of F0, F1 or F2 (LOEC 33µg/L)<br>iv) decreased yolk and follicular hyperplasia in XX fish	Flynn et al., (2017)

		0.3 mg/L, adult fish exposed for 7,14, and 21 days	i) Fecundity (number of daily egg production) reduced.<br>ii) Secondary sexual features in males (number of papillary processes in the fin rays) remained unaltered.<br>iii) *vtg1* genes in the liver of male and female medaka significantly reduced after 7-, 14-, and 21-days exposure; *vtg2* in male remained unchanged. *vtg2* in female reduced significantly.<br>iv) vtg protein in the liver of male medaka significantly reduced and did not change with time.	Kang et al. (2019)
		10.3, 20.6, 44.9 µg/L; reproductively active adult male and female fish exposed for 21 days for FSTRA following TG 229 guidelines	i) No effect on growth (length and weight), HSI, GSI, and secondary sexual features.<br>ii) Liver vtg content remained unaltered in males while decrease in female at the highest concentration used in	Onishi et al., (2021)

			the experiment (44.9 µg/L).<br>iii) Reproductive activity (fertility rate) impaired at the highest concentration of PCZ used in this study.	
94.	Progesterone (P4)	11, 24, 64, 243 µg/L; adult male and female medaka were exposed for 21 days; FSTRA protocol	i) Sex and concentration-dependent mortality was observed; females were more sensitive to P4 than males.<br>ii) No alteration in growth (length and weight)<br>iii) Reproductive activity (fertility and fecundity) reduced in a concentration-dependent manner.<br>iv) Females developed papillae on the anal fin rays in a concentration-dependent manner.<br>v) No alteration in the liver (HSI) and gonad weight (GSI) in both males and females occurred.<br>vi) Liver vtg content remained unaltered.	Onishi et al., (2021)
95.	Propanil (PRN)	9 mg/L, embryos (3/4 hpf) exposed for 7 days	i) downregulation of cyp*11b*, *3β-hsd*, *gnrhr2*, *cyp19a1b.*	Schiller et al., (2014)

96.	Propylparaben, (PPB)	0.055, 0.55, 5.5, 55 mM; adult medaka males exposed for 1 week.	i) Plasma vtg content increased in a concentration-dependent manner.<br>ii) mRNA expression of *vtg1*,*vtg2*, *chgL*,*chgH*, and *esr1* upregulated while *esr2a* and *ar* remained unaltered.	Inui et al., (2003)
		40,400,1000, and 4000 µg/L; embryos were exposed until 10 dpf, evaluated during embryonic development, evaluated 13,28, and 43 dpf	i) Embryo and larval survivability dependent on concentration and age.<br>ii) Noninflated swim bladder percent increased in a concentration-dependent manner.<br>iii) EROD activity did not alter.	Gonzalez-Doncel et al., (2014a)
		0.311, 0.926, 2.94 mg/L, 16±2 weeks medaka were exposed for three weeks (TG229)	i) Hepatic vtg enhanced in male in a concentration-dependent manner, however in female at enhanced at highest concentration (2.94 mg/L).<br>ii) Secondary sex character in male inconclusive; females no change.<br>iii) The number of total eggs, fertilized eggs and fertility rate reduced at	Kawashima et al., (2022)

			highest concentration (2.94 mg/L).	
97.	6-propyl-2-thiouracil (PTU)	32, 120, 300, 1000 mg/L, embryos (4hpf) exposed until 1dph	i) No effects on hatching and hatching rates.<br>ii) Body length smaller in 120, 300, and 1000 mg/L groups<br>iii) Developmental abnormalities absent, however, reduction in swim bladder inflation occurred.<br>iv) Reduction in eye size (100 mg/L)<br>v) Larval swimming reduced significantly.<br>vi) Significant effect on expression of *tshβ-like* in last two exposure groups (300 and 1000 mg/L)<br>vii) No effect on expression of *tshβ, dio1, dio2, trα* and *trβ*	Horie et al., (2023a)
98.	Ractopamine (RCT)	5, 25, 125, and 625 µg/L, medaka embryos (4hpf) exposed for 44 days.	i) Caused no growth response in medaka (no effect on hatchability, time to hatch, or gross abnormality) (no change in mortality, body weight or total length).<br>ii) Disrupt transcriptional changes of gene	Sun et al., (2016a)

			(especially in females) related to hypothalamic-pituitary-gonadal axis (HPG).<br>iii) Genes involved in anti-oxidative activity or detoxification were affected in a gender-specific manner.<br>iv) *vtg1* and *vtg2* (whole fish) was upregulated in females; *esr1* and *esr2a* increased in females; no change in *vtg*s in males while *erβm* RNA enhanced; no change in *arα* in both males and females.<br>v) All the steroidogenesis regulatory genes including *cyp19a* and *cyp19 b* (except *StAR* gene) are upregulated in females; in males *cyp11a*, *cyp11b* and *cyp17a* slightly increased.<br>vi) *cyp1a1* induced in females, no change in males; *cyp3a* remained unchanged in both male and females; *gst*	

			decreased in females, no change in males.<br>vii) No change in *cat* and *sod* mRNAs in males and females; *gr* was increased, *gpx* was decreased in females, no change in males.	
99.	Raloxifene (RLF)	63.9, 203, 721, 2080 µg/L; Reproductively active adult fish were exposed for 3 weeks (OECD TG 229 protocol).	i) No effect on survivability, growth (length and weight), reproductive activities (fertility and fecundity).<br>ii) Liver weight (HSI) and gonad weight (GSI). remained unaltered.<br>iii) Liver VTG in male fish showed an enhancement while in females, a concentration-dependent decrease (721 and 2080 µg/L) was observed.	Onishi et al., (2021)
100.	Roundup (RND)	0.5 mg/L and 5 mg/L, embryos 8hpf exposed for 15 dpf, depurated until sexual maturity	i) Fecundity and fertilization efficiency did not alter.<br>Brain:<br>i) In females, no change in mRNA expression in *kiss1* and *kiss2*; *gpr54-1* (receptor for kiss 1) and *gpr54-2* (receptor for kiss 2) mRNA levels are down regulated in low	Smith et al., (2019)

			roundup (0.5 mg/L); gpr54-1 downregulated in high roundup groups (5mg/L)<br>ii) In males, *kiss1*, *kiss2*, *gpr54-1* and *gpr 54-2* did not change	
101	Sodium perchlorate (SPC)	100 mg/L<br>Adult fish exposed for 7 days.<br>Maintained at 26°C, 29°C and 33 °C	i) Downregulation of *trα* and *trβ* genes<br>ii) Upregulation of *dio2*<br>iii) Decrease in T4 levels but T3 remained unaltered<br>iv) Fecundity decreased with the increase in temperature	Lee et al., (2014c)
102.	Spironolactone (SPR)	0.05, 0.5, 5, and 50 µg/L; Reproductively active adult male and females were exposed for 21 days	i) GSI enhanced in male fish exposed to 50 µg/L<br>ii) Fecundity significantly reduced in a concentration-dependent manner.<br>iii) Anal fin papillae significantly increased in both male and females<br>iv) *vtg* mRNA content in the liver of female fish significantly decreased in female fish exposed to 50 µg/L	LaLone et al., (2013).

103.	Sulfathiazole (SFT)	50 and 500 mg/L, male medaka exposed for 14 days	v) Serum E2 enhanced in both 50 and 500 mg/L concentrations	Ji et al, (2010).
104.	Surflan (SRF)	EXPOSURE 1: 3.8,5.5, and 8.5 µL/L, 7-month-old male fish exposed for 3 days.<br><br>EXPOSURE 2: 0.67, 1.3, and 2.5 µL/L, 6-month-old male fish exposed for 16 days	i) Induced *chg* (choriogenin) exposed for 3 or 16 days.<br>ii) No induction in liver VTG.	Hall et al., (2005)
105.	Tamoxifen (TAM)	0.01, 0.1, 1, and 8 mg/g diet; adult fish fed for 7 days.	i) Increased plasms VTG level in males and females in a dose-dependent manner.	Chikae et al., (2004)
		1, 5, 25, 125, 625 µg/L,<br><br>EXPOSURE 1: 0-15 dph;<br><br>EXPOSURE 2: Adult fish exposed for 21 days	EXPOSURE 1:<br>i) Hatchability and time to hatching significantly reduced in embryos exposed > 25 µg/L<br>ii) Male biased sex ratio (based on secondary sex characteristics)<br>EXPOSURE 2:<br>i) No difference in HSI and GSI in both males and females.<br>ii) Liver histology altered especially in males.	Sun et al., (2007a)

			iii) Concentration-dependent increase in plasma vitellogenin in males; decrease in females (25 µg/L and above)<br>iv) Fertility and fecundity significantly decreased in 625 µg/L group. Hatching in F1 eggs also significantly decreased in fish exposed to 625 µg/L group (F0).<br>v) Concentration-dependent increase in F1 male populations when F0 fish exposed to 25, 125, 625 µg/L.	
		30, 100, and 300 µg/L, 6-month-old fish exposed for 72 h.	Brain:<br>i) No effect in brain of female about *esr1* or *esr2a* mRNA expression.<br>ii) *arα* expression was significantly increased in the brain of female fish exposed to 100µg/L TAM.<br>iii) *cyp19a* remained unaltered in female brain.	Sun et al., (2011a)

			iv) *cyp19b* expression was suppressed significantly in the brain of females exposed to all concentrations of TAM.<br>v) In male brain, *esr1* expression was increased significantly in a concentration-dependent manner.<br>vi) *arα* mRNA expression was enhanced significantly in the brain of males exposed to 100 µg/L TAM.<br>vii) *cyp19a* was enhanced in the brain of male fish exposed to 30 µg/L TAM.<br>viii) *cyp19b* expression was suppressed in the brain of male fish in all concentrations of TAM.<br><br>Liver:<br>i) expression of *vtg1* and *vtg2*, and *esr1* down regulated in the liver of female fish in a concentration-dependent manner.<br>ii) *Esr2a* upregulated significantly in female	

				liver. however, *arα* in the liver remained unaltered after TAM exposure.<br>iii) In males *vtg1*and *vtg2* enhanced significantly in fish exposed to 30µg/L, however the data are not statistically significant in male fish exposed to 300µg/L TAM.<br>iv) *Esr1* expression was enhanced significantly in male liver at 300 µg/L TAM but *esr2a* remained unaltered. *a*rα expression was enhanced only in 100 µg/L TAM<br><br>Gonad:<br>i) *Esr1*, *esr2a*, and *arα* remained unaltered by TAM in ovary.<br>ii) *StAR*, and *cyp11a* mRNA in ovary enhanced only in 300µg/L TAM.<br>iii) *cyp11b* did not differ significantly in ovary.<br>iv) *cyp17a* remained unchanged in ovary but	

			cyp17b down regulated at 300µg/L TAM.<br>v) *cyp19a* upregulated and *cyp19b* remained unaltered in ovary after TAM exposure.<br>vi) In testis, significant decrease in *esr1* and *esr2a* mRNA was observed in males exposed to 300 µg/L TAM.<br>vii) *StAR* enhanced in males in a concentration-dependent manner.<br>viii) *cyp11a* and *cyp11b* remained unaltered in testis after TAM exposure.<br>ix) *cyp17a* and *cyp17b* down regulated in testis after TAM exposure (*cyp17b* only in 300µg/L).<br>x) *cyp19a* down regulated and *cyp19b* upregulated in testis after TAM exposure.	
		1.32, 2.51,5.08,10.17, 20.44 µg/L; adult fish (F0) were exposed for 4 weeks, F1, 15 weeks, F2, 15 weeks	i) Fecundity reduced in a concentration-dependent manner.<br>ii) The adverse outcome pathway (AOP) -related	Flynn et al., (2017)

		(total 29 weeks) MEOGRT protocol	endpoints were typically affected at a LOEC of 1.3 µg/L<br>iii) Modest increase in VTG concentration in males and reduced in females.<br>iv) Secondary sexual features reduced in XY males mostly in F2 fish.<br>v) Reduced growth in F1 males (XY) and females (XX)	
		10, 28, 83.3 µg/L; reproductively active adult male and female fish were exposed for 21 days (FSTRA protocol following TG 229 guidelines)	i) Survivability of the fish decreased in a concentration-dependent manner.<br>ii) No effect was observed in growth (length and weight), HSI, GSI, and secondary sexual features.<br>iii) Reproductive activity (fertility and fecundity) impaired in a concentration-dependent manner.<br>iv) Liver vtg enhanced in males and reduced in females in a concentration-dependent manner.	Onishi et al., (2021)

	TAM+E2	TAM (10 or 50, or 250 µg/L) + E2 (200 ng/L),<br>5 months old adult exposed for 21 days.	i) Plasma vtg concentration decreased in both males and females.<br>ii) Impaired reproductive success at highest concentration of TAM (250 µg/L) with E2 (200 ng/L)	Sun et al., (2009)
	TAM+ EE2	TAM (30, 100, 300 µg/L) + EE2 (20 ng/L)<br>Adult fish exposed for 72h	Brain:<br>i) In males, *esr1* mRNA level significantly decreased at combined EE2 and TAM exposure in a concentration-dependent manner. While TAM in combination with TAM did not alter the expression of *esr2a* or *arα*<br>ii) *cyp19b* mRNA in male brain in combined exposure significantly decreased with increasing concentration of TAM.<br>iii) In females, combined exposure of EE2 and TAM did not show any significant change in *esr1*; slight induction with *esr2a; arα* elevated	Sun et al., (2011a)

				but not significant; *cyp19b* mRNA level decreased significantly.<br>Liver.<br>i) In males, *vtg1* mRNA was enhanced by EE2 as well as in combination with TAM, while vtg2 mRNA expression by EE2 was significantly blocked by TAM in a concentration-dependent manner.<br>ii) transcription of *esr1* in males enhanced following coexposure with EE2 but *esr2a* remained unaltered; *arα* decreased at highest TAM (300 µg/L) concentration.<br>iii) In females, *vtg1* and *vtg2* decreased with enhanced concentration of TAM in combination with EE2.<br>iv) *esr1* and *arα* decreased with enhanced concentration of TAM, while *esr2a* remained unaltered.<br>Gonad:	

			i) In testis, *arα* induced in 100 µg/L TAM co-exposure; *StAR*, *cyp11a*, *cyp17a*, *cyp17b*, increased in TAM coexposure in a concentration-dependent manner.<br>ii) In ovaries, *arα*, *StAR* and *cyp 11a* increased; *esr1*, *esr2a*, and *cyp19a* no change in combined treatment compared to EE2 alone.	
106.	Terephthalic acid (TPA),	0.01, 0.1, 1, and 10 mg/L, fertilized eggs exposed for 40 days	i) No significant effect on mortality and juvenile survival.<br>ii) The NOEC of 30 dph/survival and reproduction was 10 mg/L.<br>iii) Upregulated *cyp19b*, *star*, *cyp17*, and *cyp19a* mRNA in a concentration-dependent manner. *erα, vtg1, vtg2, cyp11a, hsd3b* mRNAs remained unaltered.	Jang and Ji, (2015)
107.	4-*tert*-octylphenol (4t-OP)	EXPOSURE 1: 100 µg/L<br>begins 1,3, 7,21,35 dph continued until 100 dph	Exposure 1:<br>i) Testis-ova formation enhanced in 1 and 3 dph groups and then gradually reduced (not	Gray et al., (1999a)

		EXPOSURE 2:<br>100 µg/L,<br>1 dph continued 1,2, and 3 months of age<br><br>EXPOSURE 3:<br>100,200, and 300 µg/L<br>Adult males exposed for 6 weeks	significant) with the late exposures (7,21, 35 dph).<br>ii) Varying degree of histological effects on female gonad.<br><br>Exposure 2:<br>iii) Only 6% fish with testis-ova in 3 months exposure group; other two groups (1 and 2 months) did not show any testis-ova.<br>iv) Varying degree of histological effects on female gonad.<br><br>Exposure 3:<br>v) 16% (1 in6 fish) have testis-ova, if exposed for 3 weeks.<br>vi) Abnormal acidophilic spermatogonial tissue was observed in anterior part of the testis.	
		10, 25, 50, 100 µg/L, Males and females are exposed from 1dph to 6 months post hatch	i) Developmental abnormalities in F1 embryos (circulatory system difficulties, incomplete eye development, failure to inflate swim bladder)	Gray et al., (1999b)

			ii)	Reduced courtship activity and fertilization by males (only males exposed to 25 and 50 µg/L).	
			iii)	Low incidence of testis-ova (50 µg/L); males with intersex gonad able to be fertilized eggs produced from unexposed females.	
		20, 50, 100, 300 ppb, adult males exposed for 21 days; females unexposed.	i)	vtg levels are enhanced in male fish serum in a concentration-dependent manner.	
			ii)	Concentration-dependent reduction in egg production and fertility.	
			iii)	Increasing occurrence of developmental abnormalities.	
			iv)	Some males with testis ova (74 and 230 ppb).	
			v)	Spermatogenesis inhibited.	
			vi)	May be genotoxic as well as estrogenic.	
		20, 50, 100, 300 ppb, adult males exposed for 21 days; females unexposed.	vii)	vtg levels are enhanced in male fish serum in a concentration-dependent manner.	Gronen et al., (1999)

			viii) Concentration-dependent reduction in egg production and fertility.<br>ix) Increasing occurrence of developmental abnormalities.<br>x) some males with testis ova (74 and 230 ppb).<br>xi) Spermatogenesis inhibited.<br>xii) May be genotoxic as well as estrogenic.		
		6.25, 12.5, 25, 50, and 100µg/L, Fertilized eggs exposed for 60 days	i) LOEC is 11.4 µg/L (sexual differentiation and hepatic vtg induction).<br>ii) Hepatic vitellogenin increased (11.4µg/L)<br>iii) Sex-ratio skewed (based on secondary sex characters and gonad histology) towards female (48.1 µg/L).<br>iv) Secondary sex character reverted from female to male, however gonad histology did not change, if the fish returned to clean water.	Seki et al., (2003a)	
		13.6, 30.1, 66.1, 145, 320 µg/L, male and	i) Concentration-dependent enhancement occurred in males; no	Nozaka et al., (2004).	

		female fish exposed for 21 days	alteration in females (except highest concentration where an increase was noticed).<br>ii) LOEC for vtg induction in liver of male fish was 64.1 µg/L	
		6.2±0.6, 12.5±1.5, 25.1±2.6, 51.1±5.6, 102.3±8.5 µg/L; adult fish (F0) were exposed for 4 weeks, F1, 15 weeks, F2, 15 weeks (total 29 weeks) MEOGRT protocol	i) Fecundity, fertility, and hatching decreased significantly.<br>iii) the LOEC for fecundity and fertility was 100 µg/L; for hatching 25 µg/L.<br>iv) Liver vtg increased significantly, the LOEC was 6.25µg/L.<br>v) Secondary sexual characteristics decreased significantly (the LOEC was 6.25µg/L).<br>vi) The body weight significantly decreased (LOEC25 µg/L), while the length remained unaltered.<br>vii) Adult survivability significantly decreased.<br>viii) Testicular hypoplasia, malformation of the efferent duct, and	Flynn et al., (2017)

			oocytes observed in testis (LOEC=13µg/L).<br>ix) Basophilia observed in liver (LOEC=51 µg/L).<br>x) Histopathological changes induced in kidney.	
		6.2,12.5,25,50,100 µg/L,<br>4hpf embryos exposed until 8 dpf (stage 38)	i) LOEC for intersex development is 23.7 µg/L.<br>ii) No significant difference in gsdf expression in XY embryos.	Horie et al., (2022a)
		25.3, 82.3, 250 µg/L, adult fish exposed for 3 weeks (TG 229)	i) Liver vtg enhanced in male (last two doses (82.3, 250 µg/L) no change in females.<br>ii) Secondary sex characters unchanged in both males and females.<br>iii) Number of total eggs, fertilized eggs and fertility rate remained unaltered.	Kawashima et al., (2022)
108.	4-*tert*-pentylphenol (4t-PP)	62.5, 125, 250, 500, and 1000 µg/L,<br>12 hpf embryos exposed until 60dph, 70 dph, 101 dph.<br><br>F1 hatchlings exposed (62.5, 125,	i) The 60 d LC_{50} is 693µg/L.<br>ii) Growth reduced significantly in 1000 µg/L group.<br>iii) Appearance of secondary sexual features in males are	Seki et al., (2003b)

		250 µg/L) until 61 dph.	reduced in a concentration-dependent manner.<br>iv) Testis ova seen in 250 µg/L and above; no histological changes observed in ovary.<br>v) GSI in male and female treated groups showed no significant difference with controls.<br>vi) Hepatic vtg in males increased in almost all groups, no change in females.<br>vii) Testis-ova in F1 fish enhanced in a concentration-dependent manner.<br>viii) Hepatic vtg in F1 fish enhanced in males remained at the same level in females.<br>ix) LOEC 931 µg/L (lethal and sublethal toxicity, growth inhibition).<br>x) LOEC for estrogen effects 224 µg/L (abnormal sexual differentiation; <51.1 µg/L (hepatic vtg induction).	

			xi) LOEC for reproductive impairments 224 µg/L<br>xii) LOEC for F1 is 224 µg/L (sublethal toxicity), <51 µg/L (hepatic vtg induction).<br>xiii) Female biased sex ratio.	
		62.2, 121, 238, 413, and 783 µg/L; fertilized eggs were exposed until 60 dph	i) sex reversal (XY male to female) occurred in a concentration-dependent manner.<br>ii) No sex reversal occurred in XX female.<br>iii) Complete inhibition of *p450*$_{11\beta}$ mRNA in the gonad of XY-sex reversed medaka	Yokota et al., (2005)
		96.5,342,1100 µg/L; reproductively active adult male and female fish were exposed for 21 days (FSTRA following TG 229 guidelines)	i) Although survivability of the fish was concentration-dependent, no effect was observed on growth (length and weight), HSI, GSI, and secondary sexual features in both male and female fish.<br>ii) Reproductive activity (fecundity and fertility) impaired in a concentration-dependent manner.	Onishi et al., (2021)

			i) vtg content in the liver of male and female fish enhanced in a concentration-dependent manner	
		58.4, 227, 940µg/L,<br><br>adult fish exposed for 3 weeks (TG229 assay)	ii) Concentration dependence enhancement of liver vtg in males; significant enhancement at highest concentration (940 µg/L) in females.<br>iii) HSI enhanced in males at higher concentrations (940 µg/L).<br>iv) No change in secondary sex characters in both males and females<br>v) Number of total eggs, fertilized eggs, decreased at highest concentration (940 µg/L) and fertility rate is tended to decrease at highest concentrations (940 µg/L).	Kawashima et al., (2022)
109.	Testosterone (T)	100 µg/L stage 10 embryo and 1, 7, and 21 dph larvae exposed for 6 days and observed after 5 months.	i) No significant change in sex ratio.<br>ii) Newly hatched fry or one week post hatch larvae displayed intersex gonad.	Koger et al., (2000)

110.	Tetrabromobisphenol A (TBBPA)	32,100, 320, and 1000 µg/L; 4 hpf embryos were exposed until 9 dpf	i) No effect on hatching as well as swim bladder inflation.<br>ii) No effect on swimming behavior<br>iii) Concentration-dependent enhancement in t*shβ* and *tshβ-like* gene expression<br>iv) Downregulation of *trα* and *trβ* gene expression, though not concentration-dependent<br>v) no effect on the expression of *dio1* and *dio2*.	Horie et al., (2023a)
111.	1,2,5,6-tetrabromocyclooctane (TBCO)	58, and 607 µg/g food, adult fish fed for 21 days	i) Cumulative fecundity was 18% reduced in fish fed with 58 µg (no effect in 607 µg/g).<br>ii) HSI and GSI did not alter either in males or females.<br>Brain:<br>iii) No change in the gene expression pattern of *esr1, esr2a, arα, neurpcpY, cGnRH II, mfGnRH, sGnRH, GnRH R1, GnRH RII, GnRH RIII, gthα, lhβ, cyp19b* in male and	Saunders et al., (2015)

			female fish fed with 58 or 607 µg/g.<br>Gonad:<br>iv) *esr1*, *esr2a, arα, HDLR, HMGR, StAR, cyp17, inhibinA, activinBA* are downregulated in testis either in 58 or in 607 µg/g food or in both concentrations.<br>v) *esr1*, *esr2a, arα. HDLR, LDLR, StAR, inhibinA, acivin BA* are down regulated in ovary either by 58 or 607 µg/g food or in both concentrations.<br>Liver:<br>vi) *chgHm* and *cyp3a* upregulated in males fed only with 58 µg/g food.<br>vii) *esr1*, *vtg2*, *chgH* are upregulated and *esr2a* and *annexin 2* are downregulated in females fed only with 58 µg/g food.	
		10,100, 1000 µg/L; embryos exposed 8-13 dpf (until the first day of post hatch)	i) TBCO accumulated in the embryos.<br>ii) Did not increase incidence of mortality	Sun et al., (2016c)

			or deformities in embryos.<br>iii) A concentration-dependent increase in number of days to hatch and decrease in success of hatching.<br>iv) Among 20,425 genes, 240 genes are upregulated and 99 are downregulated in embryos exposed to 100 µg/L concentrations.<br>v) Among 1256 unique proteins, 68 proteins were upregulated, and 121 proteins were downregulated.<br>vi) Transcriptome and proteome analysis indicated TBCO impair vision and contraction of cardiac muscle.	
		40.6 µg/g food and 1034.4 µg/g food (F0) and the produced embryos reared to F1, F2, and F3 generations without any further exposure	i) Concentration of TBCO in eggs produced by F0 is 711.3 ng/g from 40.6 µg/g (low fed group) while 2535.5 ng/g from 1034.4 µg/g (high fed group).<br>ii) No change in length of the F1, F2 and F3 fish. HSI increased and GSI	Devoy et al., (2023)

				significantly decreased in F3 fish fed with high diet.<br>iii) Heart rate decreased significantly in F1 embryos fed with low and high TBCO, swim bladder inflation and spinal curvature enhanced in high fed group. No change in F2 and F3 embryos.<br>iv) Fecundity in F1 reduced by 33.9 (40.6 µg/g food) and 33.3% (1034.4 µg/g food). In F2 no reduction in low fed group but 29.8% reduced in high fed group.<br>v) In F1 females, *lhr* mRNA was increased while *fshr* mRNA decreased significantly in ovary; however, no alteration in *fshr* and *lhr* mRNAs were observed in F2 and F3 generations.<br>vi) *cyp11a* showed a dose-dependent enhancement and *cyp19a*, mRNAs showed significant	

			increase in low feed group in ovary of F1 fish but not in F2 and F3 generations.<br>vii) No difference observed in the serum E2 levels or the *vtg* mRNA in liver of F1, F2 and F3 female fish	
112.	2,2′,4,4′ tetrabromodiphenyl ether (BDE-47).	0.5, 1, 5 and 10 mg/L ; fertilized eggs (stages 10-11) assessed after 76, 124, 196, 244, and 316 hpf after exposure	i) Did not induce any significant embryo mortality, hatching failure, or delay or precocious hatching.	Gonzalez-Doncel et al., (2014b)
		10, 100, 1000 ng/g diet, 4dph exposed for 40 days depuration 80 days	ii) Growth rate increased at highest dose (1000 ng, 70 days after exposure.<br>iii) No difference in number of eggs produced or fertility rate.<br>iv) Minor effects on thyroid histology.<br>v) Liver and ovary histology did not show any significant difference.<br>vi) No difference in sex ratio.	Gonzalez-Doncel et al., (2016)
		1000 ng/g diet	i) BDE-47 detected in the embryos (maternal transfer) concentration	Gonzalez-Doncel et al., (2017)

		Reproductively active adult medaka were fed for 40 days	increased with the duration of exposure.<br>ii) Reproductive capacity, hatching, swim bladder inflation was not affected by parental exposure to BDE-47<br>iii) The locomotor activity and the lipid content of the larvae did not alter after BDE-47 exposure.	
		1000 ng/g food; 6 dph larvae exposed for 140 days.	i) The bioaccumulation of BDE-47 in whole body tissue of medaka enhanced with duration of exposure (significantly higher in males than females), while no effect was observed on the survivability and growth of the fish.<br>ii) Phenotypic sex-ratio remained unaltered.<br>iii) No effect on fertility and fecundity.	Beltran et al., (2022)
113.	Thiourea	0.001, 0.003, 0.01,0.03%<br>Adults were incubated (7-10 days), the spawned eggs were used for	i) The minimum effective dose for decreasing thyroid hormone concentration in eggs and adults is 0.03%	Tagawa and Hirano (1991)

		hormone concentration	ii) No effect in hatching and survival of the embryos<br>iii) No effect on length and weight of the larvae after until 16 dph	
114.	Thyroid hormone (T3)	T3:<br>10 nM, embryos exposed until 10 dpf (when swim bladder inflation is complete)	iv) No mortality<br>v) No swim bladder inflation in males and females; decrease in the surface area of swim bladder in females.<br>vi) No change in gene expression in females and males.	Godfrey et al., (2019)
		T3:<br>0.12,0.25,0.5, 1mg/L Embryos (2 hpf) exposed until 4hph (0 dph) 9 total exposure time 220h)	i) No inhibition in swim bladder inflation.<br>ii) Upregulation of *trα* and *trβ* mRNAs in a concentration-dependent manner	Horie et al., (2022d)
115.	17α-trenbolone (TRA)	1.3, 3.9,12, 38, and 120 ng/L, adult fish (17 weeks old) exposed for 21 days	i) Did not adversely affect survival and fecundity; NOEC=110 ng/L and LOEC > 110 ng/L.	Robinson et al., (2017)
116	17β-trenbolone (TRB)	10, 50 ng/L, 1 dph fries exposed for 38 days and 60 days.	i) sex ratio did not change.<br>ii) sperm area in testis increased in 50 ng/L fish exposed for 60 days.<br>iii) vtg in whole fish induced after 38 days exposure (50 ng/L.	Orn et al., (2006)

		50, 500, and 5000 ng/L; reproductively active adult males and females were exposed for 21 days	i) No mortality observed during the exposure period.<br>ii) GSI of male fish did not affect; in females a trend of concentration-dependent decreased was observed (not significant)<br>iii) In secondary sex characters the number of anal fin papillae did not significantly differ in males, but in females, a concentration-dependent increased in number of anal fin papillae was seen.<br>iv) VTG levels in females decreased significantly in a concentration-dependent manner; no effect was observed in males.	Seki et al., (2006)
		50,500, 5000 ng/L, 4 months old exposed for 7 days	i) GSI in females and HSI in males enhanced in fish exposed to 500 ng/L<br>ii) Up-regulation of brain *gnrhR2* and *cyp19b* (concentration-dependent) in female fish.	Zhang et al., (2008b)

			iii) Down regulation of *StAR* and *cyp11b* mRNA in testis (concentration-dependent)<br>iv) Upregulation of *cyp19a* and down regulation of *HDLR* in ovary (concentration-dependent)<br>v) Down regulation of *esr1* in liver of male and down regulation of *vtg1*, *vtg2*, *chgH*, *chgHm* mRNAs in the liver of both male and female fish.<br>vi) Up regulation of *cyp3A* and *annexin max2* mRNAs in liver of female fish.	
		50, 500, and 5000 ng/L, 12-14 weeks old exposed for 7 days	i) HSI enhanced in females exposed to 50 ng/L, GSI enhanced in 500 ng/L; in males HSI affected in fish exposed to 500 ng/L.<br>ii) Fecundity reduced in a concentration-dependent manner.<br>iii) Induced *ar* expression in ovaries but not in	Park et al., (2009)

				female liver (5000 ng/L).	
		1.0 µg/L,<br>8-month-old fish exposed for 21 days	i)	14-21 days exposure of female medaka did not induce masculinization in anal fin	Grillitsch et al., (2010)
		13±2 and 31±5 ng/L; F0 adult fish exposed for 14 days; F1 embryos exposed for 8 weeks	i)<br>ii)<br>iii)	In F1 fish, concentration-dependent sex reversal of the XX fish with ovo-testis (XX males).<br>Expression of *sox9b* and *protamine* was higher in testis than ovary; and expression of *fig1α* and *zpc1* was higher in ovary than testis (F1 fish).<br>In intersex gonads, all four gene expressions was in between the levels seen in ovaries and testis (depending on the sex-reversal process).	Flynn et al., (2013)
		5 ng/L,<br>15 dpf larvae exposed for 10 days (15-25 dpf)	i)	Downregulation of ovarian structure protein (*osp1*) expression in females [osp1 was observed only in ovaries and localized in the cytoplasm and follicular	Abdel-Moneim et al., (2015)

			layer of immature and mature oocytes.<br>ii) vtg did not show any significant changes in females.<br>iii) Ovo-testis appeared in females.	
		EXPOSURE 1:<br>2, 6, 20, 60, 100, and 200 ng/L; 12 hph larvae exposed until 60 dph<br><br>EXPOSURE 2:<br>2,6,20,60, 100, 200 ng/L; 12 hph larvae exposed until 7dph.	EXPOSURE 1:<br>i) Sex-reversal occurred (XX females to phenotypic males) in a concentration-dependent manner.<br>ii) Fertility and fecundity affected either males exposed to TRB or females exposed to TRB.<br>iii) GSI in males did not significantly differ, however, in females GSI significantly increased in a concentration-dependent manner with swollen abdomens.<br>EXPOSURE 2<br>i) Microarray analysis identified 117 upregulated and 32 downregulated genes (total 149) after TRB exposure.	Mazukami-Murata et al., (2015)

			ii) Pathway analysis indicated the upregulated genes belonged to metabolism of lipids, terpenoids, and polyketides (most of the genes showed concentration-dependent upregulation).<br>iii) Among the downregulated genes, peroxisome proliferator activator (PPAR) signaling pathway genes were identified.	
		2.2±0.5, 5.1±1.2, 12.9±2.7, 31.7±4.8, adult fish (F0) were exposed for 4 weeks, F1, 15 weeks, F2, 15 weeks (total 29 weeks) MEOGRT protocol 84.2±13 ng/L;	i) Fecundity reduced significantly in a concentration-dependent manner (LOEC 32 ng/L).<br>ii) Fertility reduced significantly (LOEC 13 ng/L).<br>iii) No effect on hatching (LOEC > 84 ng/L).<br>iv) Liver vtg reduced significantly (LOEC 32 ng/L).<br>v) Significant enhancement in secondary sexual features (LOEC 32 ng/L).	Flynn et al., (2017)

			vi) No effect on length and weight of fish as well as adult survivability (LOEC > 84ng/L).<br>vii) Presence of anal fin papillae in XX fish.<br>viii) XX fish have functional testis.	
		5µg/L, adult fish exposed for 7,14, and 21 days (TG 229, modified)	i) Fecundity (number of daily egg production) reduced.<br>ii) Secondary sexual features in males (number of papillary processes in the fin rays) remained unaltered.<br>ii) *vtg1* and *vtg2* genes in the liver of males and females significantly reduced after 7-, 14-, and 21-days exposure.<br>iii) VTG protein in liver reduced significantly and did not change over time.	Kang et al., (2019)
		8.03, 26.8, 84.6, 291 ng/L; reproductively active adult male and female medaka exposed for 21 days for FSTRA following TG229 guidelines	i) No effect was observed on survivability, growth (length and weight), HIS, GSI, and liver VTG content of both male and female fish.	Onishi et al., (2021)

			ii)	The number of anal fin papillae significantly increased in female fish in a concentration-dependent manner; no effect was observed in male fish.	
			iii)	The reproductive activity (fertility and fecundity) impaired in a concentration-dependent manner.	
		0.32,1, 3.2, 10, 32µg/L, 4hpf exposed for 60 dph	i)	All XX medaka exposed to 0.5, 1.92, 6.32 and 22.1 µg/L showed ovotestis (female to male sex reversal).	Horie et al., (2022a)
			ii)	*gsdf* expression in XX embryos in the 6.32 and 22.1 µg/L group increased.	
117.	Triadimefon (TRF)	EXPOSURE 1:<br>2 and 3.5 µM; adults (7 months old) exposed for 3 and 7 days<br>EXPOSURE 2:<br>2, 3.5, and 5 µM; adult males exposed for 3, 17 and 14 days	i)	Significant increase of cyp1a and cyp3a enzyme activity	Lin et al., (2014)
			ii)	Induction of cyp1a was concentration-dependent	
			iii)	Expression of *cyp3a38* and *cyp3a40* was induced after 3 days exposure	
		1,5,10,25, 50 µg/L; adult fish exposed for 28 days	iv)	concentration-dependent reduction in fecundity and fertility	Liu et al., (2018)

			v) No hatching delay	
118.	Triadimenol (TRD)	3, 30, 300,600,1200,2400, 3000 µg/L, 0 dpf fries exposed for 35 days, depuration 3 months	i) Did not induce significant teratogenic effects or oxidative stress in embryos and hatchlings.<br>ii) Delayed hatchability and hatching time (600-3000 µg/L)<br>iii) Decreased mRNA levels of *vtg2* (30-300µg/L) in liver and increased *cyp3a40*, but not *cyp3a38*, *vtg1*, *cgh* and *erα* (3-300 µg/L), *cyp1a* (300 µg/L) was down regulated (not significant)<br>iv) Altered ovary development (3,30, 300 µg/L) and reproduction.	Chu et al., (2016)
119.	Tributyltin (TBT)	0.1, 0.26, 0.64, 1.6, 4 µg/L; adult male and females exposed for 21 days	i) HSI in males increased in a concentration-dependent manner.<br>ii) Liver vitellogenin in male and females remained unaltered with TBT exposure.	Nozaka et al., (2004)
		0.7 µg/L, fries were exposed for 5,9, and 14 days	i) No change in adult sex ratio.<br>ii) Decrease in 5 days but no significant change in	Kuhl and Brouwer, (2006)

			brain aromatase (*cyp19b*) in 14 days.<br>iii) Aromatase enzyme activity decreased on 14 days exposures.	
		0.16, 0.8, 3.96, 19.2, 82.1 ng/egg.<br>Embryos were injected with TBT and evaluated 60 dph	i) Concentration-dependent mortality impaired embryonic development.<br>ii) No masculinization or feminization in genetic (XX) females or males (XY).	Hano et al., (2007)
		1µg/L, adult fish exposed for 3 weeks	i) Estrogen-related receptors (ERR) enhanced in testis.	Zhang et al., (2008d)
		EXPOSURE 1: F0 fish<br>0.32, 1, 3.2, 10 µg/L, adults (14 weeks old) exposed for 21 days (TG210)<br><br>EXPOSURE 2: 0.32 and 1 µg/L,<br>F1 eggs (collected from F0) exposed until 30 dph (TG 229)	F0<br>i) Concentration-dependent increase in mortality.<br>ii) Concentration-dependent decrease in fecundity and fertility.<br>iii) No significant difference in hepatic vitellogenin content though tended to be lowered in 3.2 µg/L.<br>iv) No effect on gonad histology (both testis and ovary).<br>F1:	Horie et al., (2018)

			i) No effect on the survivability and hatching of the embryos and survivability of the larvae.<br>ii) The length and weight of the larvae reduced significantly.<br>iii) No effect on gonad histology (both males and females)<br>iv) No difference in sex ratio.	
		250,500,1000,2000 ng/L, 4hpf embryos exposed until 8dpf (stage 38)	i) *gsdf* expression in XY males are significantly decreased from control XY male embryos (stage 38) exposed only to 250 µg/L.<br>ii) In XX embryos *gsdf* expression was significantly increased exposed only to 250 µg/L.	Horie et al., (2022a)
120.	Triclosan (TCS)	1,10, 100. 500 and 1000 µg/L,<br>2dph fries exposed for 14 days, depurated for 2 months	i) 1mg/L was lethal to hatchlings within 24h; 0.5 mg/L was lethal after 3 days.<br>ii) 48 h LC50 for fry is 352±68 µg/L.<br>iii) Dorsal and anal fin length also remained unaltered.	Foran et al., (2000)

			iv) Potentially a week androgen.	
		EXPOSURE 1:<br>Embryo<br>78,156,313,625,1250, 2500 µg/L<br>Embryos (24 hpf) exposed for 14 days.<br><br>EXPOSURE 2:<br>Larvae<br>78,156, 313, 625, 1250, and 2500 µg/L<br>1 dph larvae exposed for 96 h<br><br>EXPOSURE 3<br>Adult male/females<br>20, 100, 200 µg/L, exposed for 21 days	Exposure 1: embryos.<br>i) The calculated 96 h LC50 value was 399 µg/L.<br>ii) Mortality of the embryos was time-and concentration dependent.<br>iii) Hatchability decreased in a concentration-dependent manner.<br>Exposure 2: Larvae<br>i) The 96 h LC50 was 602 µg/L.<br>Exposure 3: Adults<br>i) Body length of females reduced in a concentration-dependent manner.<br>ii) No significant difference was observed in fertility and fecundity.<br>iii) HSI of male fish showed concentration-dependent enhancement while in females an increasing tendency was noticed.<br>iv) Concentration-dependent enhancement	Ishibashi et al., (2004)

			in the GSI of both male and female fish.<br>v) Hepatic VTG in males increased in 20 and 100µg/L groups.<br>vi) F1 fish (no direct exposure) obtained from F0 adults grown until 90 dph, did not show any mortality; no significant difference was observed in hatching, body length and weight (after 90 days of development).<br>vii) Sex ratio in F1 fish (no treatment in F1 generation) did not change.	
		1.4, 2.8, 5.6, 11, and 23 µg/L, MEGORT protocol (exposure period 182 days)	i) NOEC is 11 µg/L (based on effects on growth).<br>ii) No impact on hepatic vitellogenin, secondary sex characteristics, sex ratio.<br>iii) Histological observations indicate a toxic or stress effect.<br>iv) No thyroid gland related findings in triclosan-treated males or females were observed.	Mihaich et al., (2019)

			v) Triclosan does not act as an agonist or antagonist within estrogen, androgen, thyroid or steroidogenic pathways.	
		50, 100, 200 µg/L Embryos (8hpf) until 15 dpf	i) Enlarged yolk sac, decreased head trunk angle (HTA), and severe edema in pericardial region.<br>ii) The male ratio increased in a concentration-dependent manner.<br>iii) Expression of *cyp19a1a* and *ar* mRNAs decreased.<br>iv) Global hypomethylation observed in whole body not in isolated primary germ cells (PGC) by 50 µg/L group.<br>v) *Dnmt1* and *dnmt3aa* expression was decreased in both whole body and PGCs when the embryos exposed to 50 µg/L TCS.	Song et al., (2020).
		54.4,104, 177, 353 µg/L, Adult fish (16±2 weeks) exposed for 3 weeks (TG229)	i) Hepatic VTG in male reduced in highest two doses (177 and 353 µg/L) and enhanced in females in highest two	Kawashima et al., (2022)

			doses (177 and 353 µg/L).<br>ii) Secondary sex characters unaltered in both male and females.<br>iii) Number of total eggs, and fertilized eggs reduced at highest concentrations (353 µg/L) fertility rate remained unaltered.	
121.	Tricresyl phosphate (TCrP)	657.9, 1511, 4042 ng/L,<br>0 dph larvae were exposed until 100 dph	i) Significant concentration-dependent inhibition of fertilization of eggs (exposed males mated with unexposed females)<br>ii) Induction of intersex was concentration dependent.<br>iii) Dilation of the efferent duct of the testis<br>iv) Concentration-dependent suppression of 11-KT and testosterone levels in plasma and enhanced E2 level in plasma of male fish<br>v) No change in the *vtg1* and *vtg2* mRNAs in the liver of male medaka.	Chen et al., (2022)

122.	Trifloxystrobin (TRA)	0.1,1, 10, and 100µg/L, embryos exposed for 28 days	i) Hatchability and time to hatching significantly differ between control and 100 µg/L.<br>ii) Larval mortality increased significantly in 0.1, 1. and 10 µg/L group.<br>iii) *esr* mRNA level significantly upregulated above 1µg/L.<br>iv) *vtg* mRNA level upregulated 0.1 and 1 µg/L groups.<br>v) *cyp17* and *cyp19a* are upregulated in 0.1,1, and 10 µg/L groups (100µg/L unaltered).<br>vi) *trα, dio2, ahr* did not show any significant difference.<br>vii) *cyp1a* enhanced in all treatment groups (0.1-100µg/L).	Zhu et al., (2015)
123.	Trilostane (TRI)	100, 300, 1000 µg/L, Newly hatched larvae exposed until 28 dph	i) Decreased the body length in males (1000 µg/L).<br>ii) *vtg1* and *vtg2* significantly increased in males but decreased in females (1000 µg/L).	Sun et al., (2014).

			iii) Both *esr1* and *arα* significantly increased in males, did not change in females; no change in *esr2a* in both males and females.<br>iv) No effect in *fshr*, however, *lhr* mRNA increased in males.<br>v) Significantly increased the transcription of *StAR, 3β-hsd, 20β-hsd, cyp11a*, *cyp11b*, *cyp17a*, *cyp17b*, and *cyp19a* in males.<br>vi) In females no change in *fshr* and *lr* mRNAs and induced the transcription of *StAR* and inhibited the expression of *cyp17b*, *cyp19a*, *cyp19b*, and no change in *3β-hsd, 20β-hsd, cyp11a*, *cyp11b,* and *cyp17a* mRNAs.	
124.	Triphenyl phosphate (TPhP)	1.6, 8, and 40 µg/L 0 dph larvae (pMOSP1-EGF transgenic) were exposed for 100 days (long-term exposure);	i) Significant retardation of ovaries in females occurred in a concentration-dependent manner in long-term exposure.<br>ii) Fecundity reduced in a concentration-dependent	Li et al., (2019)

		3-month-old females exposed for 21 days. (short-term exposure)	manner in long-term exposure.<br>iii) Hepatic *vtg* mRNA downregulated and plasms E2 levels significantly reduced in a concentration-dependent manner in long-term exposure.<br>iv) No ovary retardation was observed in females after short-term exposure.<br>v) Plasma E2 and testosterone level significantly increased in a concentration-dependent manner, while 11-KT remained unaltered in females in short-term exposure.<br>vi) No variation in *vtg1* expression and downregulation of *vtg2* mRNA in liver of females during short-term exposure.	
		2.13, 7.19, 17.1, 44.9 µg/L, 16±2 weeks fish were exposed for three weeks (TG229)	i) Hepatic vtg unchanged in male; in female reduced in last three doses (7.19, 17.1,44.9 µg/L)	Kawashima et al., (2022)

			ii) HSI enhanced in males in 7.19, 17.1, 44.9 µg/L concentrations.<br>iii) Secondary sex characters in both males and females unchanged.<br>iv) Number of total eggs, fertilized eggs, decreased in highest concentrations (44.9 µg/L), fertility rate remained unchanged.	
125.	Triphenyltin chloride (TPT-Cl)	1.6, 8, 40, 200, and 1000 ng/L, adult fish exposed for 5 weeks	F0:<br>i) Spawning frequency and number of eggs produced, and the egg protein content/egg decreased in a concentration-dependent manner, while fertilization rate tended to decrease (significant only in 200 ng/L).<br>ii) Ovarian histology indicates retarded ovarian growth (1000 ng/L).<br>iii) Expression of *vtg1* and *vtg2* mRNAs in the liver of female fish decreased in a concentration-dependent manner.	Zhang et al., (2008e)

			iv) Expression of *17β-hsd1*, *cyp19a* in the ovary suppressed (200 ng/L).<br>v) Expression of cyp19b in the brain of females suppressed (200 ng/L).<br>vi) Expression of *cyp1a*, *cyp2a1*, *ugt2a3* and *17β-hsd1* increased in the liver of both male and female fish (200 ng/L).<br>F1:<br>i) Concentration-dependent increase in the number of hemorrhaging embryos.<br>ii) Growth retardation in hemorrhaging embryos with ocular developmental defects (eye lessness). observed (8 ng/L or higher)<br>iii) Larval morphological abnormalities are also concentration dependent.	
		250,500,1000,2000 ng/L, embryos (4hpf) exposed to until 8dpf (stage 38)	i) *gsdf* expression in XX and XY embryos remained unaltered.	Horie et al., (2022a)
126.		0.019 mg/L,	i) No mortality	Godfrey et al., (2019)

	Tris(1,3-dichloro-2-propyl) phosphate (TDCPP)	Embryos exposed 10 dpf (until swim bladder inflation is complete)	ii) body length in males and females enhanced.<br>iii) significant number of females failed to inflate swim bladder.<br>iv) *vtg* expression increase in females.<br>v) Upregulation of *trα, vtg* and *esrβ* in males.	
		0.12, 0.25, 0.5, 1, and 2 mg/L;<br>Fertilized eggs (2 hpf) were exposed until 4hph.	i) Swim bladder inflation was inhibited (LOEL: 1mg/L)<br>ii) No change in the expression of *tshβ*, *trα*, and *trβ*	Horie et al., (2022d)
127.	Vinclozolin (VIN)	2500 µg/L,<br>1dph fries exposed for 100 dph	i) Affected spermatogenesis in males, no testis-ova.<br>ii) No difference in sex ratio.	Kiparissis et al., (2003b)
		64,200, 640 µg/L,<br><br>EXPOSURE 1: Adults (98 dpf) were exposed for 21 days.<br><br>EXPOSURE 2: Juveniles (40 dpf) exposed for 28 days	i) No significant effect on the number of eggs and fertilized eggs produced either in adult or juvenile test.<br>ii) Decrease in fertility was observed in both adult and juvenile test (significant in adult test at 640 µg/L, but not in juvenile test).<br>iii) Significant decrease in female liver vtg	Nakamura et al., (2014)

			concentration in 640 µg/L only in adult test.<br>iv) No sex reversal<br>v) No significant effect on the number of male papillary processes in adult test; however, in juvenile test, at 640 µg/L concentration decrease in male papillary processes were observed.<br>vi) No female samples develop papillary processes either in adult of juvenile test.	
		100, 300, and 1000 µg/L<br>Adult males were exposed for 72h	i) No mortality.<br>ii) Significant decrease in hepatic vtg1 male fish.<br>iii) Hepatic vtg2 gene remained unaffected.<br>iv) Transcription of esr1 and esr2a remained unaffected.<br>v) In brain upregulation *ofesr1, esr2a, arα* and *cyp19a* and *cyp19b* genes<br>vi) In testis, only esr1 and cyp17b mRNAs were upregulated, while	Sun et al., (2016b)

			cyp19a and cyp19b showed downregulation.	
		17.3, 33.2, 69.5, 136.3, 253.3 µg/L adult fish (F0) were exposed for 4 weeks, F1, 15 weeks, F2, 15 weeks (total 29 weeks) MEOGRT protocol	i) Fecundity and fertility reduced significantly.<br>ii) Vtg in male fish enhanced significantly (LOEC= 136 µg/L).<br>iii) Secondary sexual characters reduced (LOEC=33 µg/L).<br>iv) Length (LOEC 70 µg/L) significantly enhanced.<br>v) Weight (LOEC 33αg/L) significantly enhanced.	Flynn et al., (2017)
		14.5, 43.9, 137, 453 µg/L; reproductively adult male and female fish exposed for 21 days for FSTRA following TG 229 guidelines	i) No effect on mortality, growth (length and weight), HSI, GSI, and secondary sexual features.<br>ii) Liver VTG content in males remained unaltered, while in female significant decrease was observed in fish exposed to highest concentration (453 µg/L) of VIN.<br>iii) Reproductive activity (only the fertility rate) reduced in fish exposed to highest concentration of VIN (453 µg/L).	Onishi et al. (2021)

128.	nano Zinc Oxide (nZnO)	5 µg/mL, 5-8 months old, males exposed for 7 days, females exposed for 10 days	i) Impaired sexual and territorial behaviors in males.<br>ii) Affected fertility by altering sperm viability and motility.<br>iii) Induced reactive oxygen species in ovaries.<br>iv) Induced follicular growth arrest, atresia, and subfertility.<br>v) Altered sex-steroid levels in females,	Paul et al., (2021)
	Zinc sulphate (ZnSO4)	5 µg/mL, 5-8 months old, males exposed for 7 days, females exposed for 10 days	i) Impacted sexual behavior in males.<br>ii) Adversely affected the fertility of females by reducing the number of post-vitellogenic oocytes.	Paul et al., (2021)
